# Classicality of derived Emerton–Gee stack

**DOI:** 10.1007/s00208-025-03259-7

**Published:** 2025-08-15

**Authors:** Yu Min

**Affiliations:** Department of Mathematics, Imperial College London, London, UK

## Abstract

We construct a derived stack $\mathcal {X}$ of Laurent *F*-crystals on , where $\mathcal {O}_K$ is the ring of integers of a finite extension *K* of $\mathbb {Q}_p$. We first show that its underlying classical stack $^\textrm{cl}\mathcal {X}$ coincides with the Emerton–Gee stack $\mathcal {X}_\textrm{EG}$, i.e. the moduli stack of étale $(\varphi ,\Gamma )$-modules. Then we prove that the derived stack $\mathcal {X}$ is classical in the sense that when restricted to truncated animated rings, $\mathcal {X}$ is equivalent to the sheafification of the left Kan extension of $\mathcal {X}_\textrm{EG}$ along the inclusion from the classical commutative rings to animated rings.

## Introduction

Recent years have seen a lot of progress on the categorification of the (arithmetic) local Langlands correspondence, e.g. [6, 10, 13, 14, 19, 38]. In particular, various methods have been used to construct the moduli stacks of Langlands parameters. When $l\ne p$, the moduli stack of local Langlands parameters turns out to be a quotient of a local complete intersection ring, which implies the derived stack of local Langlands parameters in this case is indeed classical. When $l=p$, the moduli stack of local Galois representations is no longer the right geometric object to consider. Instead, one needs to look at the moduli stack $\mathcal {X}_\textrm{EG}$ of étale $(\varphi ,\Gamma )$-modules, constructed by Emerton–Gee in [12]. It is then tempting to ask if there is a well-defined derived version of $\mathcal {X}_\textrm{EG}$ and if it is also classical.

On the other hand, the derived stack of global Langlands parameters is rarely classical. In order to study a global-to-local map of moduli stacks of Langlands parameters, it is then desirable to have a derived version of the Emerton–Gee stack. And this derived extension should have reasonable (co)tangent complexes as suggested by [13, Remark 9.2.3].

In this paper, we will propose a construction of a derived Emerton–Gee stack in the ${\textrm{GL}}_d$-case and investigate its classicality. Note that among all the derived extensions of the Emerton–Gee stack, there is an initial one, i.e. its left Kan extension from the classical commutative rings to animated rings. By classicality of a derived stack, we mean essentially it is the left Kan extension of its underlying classical stack. But usually, the left Kan extension of a stack, which is constructed abstractly, does not enjoy natural moduli interpretations. So in other words, our task is really to find a good moduli interpretation of the left Kan extension of the Emerton–Gee stack.

Let us first revisit the Emerton–Gee stack. Let *K* be a finite extension of $\mathbb {Q}_p$ and $\mathcal {O}_K$ be its ring of integers. By [4] and [36], we now know that there are natural equivalences between the category  of Laurent *F*-crystals on the absolute prismatic site  of $\mathcal {O}_K$, the category of étale $(\varphi ,\Gamma )$-modules and the category of finite free $\mathbb {Z}_p$-representations of the absolute Galois group $G_K:={\textrm{Gal}}(\bar{K}/K)$. Instead of defining a moduli stack of étale $(\varphi ,\Gamma )$-modules as in [12], one can try to construct a moduli stack of Laurent *F*-crystals. Indeed, our first main result says that the two stacks turn out to be equivalent, which generalises [36] to the non-trivial coefficient case.

### Theorem 1.1

(Theorem 2.19) The moduli stack $^\textrm{cl}\mathcal {X}$ of Laurent *F*-crystals on , i.e. the functor sending each *p*-nilpotent ring *R* to the groupoid  of Laurent *F*-crystals with coefficient in *R*, is equivalent to the Emerton–Gee stack $\mathcal {X}_\textrm{EG}$. Here the sheaf  is defined by  for any .

The basic idea of proving this theorem is to use the absolute perfect prismatic site as a bridge. When the coefficient rings are finite type $\mathbb {Z}_p$-algebras, there will be no difference between Laurent *F*-crystals on  and those on . In this case, both of them can be proved to be the same as étale $(\varphi ,\Gamma )$-modules. To extend the equivalence from finite type algebras to arbitrary algebras, the limit-preserving property of the moduli stack of étale $\varphi$-modules will be of great importance to us. We remark that it is essential to use the Breuil–Kisin prism, which is related to the Kummer extension instead of the cyclotomic extension. This is because the cyclotomic prism is only well-defined in the unramified case. So in order to deal with the ramified case, the Breuil–Kisin prism is necessary.

Now we turn to our initial question, i.e. construct a good derived extension of the Emerton–Gee stack. Naturally, one needs to figure out how to define derived étale $(\varphi ,\Gamma )$-modules. The subtlety lies in the continuous derived $\Gamma$-action. Note that the prismatic site or more precisely, the perfect prismatic site (or *v*-site) provides an intrinsic way to deal with continuous $\Gamma$-action. This suggests we stay in the realm of prismatic site to define derived stack of Laurent *F*-crystals and regard it as the derived Emerton–Gee stack in light of Theorem 1.1.

Allowing *R* to be any *p*-nilpotent animiated ring in Theorem 1.1, we get a derived stack $\mathcal {X}$ of Laurent *F*-crystals. Here comes the question: is $\mathcal {X}$ classical? Our second main result provides an affirmative answer to it.

### Theorem 1.2

(Theorem 3.3) The derived stack $\mathcal {X}$ of Laurent *F*-crystals is classical up to nilcompletion, i.e. when restricted to truncated animated rings, the derived stack $\mathcal {X}$ is equivalent to $({\textrm{Lan}}\mathcal {X}_\textrm{EG})^{\#}$, the (étale) sheafification of the left Kan extension of the Emerton–Gee stack along the inclusion from classical commutative rings to animated rings.

### Remark 1.3

The nilcompletion appearing in the above theorem is not essential as we mostly care about nilcomplete stacks in practice. Taking the nilcompletion is essentially due to that the left Kan extension of a prestack might not be nilcomplete in general (see Remark 3.7).

In fact, Theorem 1.2 is infinitesimally guaranteed by [2, Theorem 1.1], which states that the framed local Galois deformation ring is a local complete intersection ring. By [18, Lemma 7.5], this means the deformation functor of local Galois representations is classical. That being said, to extend this to the whole derived stack instead of just at the infinitesimal level is not as straightforward as the $l\ne p$ case. Namely, the Emerton–Gee stack is only a Noetherian formal algebraic stack instead of an algebraic stack, and the derived stack of Laurent *F*-crystals is only a derived stack abstractly defined, which is not a derived algebraic stack, either. This will cause essential troubles in proving the classicality.

Our basic tool is a global derived deformation theory, developed in [16] (see Appendix I for some subtleties in our context). The philosophy behind the global deformation theory is that the Postnikov tower of any animated ring is actually a successive extension of square-zero extensions, which is determined by the cotangent complex. In some good cases, comparing two derived stacks will be reduced to comparing their pro-cotangent complexes. This is roughly how we finally prove Theorem 1.2. More precisely, we have the following criterion.

### Proposition 1.4

[16, Chapter 1, Proposition 8.3.2] Let $\mathcal {F}_1\rightarrow \mathcal {F}_2$ be a map of derived prestacks admitting a deformation theory. Suppose there exists a commutative diagram,where $g_1,g_2$ are nilpotent embeddings, and $^\textrm{cl}\mathcal {F}_{0}$ is a classical prestack. Suppose also that for any discrete commutative ring $R\in {Nilp}_{\mathbb {Z}_p}$, and a map $x_0\in {^\textrm{cl}\mathcal {F}_{0}}(R)$ and $x_i=g_i\circ x_0$ where $i=1,2$, the induced map

$$ T^*_{x_2}(\mathcal {F}_2)\rightarrow T^*_{x_1}(\mathcal {F}_1) $$

is an isomorphism. Then *f* is an isomorphism.

Our first task then is to show both $\mathcal {X}$ and $({\textrm{Lan}}\mathcal {X}_\textrm{EG})^{\#}$ admit a deformation theory (see Definition 3.22), which reduces the comparison of $\mathcal {X}$ and $({\textrm{Lan}}\mathcal {X}_\textrm{EG})^{\#}$ to the comparison of their pro-cotangent complexes at classical points by Theorem 1.1 and Proposition 3.30. We can further reduce the comparison to classical points of finite type by proving both $\mathcal {X}$ and $({\textrm{Lan}}\mathcal {X}_\textrm{EG})^{\#}$ are locally almost of finite type (Lemma 3.31 and 3.33). However, pro-complexes are known to be technically difficult to deal with. For example, they do not satisfy the faithfully flat descent in general and one can not apply derived Nakayama lemma to them. This seems to make it impossible to reduce the comparison of pro-cotangent complexes at the points valued in finite type algebras to those valued in finite fields, which. Fortunately, we are able to prove the following result.

### Proposition 1.5

(Proposition 3.39 and 3.41) For any point $e:{\textrm{Spec}}(R)\rightarrow \mathcal {X}$ (resp. $e:{\textrm{Spec}}(R)\rightarrow ({\textrm{Lan}}\mathcal {X}_\textrm{EG})^{\#}$) with *R* being a classical finite type algebra over $\mathbb {Z}/p^a$ for some positive integer *a*, the pro-cotangent complex $T^*_e(\mathcal {X})$ (resp. $T^*_e(({\textrm{Lan}}\mathcal {X}_\textrm{EG})^{\#})$) is indeed a complex of *R*-modules.

In particular, the cotangent complex $T^*_e(\mathcal {X})$ is the shifted Herr complex associated to the adjoint étale $(\varphi ,\Gamma )$-module corresponding to the point *e*. This justifies the obstruction theory considered in [12].

Now we are able to reduce the comparison to classical points valued in finite fields. The new difficulty is that we have to deal with two different left Kan extensions: one is the left Kan extension from discrete Artinian local rings to Artinian local animated rings, the other is the left Kan extension from discrete commutative rings to animated rings. The former one is concerned with [18, Lemma 7.5]. The latter is what we care about. In order to apply [2, Theorem 1.1], we have to introduce the work of [38] on derived representations with general coefficients as a bridge. In the meantime, it is necessary to clarify the relation between derived representations and derived Laurent *F*-crystals. The whole proof of the classicalty then culminates in the technical Proposition 3.53.

### Remark 1.6

We will also give an ad-hoc definition of derived étale $(\varphi ,\Gamma )$-modules in the unramified case in Subsection 3.5, which might be easier to work with than Laurent *F*-crystals in practice.

### Remark 1.7

It seems possible to generalise the main results to the case of perfect complexes, i.e. the derived stack of Laurent *F*-crystals of perfect complexes is classical when restricted to truncated animated rings. But we do not pursue this generality here.

In some sense, Theorem 1.2 completes the picture that the moduli stacks of local Langlands parameters are all classical in the ${\textrm{GL}}_d$-case. To further study the case of general groups, a common strategy is to use the Tannakian formalism. Note that we do not know whether Theorem 1.2 will hold true for any group *G* (although we expect it to be the case if *G* is a reductive group). In the cases where the local Galois deformation ring is a local complete intersection, one still needs to use the nice geometric properties of the *G*-version of the Emerton–Gee stack to prove Theorem 1.2. We plan to investigate the derived Emerton–Gee stack for general groups in future work. Note that there are already some works in this direction, e.g. [23, 24].

### Convention

1. All the étale $\varphi$-modules and étale $(\varphi ,\Gamma )$-modules are projective of rank *d* so we will omit *d* in the notations.
2. For any $\infty$-category $\mathcal {C}$, we use $\mathcal {C}^{\simeq }$ to denote its core, i.e. its largest Kan subcomplex, and use $\textrm{Ho}(\mathcal {C})$ to denote its homotopy category.
3. For any simplicial (resp. cosimplicial) diagram, we will omit degeneracy (resp. codegeneracy) maps for simplicity.

## $^\textrm{cl}\mathcal {X}\simeq \mathcal {X}_\textrm{EG}$

Let *K* be a finite extension of $\mathbb {Q}_p$ with perfect residue field *k* and ring of integers $\mathcal {O}_K$. As we have mentioned in the introduction, Bhatt–Scholze and Wu have proved that there is an equivalence between the category of Laurent *F*-crystals on the absolute prismatic site  and the category of étale $(\varphi ,\Gamma )$-modules. There are two directions to generalise this result. One is to consider the geometric family, i.e. consider more general schemes instead of just $\mathcal {O}_K$. This has already been done in [5] and part of it has also been done in [32].

The other direction is to consider the arithmetic family. This is what we will do in this paper. Note that the arithmetic families of étale $(\varphi ,\Gamma )$-modules is just the Emerton–Gee stack $\mathcal {X}_\textrm{EG}$ in [12]. We mainly want to extend the above equivalence to more general coefficients. More precisely, we will construct the derived stack $\mathcal {X}$ of Laurent *F*-crystals and compare its underlying classical stack $^\textrm{cl}\mathcal {X}$ to $\mathcal {X}_\textrm{EG}$.

### The derived stack $\mathcal {X}$

Let $\text {Nilp}_{\mathbb {Z}_p}$ denote the category of *p*-nilpotent commutative rings, i.e. algebras over $\mathbb {Z}/p^a$ for some positive integer *a*, and ${\textbf {Nilp}}_{\mathbb {Z}_p}$ denote *p*-nilpotent animated rings, i.e. the animation of $\text {Nilp}_{\mathbb {Z}_p}$. Let ${\textbf {Ani}}$ denote the $\infty$-category of anima (i.e. $\infty$-category of $\infty$-groupoids). For more details about animation and anima, we refer to [8, Chapter 5].

We first recall the definitions of prestack and derived prestack.

#### Definition 2.1

(Prestack/stack) By a (classical) prestack, we mean a functor $\mathcal {F}: \text {Nilp}_{\mathbb {Z}_p}\rightarrow {\textbf {Ani}}$. If $\mathcal {F}$ satisfies the fppf descent, we call it a (classical) stack. Let $^\textrm{cl}{\textrm{PreStk}}$ denote the $\infty$-category of prestacks.

#### Remark 2.2

The (pre)stack here is also called higher (pre)stack in the literatures.

#### Definition 2.3

(Derived prestack/stack) By a derived prestack, we mean a functor $\mathcal {F}: {\textbf {Nilp}}_{\mathbb {Z}_p}\rightarrow {\textbf {Ani}}$. If *F* satisfies the fppf descent, we call it a derived stack. We use ${\textrm{PreStk}}$ to denote the $\infty$-category of derived prestacks. Let ${\textbf {Nilp}}_{\mathbb {Z}_p}^{\le n}$ denote the $\infty$-category of *n*-truncated animated rings, i.e. animated ring *R* with $\pi _i(R)=0$ for all $i>n$. We can define the $\infty$-category ${\textrm{PreStk}}^{\le n}$ of *n*-truncated prestacks, i.e. functors $\mathcal {F}:{\textbf {Nilp}}_{\mathbb {Z}_p}^{\le n}\rightarrow {\textbf {Ani}}$. In particular, ${\textrm{PreStk}}^{\le 0}={^\textrm{cl}{\textrm{PreStk}}}$.

For any derived prestack $\mathcal {F}$, one can get its underlying classical prestack $^\textrm{cl}\mathcal {F}$ by precomposing the natural inclusion $\text {Nilp}_{\mathbb {Z}_p}\rightarrow {\textbf {Nilp}}_{\mathbb {Z}_p}$.

Now for any animated ring $R\in {\textbf {Nilp}}_{\mathbb {Z}_p}$, we can define an $\infty$-presheaf  on the absolute prismatic site  (cf. [4, Remark 4.7], [5, Definition 2.3]) byThe $\infty$-presheaf  is actually an $\infty$-sheaf by the following theorem due to Drinfeld and Mathew.

#### Theorem 2.4

(Drinfeld–Mathew, [31, Theorem 5.8]) Let *R* be a connective $E_{\infty }$-ring, and *I* be a finitely generated ideal of $\pi _0(R)$. Then the following functors defined on the $\infty$-category of connective $E_{\infty }$-*R*-algebras

1. $$S\mapsto D_\textrm{perf}(\textrm{Spec}(S^{\wedge }_I)-V(IS))$$;
2. $$S\mapsto {\textrm{Vect}}(\textrm{Spec}(S^{\wedge }_I)-V(IS))$$

are sheaves for the *I*-completely flat topology.

To see  is a sheaf, let $(A,I)\rightarrow (B,I)$ be a (*p*, *I*)-completely faithfully flat map. Then we need to show

$$ (A\otimes ^{\mathbb {L}}_{\mathbb {Z}_p}R)^{\wedge }_{I}[\frac{1}{I}]\simeq \varprojlim _n(B^n\otimes ^{\mathbb {L}}_{\mathbb {Z}_p}R)^{\wedge }_{I}[\frac{1}{I}] $$

where $B^n$ is the $(n+1)$-fold (*p*, *I*)-completed tensor product of *B* over *A*. It suffices to prove the above equivalence by regarding all the terms as anima. Note that the mapping space $\textrm{Map}((B^n\otimes ^{\mathbb {L}}_{\mathbb {Z}_p}R)^{\wedge }_{I}[\frac{1}{I}],(B^n\otimes ^{\mathbb {L}}_{\mathbb {Z}_p}R)^{\wedge }_{I}[\frac{1}{I}])$ in $\mathcal {D}((B^n\otimes ^{\mathbb {L}}_{\mathbb {Z}_p}R)^{\wedge }_{I}[\frac{1}{I}])$, the derived $\infty$-category of $(B^n\otimes ^{\mathbb {L}}_{\mathbb {Z}_p}R)^{\wedge }_{I}[\frac{1}{I}]$-modules, is equivalent to $(B^n\otimes ^{\mathbb {L}}_{\mathbb {Z}_p}R)^{\wedge }_{I}[\frac{1}{I}]$ itself. And by the above theorem of Drinfeld–Mathew, we have

$$ \textrm{Map}\big ((A\otimes ^{\mathbb {L}}_{\mathbb {Z}_p}R)^{\wedge }_{I}\big [\frac{1}{I}\big ],(A\otimes ^{\mathbb {L}}_{\mathbb {Z}_p}R)^{\wedge }_{I}\big [\frac{1}{I}\big ]\big )\simeq \varprojlim _n\textrm{Map}\big ((B^n\otimes ^{\mathbb {L}}_{\mathbb {Z}_p}R)^{\wedge }_{I}\big [\frac{1}{I}\big ],(B^n\otimes ^{\mathbb {L}}_{\mathbb {Z}_p}R)^{\wedge }_{I}\big [\frac{1}{I}\big ]\big ) $$

which then gives the desired equivalence.

Let  denote the $\infty$-category of vector bundles,^1^ over . Then we have the following description by the *I*-completely faithfully flat descent.

#### Proposition 2.5

([5, Proposition 2.8]) There is an equivalence of $\infty$-categories

Note that as *R* is *p*-nilpotent, the Frobenius on the $\delta$-ring *A* induces a Frobenius map $\varphi$ on . Then we can define Laurent *F*-crystals with coefficients in *R* on  and the corresponding derived prestack of Laurent *F*-crystals.

Let us start with some discussions about derived étale $\varphi$-modules.

#### Definition 2.6

(Derived étale $\varphi$-modules) Let *A* be an animated ring equipped with an endomorphism $\varphi :A\rightarrow A$. Let ${\textrm{Vect}}(A)$ be the $\infty$-category of finite projective *A*-modules (cf. [25, Proposition 2.5.1 and 2.5.3, Theorem 2.5.2]) and $\textrm{Isom}({\textrm{Vect}}(A))$ be the full subcategory of $\textrm{Fun}(\Delta ^1,{\textrm{Vect}}(A))$ spanned by the isomorphisms. Then we define the $\infty$-category ${\textrm{Vect}}(A)^{\varphi =1}$ of étale $\varphi$-modules over *A* to be the (homotopy)^2^ pullbackwhere the functor $\varphi ^*$ is the base change functor $(-)\otimes _{A,\varphi }A$ and $ev_0$ (resp. $ev_1$) is the functor sending an isomorphism to the origin (resp. target). By construction, ${\textrm{Vect}}(A)^{\varphi =1}$ is the $\infty$-category of pairs $(M,\varphi _M)$ where *M* is a finite projective *A*-module and $\varphi _M:\varphi ^*M\rightarrow M$ is an isomorphism of *A*-modules. By [29, Proposition 7.6.5.18], ${\textrm{Vect}}(A)^{\varphi =1}$ is also equivalent to the homotopy pullbackwhich by [29, Proposition 7.6.5.20] is equivalent to the homotopy equalizer ofsimilar to the classical case.

#### Construction 2.7

(Dual of étale $\varphi$-module) For any $(M,\varphi _M)\in {\textrm{Vect}}(A)^{\varphi =1}$, we can construct its dual $(M^{\vee },\varphi _{M^{\vee }})$ as follows: $M^{\vee }:={\textrm{Hom}}_{\mathcal {D}(A)}(M,A)$ and $\varphi _{M^{\vee }}$ is the homotopy inverse of the natural map

$$ {\textrm{Hom}}_{\mathcal {D}(A)}(M,A)\xrightarrow {\simeq } {\textrm{Hom}}_{\mathcal {D}(A)}(\varphi ^*M,A)\simeq \varphi ^*{\textrm{Hom}}_{\mathcal {D}(A)}(M,A)$$

where $\mathcal {D}(A)$ is the (stable) $\infty$-category of *A*-modules and the second isomorphism follows from [25, Corollary 2.5.6].

#### Definition 2.8

(Frobenius invariants) For any $(M,\varphi _M)\in {\textrm{Vect}}(A)^{\varphi =1}$, by abuse of notation, we also write $\varphi _M:M\rightarrow M$ as the composite $M=M\otimes _{A,id}A\rightarrow \varphi ^*M\xrightarrow {\varphi _M}M$ and let $M^{\varphi _M=1}$ denote the equalizer ofin $\mathcal {D}(\mathbb {Z})$.

We have the following lemma concerning the mapping space between derived étale $\varphi$-modules.

#### Lemma 2.9

For any two objects $(M,\varphi _M)$ and $(N,\varphi _N)$ in ${\textrm{Vect}}(A)^{\varphi =1}$, we have

$$\begin{aligned} \textrm{Map}((M,\varphi _M),(N,\varphi _N))\simeq \tau _{\ge 0}(M^{\vee }\otimes N)^{\varphi _{M^{\vee }}\otimes \varphi _N=1}. \end{aligned}$$

#### Proof

By the description of ${\textrm{Vect}}(A)^{\varphi =1}$ as the homotopy equalizer, we get an equalizer diagramNote that $\textrm{Map}(M,N)\simeq \tau _{\ge 0}{\textrm{Hom}}(M,N)\simeq {\textrm{Hom}}(M,N)\simeq M^{\vee }\otimes N$.^3^ Then the map $\varphi ^*:\textrm{Map}(M,N)\rightarrow \textrm{Map}(M,N)$ defined as

$$ \textrm{Map}(M,N)\xrightarrow {f} \textrm{Map}(\varphi ^*M,\varphi ^*N)\xrightarrow {g} \textrm{Map}(M,N), $$

where *f* is induced by the pullback along $A\xrightarrow {\varphi }A$ and *g* is induced by the equivalences $M\xrightarrow {\varphi ^{-1}_M} \varphi ^*M$ and $\varphi ^*N\xrightarrow {\varphi _N} N$, is induced by

$$ M^{\vee }\otimes N\rightarrow (\varphi ^*M^{\vee })\otimes (\varphi ^*N)\simeq \varphi ^*(M^{\vee }\otimes N)\rightarrow M^{\vee }\otimes N. $$

Now this lemma follows from Definition 2.8. $\square$

Now we are ready to define Laurent *F*-crystals and the corresponding derived prestack.

#### Definition 2.10

(Laurent *F*-crystal) For any animated ring $R\in {\textbf {Nilp}}_{\mathbb {Z}_p}$, we define the $\infty$-category of Laurent *F*-crystals aswhere ${\textrm{Vect}}((A\otimes ^{\mathbb {L}}_{\mathbb {Z}_p}R)^{\wedge }_{I}[\frac{1}{I}])^{\varphi =1}$ denote the $\infty$-category of étale $\varphi$-modules over $(A\otimes ^{\mathbb {L}}_{\mathbb {Z}_p}R)^{\wedge }_{I}[\frac{1}{I}]$.

By Proposition 2.5, a Laurent *F*-crystal is simply a pair $(\mathbb {M},\varphi _{\mathbb {M}})$ where $\mathbb {M}$ is a vector bundle over  and $\varphi _{\mathbb {M}}:\varphi ^*\mathbb {M}\rightarrow \mathbb {M}$ is an isomorphism of -modules.

Let  (resp. ${\textrm{Vect}}((A\otimes ^{\mathbb {L}}_{\mathbb {Z}_p}R)^{\wedge }_{I}[\frac{1}{I}])^{\varphi =1,\simeq }$) denote the core of  (resp. ${\textrm{Vect}}((A\otimes ^{\mathbb {L}}_{\mathbb {Z}_p}R)^{\wedge }_{I}[\frac{1}{I}])^{\varphi =1}$, i.e. the largest Kan complex contained. As taking core commutes with limit by [30, Tag 01D8], we then get

#### Definition 2.11

The derived prestack $\mathcal {X}_{K}$ of Laurent *F*-crystals is defined asfor any $R\in {\textbf {Nilp}}_{\mathbb {Z}_p}$. As *K* is fixed throughout this paper, we simply write $\mathcal {X}$ instead of $\mathcal {X}_{K}$.

Again, by Theorem 2.4 due to Drinfeld and Mathew, we see that $\mathcal {X}$ is in fact a derived stack. Before proving this, we recall the definition of the Breuil–Kisin prism.

#### Definition 2.12

(Breuil–Kisin prism) Let $\pi$ be a fixed uniformiser of $\mathcal {O}_K$ and *E* is the Eisenstein polynomial of $\pi$. Let $\{\pi _{n}\}_n$ be a compatible system of *p*-power roots of $\pi$. We call  the Breuil–Kisin prism, where the Frobenius $\varphi _{\mathfrak {S}}$ is the natural one on *W*(*k*) and sends *u* to $u^p$, and its perfection $(A_{\infty }:=W(\mathcal {O}^{\flat }_{{\hat{K}}_{\infty }}),(E))$ the perfect Breuil–Kisin prism, where ${\hat{K}}_{\infty }$ is the *p*-adic completion of $\bigcup _n K(\pi _n)$.

#### Proposition 2.13

The derived prestack $\mathcal {X}$ is a derived stack for the fppf topology.

#### Proof

Let $R_1\rightarrow R_2$ be a faithfully flat map in ${\textbf {Nilp}}_{\mathbb {Z}_p}$. Then we need to provewhere $\{R_2^i\}_{i\ge 0}$ is the Čech nerve associated with $R_1\rightarrow R_2$.

Note that $(\mathfrak {S},(E))$ is a final cover of the topos  by [15, Lemma 2.2]. So we have2.1for any $R\in {\textbf {Nilp}}_{\mathbb {Z}_p}$, where $(\mathfrak {S}^{\bullet },(E))$ denotes the Čech nerve associated with $(\mathfrak {S},(E))$ and we simply write $\mathfrak {S}_R^n=(\mathfrak {S}^n\otimes ^{\mathbb {L}}_{\mathbb {Z}_p}R)^{\wedge }_{E}$. For any $(\mathfrak {S}^n,(E))$, we see that $\mathfrak {S}^n\otimes ^{\mathbb {L}}_{\mathbb {Z}_p}R_1\rightarrow \mathfrak {S}^n\otimes ^{\mathbb {L}}_{\mathbb {Z}_p}R_2$ is (*E*-completely) faithfully flat. So we can apply Theorem 2.4 and get

$$ {\textrm{Vect}}(\mathfrak {S}^n_{R_1}[\frac{1}{E}])^{\varphi =1,\simeq }\xrightarrow {\simeq }\varprojlim _i{\textrm{Vect}}(\mathfrak {S}^n_{R^i_2}[\frac{1}{E}])^{\varphi =1,\simeq } $$

Now the proposition follows from the fact that limit commutes with limit. $\square$

We conclude this subsection with a key lemma which will be used frequently.

#### Lemma 2.14

[21, Proposition A.1] Let $\mathcal {F}:\Delta \rightarrow {\textbf {Ani}}$ be a cosimplicial anima such that all $\mathcal {F}([i])$’s are *n*-truncated. Then there is an equivalence

$$ \varprojlim _{\Delta }\mathcal {F}\xrightarrow {\simeq } \varprojlim _{\Delta _{\le n+1}}\mathcal {F}|_{\Delta _{\le n+1}}, $$

where $\Delta _{\le n+1}$ denotes the full subcategory of $\Delta$ with objects $[0],[1],[2],\cdots ,[n+1]$.

#### Proof

Note that $\mathcal {F}:\Delta \rightarrow {\textbf {Ani}}$ actually factors through $\mathcal {F}:\Delta \rightarrow {\textbf {Ani}}^{\le n}\rightarrow {\textbf {Ani}}$, where ${\textbf {Ani}}^{\le n}$ is spanned by all the *n*-truncated animated sets. $\square$

### The underlying classical stack $^\textrm{cl}\mathcal {X}$

In this subsection, we will study the underlying classical stack $^\textrm{cl}\mathcal {X}$ of $\mathcal {X}$ and compare it with the Emerton–Gee stack $\mathcal {X}_\textrm{EG}$ constructed in [12].

Let’s first briefly recall the construction of the Emerton–Gee stack and some of its basic properties. We fix a compatible system $\{\zeta _{p^n}\}_n$ of primitive *p*-power roots of unity in $\bar{K}$ and write $K_{{\textrm{cyc}}}:=\bigcup _n K(\zeta _{p^n})$. Let $k_{\infty }$ denote the residue field of $K(\zeta _{p^{\infty }})$. Write $A_K^+=W(k_{\infty })[[T_K]]$, where $T_K$ is a chosen lift in $A_K$ of the uniformiser of the field of norms of $K(\zeta _{p^{\infty }})$ and $A_K=A_K^+[\frac{1}{T_K}]^{\wedge }_p$. There is a $\varphi$-action on $A_K$ as well as a compatible $\Gamma :={\textrm{Gal}}(K(\zeta _{p^{\infty }})/K)$-action. The subring $A_K^+$ is not necessarily stable under the $\varphi$-action. If *K* is absolutely unramified, then there is a $\varphi$-action such that $A_K^+$ is $\varphi$-stable.

#### Definition 2.15

(Étale $(\varphi ,\Gamma )$-modules) Let *R* be a *p*-nilpotent ring. We say *M* is an étale $(\varphi ,\Gamma )$-module with *R*-coefficient if *M* is a finite projective module over $A_{K,R}:=(A_K^+\otimes _{\mathbb {Z}_p}R)^{\wedge }_{T_K}[\frac{1}{T_K}]$ equipped with compatible semilinear Frobenius action $\varphi _M:\varphi ^*M\xrightarrow {\simeq }M$ and continuous $\Gamma$-action. Let $\textrm{Mod}^{\varphi ,\Gamma }(A_{K,R})$ denote the category of étale $(\varphi ,\Gamma )$-modules with *R*-coefficient and $\textrm{Mod}^{\varphi ,\Gamma }(A_{K,R})^{\simeq }$ denote its underlying groupoid.

#### Theorem 2.16

[12] Let $\mathcal {X}_\textrm{EG}$ be the classical prestack defined as $\mathcal {X}_\textrm{EG}(R):=\textrm{Mod}^{\varphi ,\Gamma }(A_{K,R})^{\simeq }$. Then $\mathcal {X}_\textrm{EG}$ is a Noetherian formal algebraic stack. In particular, it is also an ind-algebraic stack locally of finite type.

Now we turn to the derived stack $\mathcal {X}$. Note that when *R* is discrete, the sheaf  is not necessarily a discrete $\infty$-sheaf. But the $\infty$-groupoid  is indeed an 1-groupoid. In fact, in Eq. 2.1, the right hand side is an inverse limit of 1-groupoids as each $\mathfrak {S}^n_R[\frac{1}{E}]$ is discrete. So $^\textrm{cl}\mathcal {X}$ is an fppf stack valued in 1-groupoids in the classical sense.

In order to compare $^\textrm{cl}\mathcal {X}$ and $\mathcal {X}_\textrm{EG}$, we need to construct a map between them in the first place. To achieve this, we recall some important prisms in .

#### Definition 2.17

(Cyclotomic prism) Let ${\hat{K}}_{{\textrm{cyc}}}^{\flat }$ be the tilt of the *p*-adic completion of $K(\zeta _{p^{\infty }})$ and define $\epsilon :=(1,\zeta _p,\zeta _{p^2},\cdots )\in {\hat{K}}_{{\textrm{cyc}}}^{\flat }$. Write $q=[\epsilon ]$ and $[p]_q:=\frac{q^p-1}{q-1}$. When *K* is absolutely unramified, by [36, Proposition 2.19], $(\bar{A}_K^+,[p]_q)$^4^ is a prism in . We will call it the cyclotomic prism and is equipped with compatible $\varphi$-action and $\Gamma$-action. By [36, Lemma 2.17], its perfection is $(A_{{\textrm{cyc}}}:=W(\mathcal {O}^{\flat }_{{\hat{K}}_{{\textrm{cyc}}}}),(\xi ))$, which we call the perfect cyclotomic prism. When *K* is ramified, there is no well-defined cyclotomic prism but the perfect cyclotomic prism $(A_{{\textrm{cyc}}}:=W(\mathcal {O}^{\flat }_{{\hat{K}}_{{\textrm{cyc}}}}),(\xi ))$ still makes sense.

#### Definition 2.18

(Étale $(\varphi ,\Gamma )$-modules over the perfect cyclotomic prism) Let *R* be a *p*-nilpotent ring. We say *M* is an étale $(\varphi ,\Gamma )$-module over the perfect cyclotomic prism with *R*-coefficient if *M* is a finite projective module over $A_{{\textrm{cyc}},R}[\frac{1}{\xi }]:=(A_{{\textrm{cyc}}}\otimes _{\mathbb {Z}_p}R)^{\wedge }_{\xi }[\frac{1}{\xi }]$ equipped with compatible semilinear Frobenius action $\varphi _M:\varphi ^*M\xrightarrow {\simeq }M$ and continuous $\Gamma$-action. Let $\textrm{Mod}^{\varphi ,\Gamma }(A_{{\textrm{cyc}},R}[\frac{1}{\xi }])$ denote the category of étale $(\varphi ,\Gamma )$-modules with *R*-coefficient and $\textrm{Mod}^{\varphi ,\Gamma }(A_{{\textrm{cyc}},R}[\frac{1}{\xi }])^{\simeq }$ denote its underlying groupoid.

Note that both the cyclotomic prism and the Breuil–Kisin prism are covers of the final object of the topos . Now we state the main result of this section.

#### Theorem 2.19

There is a natural equivalence between $^\textrm{cl}\mathcal {X}$ and $\mathcal {X}_\textrm{EG}$.

To prove this theorem, we will proceed in two steps. The first one is to compare étale $(\varphi ,\Gamma )$-modules with coefficients to Laurent *F*-crystals with coefficients on the absolute perfect prismatic site. There is a natural restriction map from  to , which will then link  to $\textrm{Mod}^{\varphi ,\Gamma }(A_{K,R})$. But this step can only provide comparison when the coefficient rings are finite type $\mathbb {Z}_p$-algebras. This is closely related to the “non-Noetherian" nature of the perfect absolute prismatic site . To finally prove Theorem 2.19, we will need the second step: prove limit-preserving property of $^\textrm{cl}\mathcal {X}$. In a more geometric language, we will prove $^\textrm{cl}\mathcal {X}$ is locally of finite presentation.

We begin with how Laurent *F*-crystals are related to “non-Noetherian" étale $(\varphi ,\Gamma )$-modules.

#### Proposition 2.20

For any $\mathbb {Z}/p^a$-algebra *R*, there is an equivalence of categories

#### Proof

Since $(A_{{\textrm{cyc}}},(\xi ))$ is a cover of the final object of the topos , we see that any Laurent *F*-crystal corresponds to a stratification $(\mathcal {M},\epsilon )$ with respect to the Čech nerve $(A_{{\textrm{cyc}}}^{\bullet },(\xi ))$ of $(A_{{\textrm{cyc}}},(\xi ))$ in , where $\mathcal {M}$ is a finite projective $A_{{\textrm{cyc}},R}[\frac{1}{\xi }]$-module and $\epsilon$ is an isomorphism of $A^1_{{\textrm{cyc}},R}[\frac{1}{\xi }]$-modules:

$$ \mathcal {M}\otimes _{A_{{\textrm{cyc}},R}[\frac{1}{\xi }],p_0}A^1_{{\textrm{cyc}},R}[\frac{1}{\xi }]\cong \mathcal {M}\otimes _{A_{{\textrm{cyc}},R}[\frac{1}{\xi }],p_1}A^1_{{\textrm{cyc}},R}[\frac{1}{\xi }] $$

which satisfies the cocycle condition. Now this theorem follows from Lemma 2.22. $\square$

#### Remark 2.21

One can also replace $\textrm{Mod}^{\varphi ,\Gamma }(A_{{\textrm{cyc}},R}[\frac{1}{\xi }])$ by the category $\textrm{Mod}^{\varphi ,G_K} (A_{\inf ,R}[\frac{1}{\xi }])$ of étale $(\varphi ,G_K)$-modules over $A_{\inf ,R}[\frac{1}{\xi }]$ in Theorem 2.20.

#### Lemma 2.22

For any $\mathbb {Z}/p^a$-algebra *R*, there is an isomorphism of rings

$$ A^n_{{\textrm{cyc}},R}[\frac{1}{\xi }]\cong C(\Gamma ^n, A_{{\textrm{cyc}},R}[\frac{1}{\xi }]) $$

for all $$n\ge 0$$.

#### Proof

Consider $(C(\Gamma ^n,A_{{\textrm{cyc}}}),(\xi ))$ as a perfect prism in . Then there is a natural map

$$ (A_{{\textrm{cyc}}}^n,(\xi ))\rightarrow (C(\Gamma ^n,A_{{\textrm{cyc}}}),(\xi )) $$

induced by the maps of prisms $\gamma _i:(A_{{\textrm{cyc}}},(\xi ))\rightarrow (C(\Gamma ^n,A_{{\textrm{cyc}}}),(\xi ))$ defined as $(\gamma _i(x))(g)=(\textrm{pr}_i(g))(x)$, where $\textrm{pr}_i$ is the *i*-th projection map $\Gamma ^n\rightarrow \Gamma$ when $1\le i\le n$ and the map $\Gamma ^n\rightarrow *$ when $i=0$.

Note that by [36, Lemma 5.3], there is an isomorphism

$$ A^n_{{\textrm{cyc}}}[\frac{1}{\xi }]/p^m\cong C(\Gamma ^n,A_{{\textrm{cyc}}}[\frac{1}{\xi }]/p^m) $$

for any *m*. In particular the homotopy cofiber *M* of the map $A^n_{{\textrm{cyc}}}/p^m\rightarrow C(\Gamma ^n,A_{{\textrm{cyc}}})/p^m$ vanishes after inverting $\xi$. As both $A^n_{{\textrm{cyc}}}/p^m$ and $C(\Gamma ^n,A_{{\textrm{cyc}}})/p^m$ are derived $\xi$-complete, so is the homotopy cofiber *M*. Then by [1, Theorem 2.3], we see that *M* is killed by $\xi ^s$ for some *s*.

Now for any finite type $\mathbb {Z}/p^a$-algebra *R*, the map

$$ A^n_{{\textrm{cyc}}}/p^a\otimes _{\mathbb {Z}/p^a} R\rightarrow C(\Gamma ^n, A_{{\textrm{cyc}}}/p^a)\otimes _{\mathbb {Z}/p^a} R $$

becomes isomorphism after inverting $\xi$. We can consider the following exact sequence of derived $\xi$-complete $A_{{\textrm{cyc}}}$-modules

$$ A^n_{{\textrm{cyc}}}/p^a{\widehat{\otimes }}_{\mathbb {Z}/p^a}^{\mathbb {L}} R\rightarrow C(\Gamma ^n, A_{{\textrm{cyc}}}/p^a){\widehat{\otimes }}_{\mathbb {Z}/p^a}^{\mathbb {L}} R\rightarrow M{\widehat{\otimes }}_{\mathbb {Z}/p^a}^{\mathbb {L}}R, $$

where by abuse of notation ${\widehat{\otimes }}^{\mathbb {L}}$ means derived $\xi$-adic completion of the derived tensor product.

We claim that $M{\widehat{\otimes }}_{\mathbb {Z}/p^a}^{\mathbb {L}}R$ vanishes after inverting $\xi$. By [34, Tag 0CQE] and [34, Tag 0923], it is easy to see this is true if $M\otimes ^{\mathbb {L}}_{\mathbb {Z}/p^a}R$ is killed by some bounded power of $\xi$. To see the latter, first note that $H^0(M\otimes _{\mathbb {Z}/p^a}^{\mathbb {L}}R)=H^0(M)\otimes _{\mathbb {Z}/p^a}R$ is killed by $\xi ^s$. Note that $M\otimes ^{\mathbb {L}}_{\mathbb {Z}/p^a}R$ is concentrated in homological degree [0, 1] as $A^n_{{\textrm{cyc}}}/p^a$ is flat over $\mathbb {Z}/p^a$. To investigate $H^{-1}(M\otimes _{\mathbb {Z}/p^a}^{\mathbb {L}}R)$, we examine the homology Künneth spectral sequence

$$ E^2_{i,j}=\textrm{Tor}^{\mathbb {Z}/p^a}_i(H_j(M),R)\Longrightarrow H_{i+j}(M\otimes _{\mathbb {Z}/p^a}^{\mathbb {L}}R). $$

Note that both $E^2_{0,1}=H_1(M)\otimes _{\mathbb {Z}/p^a}R$ and $E^2_{1,0}=\textrm{Tor}^{\mathbb {Z}/p^a}_1(H_0(M),R)$ are killed by $\xi ^s$. So both $E^{\infty }_{0,1}$ and $E^{\infty }_{1,0}$ are killed by $\xi ^s$. This implies that $H^{-1}(M\otimes _{\mathbb {Z}/p^a}^{\mathbb {L}}R)=H_1(M\otimes _{\mathbb {Z}/p^a}^{\mathbb {L}}R)$ is killed by $\xi ^{2s}$. Hence $M\otimes _{\mathbb {Z}/p^a}^{\mathbb {L}}R$ is killed by $\xi ^{2s}$.

We finally get

$$ A^n_{{\textrm{cyc}},R}[\frac{1}{\xi }]\cong (C(\Gamma ^n,A_{{\textrm{cyc}}}/p^a){\widehat{\otimes }}_{\mathbb {Z}/p^a} R)[\frac{1}{\xi }]. $$

It remains to prove

$$ C(\Gamma ^n,A_{{\textrm{cyc}}}/p^a){\widehat{\otimes }}_{\mathbb {Z}/p^a} R\cong C(\Gamma ^n,A_{{\textrm{cyc}}}/p^a{\widehat{\otimes }}_{\mathbb {Z}/p^a} R) $$

where the completion means $\xi$-adic completion.

This follows from $A_{{\textrm{cyc}}}/p^a$ is $\xi$-adic complete. $\square$

Before moving on, we give a lemma that will be used frequently.

#### Lemma 2.23

Let $f:\mathcal {C}\rightarrow \mathcal {D}$ be a morphism between two cosimplicial $\infty$-categories. Suppose $f_i:\mathcal {C}_{[i]}\rightarrow \mathcal {D}_{[i]}$ is fully faithful for all [*i*]’s and $f_0:\mathcal {C}_{[0]}\rightarrow \mathcal {D}_{[0]}$ is an equivalence. Then $f:\textrm{Tot}(\mathcal {C})\rightarrow \textrm{Tot}(\mathcal {D})$ is an equivalence.

#### Proof

As all $f_i$’s are fully faithful, we know that *f* must be fully faithful. Moreover since $f_0$ is an equivalence and all $f_i$’s are fully faithful, we can construct a map from $\textrm{Tot}(\mathcal {D})$ to $\mathcal {C}$ using $f_0^{-1}$ which induces a map $g:\textrm{Tot}(\mathcal {D})\rightarrow \textrm{Tot}(\mathcal {C})$. By the full faithfulness of all $f_i$’s again, we see that $f\circ g\simeq id:\textrm{Tot}(\mathcal {D})\rightarrow \textrm{Tot}(\mathcal {D})$. In particular, this implies that *f* is essentially surjective. So we get *f* is an equivalence. $\square$

Now we need to compare Laurent *F*-crystals on the absolute prismatic site and those on the absolute perfect prismatic site.

#### Proposition 2.24

Let *R* be a finite type $\mathbb {Z}/p^a$-algebra. There is a natural equivalence of categories

In order to prove Proposition 2.24, we need the following lemma due to Bhatt–Scholze.

#### Lemma 2.25

[4, Lemma 9.2] Let *B* be a $\mathbb {Z}_p$-algebra with a Frobenius lift $\varphi$. Suppose there is an element $t\in B$ such that $\varphi (t)\in t^2B$. Let $\mathcal {D}(B[F])$ be the $\infty$-category of pairs $(M,\varphi )$ where $M\in \mathcal {D}(B)$ and $\varphi :M\rightarrow \varphi _*M$ is a map. Let $\mathcal {D}_\textrm{comp}(B[F])$ be the full subcategory of $\mathcal {D}(B[F])$ consisting of pairs $(M,\varphi )$ with *M* derived *t*-adic complete. Then the functors $\mathcal {D}_\textrm{comp}(B[F]) \rightarrow \mathcal {D}(\mathbb {Z}_p)$ given by $(M,\varphi )\mapsto M^{\varphi =1}$ and $(M,\varphi )\mapsto (M[\frac{1}{t}])^{\varphi =1}$ commute with colimits.

#### Proof

The proof is the same as that of [4, Lemma 9.2]. For readers’ convenience, we repeat it here. All colimits in this proof refer to the colimits in $\mathcal {D}(B)$.

Let $(M_i,\varphi _i)$ be a diagram in $\mathcal {D}_\textrm{comp}(B[F])$. Let $\widehat{\varinjlim M_i}$ be the derived *t*-adic completion of $\varinjlim M_i$. Note that the fiber *F* of the map $\varinjlim M_i\rightarrow \widehat{\varinjlim M_i}$ is uniquely *t*-divisible and thus identifies with the fiber of $\varinjlim M_i[\frac{1}{t}]\rightarrow \widehat{\varinjlim M_i}[\frac{1}{t}]$. Then the lemma can be reformulated as $F^{\varphi =1}=0$ and we just need to prove the statement for the functor $(M,\varphi )\mapsto M^{\varphi =1}$. Next consider the fiber ${\bar{N}}$ of the map $N\rightarrow N/t$ for any $N\in \mathcal {D}_\textrm{comp}(B[F])$. Note that ${\bar{N}}$ is derived *t*-complete. As $\varphi (t)\in t^2B$, ${\bar{N}}$ and *N*/*t* both have induced $\varphi$-actions. Moreover as the $\varphi$-action on ${\bar{N}}$ is topologically nilpotent with respect to the *t*-adic topology, we have ${\bar{N}}^{\varphi =1}=0$. This implies $N^{\varphi =1}\xrightarrow {\simeq }(N/t)^{\varphi =1}$. Since both functors in the composition $\mathcal {D}_\textrm{comp}(B[F])\xrightarrow {N\rightarrow N/t}\mathcal {D}(B[F])\xrightarrow {(-)^{\varphi =1}} \mathcal {D}(\mathbb {Z}_p)$ commute with colimits, the lemma follows. $\square$

#### Proof of Proposition 2.24

By using the Breuil–Kisin prism $(\mathfrak {S},(E))$ and its perfection $(A_{\infty },(E))$, we can write both categories as a 2-limit of categoriesandwhere $(\mathfrak {S}^{\bullet },(E))$ (resp. $(A_{\infty }^{\bullet },(E))$) is the Čech nerve associated with $(\mathfrak {S},(E))$ in  (resp. $(A_{\infty },(E))$ in ) and $\mathfrak {S}^{\bullet }_R:=(\mathfrak {S}^{\bullet }\otimes _{\mathbb {Z}_p}R)^{\wedge }_{E}$ (resp. $A_{\infty ,R}^{\bullet }:=(A_{\infty }\otimes _{\mathbb {Z}_p}R)^{\wedge }_{E}$). As *R* is *p*-nilpotent, we can replace all *E* by *u*.

By [12, Proposition 2.6.12], the natural functor $\textrm{Res}^0:{\textrm{Vect}}(\mathfrak {S}_{R}[\frac{1}{u}])^{\varphi =1}\rightarrow {\textrm{Vect}}(A_{\infty ,R}[\frac{1}{u}])^{\varphi =1}$ is an equivalence. By Lemma 2.25, the functor $\textrm{Res}^i:{\textrm{Vect}}(\mathfrak {S}^i_{R}[\frac{1}{u}])^{\varphi =1}\rightarrow {\textrm{Vect}}(A^i_{\infty ,R}[\frac{1}{u}])^{\varphi =1}$ is fully faithful for all *i*. In fact, we claim that $M^{\varphi =1}\cong (M\otimes _{\mathfrak {S}_R^i[\frac{1}{u}]}A_{\infty ,R}^i[\frac{1}{u}])^{\varphi =1}$ for any $M\in {\textrm{Vect}}(\mathfrak {S}^i_{R}[\frac{1}{u}])^{\varphi =1}$. Then the full faithfulness follows from Lemma 2.9. Combining the equivalence of $\textrm{Res}^0$ and the full faithfulness of $\textrm{Res}^i$ for all *i*, we can see the restriction functor  is indeed an equivalence by Lemma 2.23. Or an easy way to see this is that every stratification with respect to $A_{\infty ,R}^{\bullet }[\frac{1}{E}]$ can now be descended to a stratification with respect to $\mathfrak {S}_{R}^{\bullet }[\frac{1}{E}]$.

Now we prove the claim. By [11, Lemma 5.2.14], for any $(M,\varphi _M)\in {\textrm{Vect}} (\mathfrak {S}^i_{R}[\frac{1}{u}])^{\varphi =1}$, there exists $(N,\varphi _N)\in {\textrm{Vect}}(\mathfrak {S}^i_{R}[\frac{1}{u}])^{\varphi =1}$ such that $(M\oplus N, \varphi _M\oplus \varphi _N)\in {\textrm{Vect}}(\mathfrak {S}^i_{R}[\frac{1}{u}])^{\varphi =1}$ is a finite free étale $\varphi$-module. So without loss of generality, we may assume *M* is finite free. In this case, we can always find a lattice $(M_0,\varphi )$ over $\mathfrak {S}_R^i$ inside *M* by [11, Lemma 5.2.15].

Now we want to apply Lemma 2.25 to the pair $(\mathfrak {S},u)$ and $M_0$. Note that $\varphi (u)=u^p\in \mathfrak {S}$. So now what we need is to prove

$$ A_{\infty ,R}^{i}\simeq (\varinjlim _{\varphi }\mathfrak {S}^{i}_{R})^{\wedge }_{u,\mathrm derived} $$

where the completion on the right hand side is the derived *u*-adic completion.^5^ Note that $(A_{\infty }^i,(E))$ is the perfection of $(\mathfrak {S}^i,(E))$. By [4, Lemma 3.9], we see that

$$ A_{\infty }^i\simeq (\varinjlim _{\varphi }\mathfrak {S}^i)^{\wedge }_{(p,E)}\simeq (\varinjlim _{\varphi }\mathfrak {S}^i)^{\wedge }_{(p,E),\mathrm derived}. $$

Then by derived Nakayama lemma, we have

$$ A_{\infty }^i/p^a\simeq (\varinjlim _{\varphi }\mathfrak {S}^i/p^a)^{\wedge }_{E,\mathrm derived}\simeq (\varinjlim _{\varphi }\mathfrak {S}^i/p^a)^{\wedge }_{u,\mathrm derived} $$

which implies

$$\begin{aligned} A_{\infty ,R}^i&\simeq (A^i_{\infty }/p^a\otimes _{\mathbb {Z}_p/p^a}R)^{\wedge }_{u}\ \simeq (A^i_{\infty }/p^a\otimes ^{\mathbb {L}}_{\mathbb {Z}_p/p^a}R)^{\wedge }_{u,\mathrm derived}\\&\simeq ((\varinjlim _{\varphi }\mathfrak {S}^i/p^a)\otimes _{\mathbb {Z}_p/p^a}^{\mathbb {L}}R)^{\wedge }_{u,\mathrm derived}\simeq (\varinjlim _{\varphi }\mathfrak {S}^{i}_{R})^{\wedge }_{u,\mathrm derived}. \end{aligned}$$

To see the second isomorphism above, i.e. the derived *u*-adic completion of $A^i_{\infty }/p^a\otimes _{\mathbb {Z}/p^a}R$ is the same as the classical *u*-adic completion, it suffices to show $(A^i_{\infty }/p^a\otimes _{\mathbb {Z}/p^a}R)[u^{\infty }]$ is bounded. By [34, Tag 0912], we see that $\mathfrak {S}\rightarrow A_{\infty }^i$ is (faithfully) flat instead of just (*p*, *u*)-complete flat. So $(A^i_{\infty }/p^a\otimes _{\mathbb {Z}/p^a}R)[u^{\infty }]=(\mathfrak {S}/p^a\otimes _{\mathbb {Z}/p^a}R)[u^{\infty }]\otimes _{\mathfrak {S}}A_{\infty }^i$. Note that $\mathfrak {S}/p^a\otimes _{\mathbb {Z}/p^a}R$ is Noetherian. So we are done.

Note that $\varphi ^*M$ is equipped with a natural Frobenius map such that $\varphi _M:\varphi ^*M\rightarrow M$ induces an isomorphism of étale $\varphi$-modules. In particular, we have $\varphi _M:(\varphi ^*M)^{\varphi =1}\simeq M^{\varphi =1}$. On the other hand, the composite of the Frobenius equivariant maps $M\rightarrow \varphi ^*M$ and $\varphi _M:\varphi ^*M\rightarrow M$ is exactly the Frobenius semilinear map $\varphi _M:M\rightarrow M$. As $\varphi _M=id: M^{\varphi =1}\rightarrow M^{\varphi =1}$, we see that $M^{\varphi =1}\xrightarrow {\simeq }(\varphi ^*M)^{\varphi =1}$. The same argument then shows that the natural map $(\varphi ^n)^*M\rightarrow (\varphi ^{n+1})^*M$ induces $((\varphi ^n)^*M)^{\varphi =1}\simeq ((\varphi ^{n+1})^*M)^{\varphi =1}$. This implies $M^{\varphi =1}\simeq \varinjlim _n ((\varphi ^n)^*M)^{\varphi =1}$.

By Lemma 2.25, we finally get

$$\begin{aligned} M^{\varphi =1}&\simeq \varinjlim _n ((\varphi ^n)^*M)^{\varphi =1}\simeq (((\varinjlim _{\varphi }\mathfrak {S}_R^i)\otimes _{\mathfrak {S}_R^i}M_0)^{\wedge }_{u,\mathrm derived}[\frac{1}{u}])^{\varphi =1} \\&\simeq (A_{\infty ,R}^i\otimes _{\mathfrak {S}^i_R}M_0[\frac{1}{u}])^{\varphi =1}. \end{aligned}$$

#### Remark 2.26

In the unramified case, we may also use the cyclotomic prism $(\bar{A}_K^+,[p]_q)$ instead of the Breuil–Kisin prism in the proof of Proposition 2.24. In fact, let *a* be a positive integer, we just need to find a suitable element $t\in \bar{A}_K^+/p^a$ with $\varphi (t)\in t^2{\bar{A}}_K^+/p^a$ so that we can apply Lemma 2.25. As $\varphi ([p]_q)=[p]_q^p+p\delta ([p]_q)\in {\bar{A}}_K^+$, we have $\varphi ([p]_q^m)\equiv [p]_q^{pm} \mod p^a$ for some large enough integer *m*. So we can simply choose *t* to be the image of $[p]_q^m$ in ${\bar{A}}_K^+/p^a$.

Recall the following important proposition in [12].

#### Proposition 2.27

[12, Proposition 2.7.8] For any finite type $\mathbb {Z}/p^a$-algebra *R*, there is an equivalence of categories

$$ \textrm{Mod}^{\varphi ,\Gamma }(A_{{\textrm{cyc}},R}[\frac{1}{\xi }])\simeq \textrm{Mod}^{\varphi ,\Gamma }(A_{K,R}) $$

We then have the immediate corollary.

#### Corollary 2.28

For any finite type $\mathbb {Z}/p^a$-algebra *R*, there is an equivalence of categories

#### Proof

Just combine Proposition 2.20, Proposition 2.24 and Proposition 2.27. $\square$

In order to extend this equivalence to all $\mathbb {Z}/p^a$-algebras, we recall that the Emerton–Gee stack $\mathcal {X}_\textrm{EG}$ is limit preserving.

#### Lemma 2.29

[12, Lemma 3.2.19] The Emerton–Gee stack $\mathcal {X}_\textrm{EG}$ is limit preserving. In particular, $\mathcal {X}_\textrm{EG}(R)=\varinjlim _i\mathcal {X}_\textrm{EG}(R_i)$ where *R* is any $\mathbb {Z}/p^a$-algebra and $\{R_i\}$ is the filtered colimit system of all the finite type sub-$\mathbb {Z}/p^a$-algebras of *R*.

So to prove Theorem 2.19, we just need to prove the classical stack $\mathcal {X}$ of Laurent *F*-crystals is also limit preserving.

#### Lemma 2.30

For any $\mathbb {Z}/p^a$-algebra $R=\varinjlim _i R_i$ where $\{R_i\}$ is a filtered colimit system of finite type $\mathbb {Z}/p^a$-algebras, we have $^\textrm{cl}\mathcal {X}(R)=\varinjlim _i^\textrm{cl}\mathcal {X}(R_i)$.

#### Proof

By using the Breuil–Kisin prism $(\mathfrak {S},(E))$ as the cover of the final object of the topos , we can writefor any *i*. We can also take the core of each category as taking core commutes with limits.

As filtered colimit commutes with finite limits in 1-Groupoids, we then haveThere is a natural map $\varinjlim _i {^\textrm{cl}\mathcal {X}}(R_i)\rightarrow {^\textrm{cl}\mathcal {X}(R)}$ by base change. By [12, Corollary 3.1.5 and 3.2.6], the moduli stack of étale $\varphi$-modules is limit preserving, i.e. we have $\varinjlim _i({\textrm{Vect}}(\mathfrak {S}_{R_i}[\frac{1}{E}])^{\varphi =1,\simeq })\simeq {\textrm{Vect}}(\mathfrak {S}_R[\frac{1}{E}])^{\varphi =1,\simeq }$. By Lemma 2.9 and Lemma 2.25, we get the base change functor $\varinjlim _i{\textrm{Vect}}(\mathfrak {S}^j_{R_i}[\frac{1}{E}])^{\varphi =1}\rightarrow {\textrm{Vect}}(\mathfrak {S}_R^j[\frac{1}{E}])^{\varphi =1}$ is fully faithful for $j=1,2$. This means we finally have an equivalence of categories $^\textrm{cl}\mathcal {X}(R)=\varinjlim _i{^\textrm{cl}\mathcal {X}}(R_i)$ by Lemma 2.23. $\square$

#### Proof of Theorem 2.19

This follows from Corollary 2.28 and Lemma 2.30.

#### Remark 2.31

Perpendicular to the cyclotomic case, one can also try to construct a moduli stack of étale $(\varphi ,\tau )$-modules in the Kummer case. Then by using similar arguments, one can prove it is equivalent to the stack $^\textrm{cl}\mathcal {X}$ as least when restricted to the finite type $\mathbb {Z}_p$-algebras. We are not sure if the stack of étale $(\varphi ,\tau )$-modules is limit-preserving or not. But in practice, one can always modify it to be limit-preserving.

## $\mathcal {X}^\textrm{nil}\simeq ({\textrm{Lan}}\mathcal {X}_\textrm{EG})^{\#,\mathrm nil}$

In this section, we shift our focus to the derived stack $\mathcal {X}$ of Laurent *F*-crystals. As we have mentioned in the introduction, we hope the derived stack $\mathcal {X}$ is indeed “classical”, which roughly means it is determined by its underlying stack. This will then complete the picture that “derived stacks of local Langlands parameters are all classical”.

The possibility of the classicality of $\mathcal {X}$ is suggested by the following theorem.

### Theorem 3.1

[2, Theorem 1.1] Let $k_f$ be a finite field of characteristic *p*. Let ${\bar{\rho }}:G_K\rightarrow GL_d(k_f)$ be a residual representation. Then the framed local Galois deformation ring $R^{\square }_{{\bar{\rho }}}$ is a local complete intersection.

As we will discuss in detail later, this is equivalent to that the derived local Galois deformation functor constructed by [18] is classical. In other words, if $\mathcal {X}$ is the correct derived object to be considered, it is already infinitesimal classical at any point corresponding to finite fields.

Now let’s be more precise about the notion of calssicality.

### Definition 3.2

Let $\mathcal {F}: {\textbf {Nilp}}_{\mathbb {Z}_p}\rightarrow {\textbf {Ani}}$ be a derived prestack. We say $\mathcal {F}$ is classical if $\mathcal {F}$ is equivalent to the left Kan extension $\textrm{Lan}{^\textrm{cl}\mathcal {F}}$ of its underlying classical prestack $^\textrm{cl}\mathcal {F}$ along the natural inclusion ${Nilp}_{\mathbb {Z}_p}\hookrightarrow {\textbf {Nilp}}_{\mathbb {Z}_p}$. Equivalently, $\mathcal {F}$ is classical if $\mathcal {F}$ can be written as a colimit of $h_{A_i}$ where $A_i\in {Nilp}_{\mathbb {Z}_p}$. If $\mathcal {F}$ is a derived stack for the étale topology, we say $\mathcal {F}$ is classical as a stack if it is equivalent to the sheafification $(\textrm{Lan}{^\textrm{cl}\mathcal {F}})^{\#}$.

From now on, by left Kan extension, we always mean left Kan extension along the inclusion ${Nilp}_{\mathbb {Z}_p}\hookrightarrow {\textbf {Nilp}}_{\mathbb {Z}_p}$ unless specified otherwise. Our main result in this section is the following theorem.

### Theorem 3.3

The derived stack $\mathcal {X}$ is equivalent to the (étale) sheafification $(\textrm{Lan}{^\textrm{cl}\mathcal {X}})^{\#}$ of the left Kan extension $\textrm{Lan}{^\textrm{cl}\mathcal {X}}$ when restricted to ${\textbf {Nilp}}_{\mathbb {Z}_p}^{<\infty }$. In other words, $\mathcal {X}$ and $(\textrm{Lan}{\mathcal {X}_\textrm{EG}})^{\#}$ are equivalent after nilcompletion.

To prove Theorem 3.3, we need a global derived deformation theory instead of just infinitesimal deformation theory. This global theory is established in [16, Chapter 1]. Although [16] always assumes the base field is of characteristic 0, the foundational part of the deformation theory also works^6^ for animated rings (see Appendix I for some necessary modifications).

### Derived deformation theory

We first introduce the necessary foundational part of the derived deformation theory. The basic spirit is behind the following fact.

#### Fact 3.4

For any animated ring *R*, there is a homotopy pullback for any *n*3.1where $\tau _{\le n}(R)\oplus \pi _{n+1}(R)[n+2]$ is the split square-zero extension, which we will discuss later, the map $\tau _{\le n+1}R\rightarrow \tau _{\le n}R$ is the natural truncation map,  is the trivial section of the natural projection $\tau _{\le n}(R)\oplus \pi _{n+1}(R)[n+2]\rightarrow \tau _{\le n}R$, and the map $s:\tau _{\le n}R\rightarrow \tau _{\le n}(R)\oplus \pi _{n+1}(R)[n+2]$ is a non-trivial section.

Suppose $\mathcal {F}: {\textbf {Nilp}}_{\mathbb {Z}_p}\rightarrow {\textbf {Ani}}$ is a derived prestack. The study of the deformation theory of $\mathcal {F}$ can be divided into 3 parts:

1. Find the relationship between $\mathcal {F}(R)$ and $\{\mathcal {F}(\tau _{\le n}(R))\}_n$.
2. The homotopy pullback 3.1 is an example of square-zero extensions, which is controlled by (pro-)cotangent complex^7^ in a certain way. So we need to study the (pro-)cotangent complex of $\mathcal {F}$, which can be regarded as some “linear-algebra" data.
3. If we have a good understanding of the cotangent complex (or square zero extensions), then we need to understand how the derived prestack $\mathcal {F}$ acts on the square-zero extensions.

#### Nilcomplete

Let us start with the first part. Recall that all animated rings are Postnikov complete, i.e. $R\simeq \varprojlim _n\tau _{\le n}R$. We then have the following definition.

##### Definition 3.5

(Nilcomplete) A derived prestack $\mathcal {F}$ is nilcomplete^8^ if for any $R\in {\textbf {Nilp}}_{\mathbb {Z}_p}$, the map

$$ \mathcal {F}(R)\rightarrow \varprojlim _n \mathcal {F}(\tau _{\le n}R) $$

is an isomorphism. Let ${\textrm{PreStk}}^\textrm{nil}$ denote the $\infty$-category of nilcomplete derived prestacks. The inclusion ${\textrm{PreStk}}^\textrm{nil}\hookrightarrow {\textrm{PreStk}}$ admits a left adjoint given by the nilcompletion

$$ \mathcal {F}^\textrm{nil}(R):=\varprojlim _n \mathcal {F}(\tau _{\le n}R) $$

##### Example 3.6

All derived affine schemes are nilcomplete. In fact, more is true: all derived Artin stacks are nilcomplete. Note that this is not always true for ind-derived Artin stacks^9^ as filtered colimit does not necessarily commute with inverse limit.

##### Remark 3.7

The left Kan extensions of prestacks in ${{\textrm{PreStk}}}^{\le n}$ along the inclusion ${\textbf {Nilp}}_{\mathbb {Z}_p}^{\le n}\rightarrow {\textbf {Nilp}}_{\mathbb {Z}_p}$ does not necessarily lie in ${\textrm{PreStk}}^\textrm{nil}$. So the left Kan extension ${\textrm{Lan}}{^{{\textrm{cl}}}\mathcal {X}}$ and its sheafification $(\textrm{Lan}{^\textrm{cl}\mathcal {X}})^{\#}$ might not be nilcomplete. This is why we need to take nilcompletion in Theorem 3.3.

#### Pro-cotangent complex

Suppose *A* is a discrete animated ring and *M* is a discrete *A*-module. Then there is a classical construction $A\oplus M$ which is an *A*-algebra whose underlying *A*-module structure is the natural one and the ring structure is given by the following formula

$$ (a_1,m_1)\cdot (a_2,m_2)=(a_1a_2,a_2m_1+a_1m_2). $$

Then we can obtain

$$ {\textrm{Hom}}_{{\textrm{Mod}}(A)}(\Omega _{A/\mathbb {Z}},M)\cong \textrm{Der}_{\mathbb {Z}}(A,M)\cong {\textrm{Hom}}_{A}(A,A\oplus M). $$

For general $A\in {\textbf {Nilp}}_{\mathbb {Z}_p}$ and animated *A*-module *M*, there is a similar story. Recall that the $\infty$-category ${\textrm{Mod}}(A)$ of *A*-modules is defined to be the $\infty$-category ${\textrm{Mod}}(A^{\circ })$ where $A^{\circ }$ is the underlying connective $\mathbb {E}_{\infty }$-algebra of *A* through the natural functor $\Theta : {\textbf {Nilp}}_{\mathbb {Z}_p}\rightarrow {\textbf {CAlg}}_{\mathbb {Z}_p}^{\le 0}$ (cf. [28, Construction 25.1.2.1]). Then the $\infty$-category ${\textrm{Mod}}(A)^{\le 0}$ of animated *A*-modules is the connective part ${\textrm{Mod}}(A^{\circ })^{\le 0}$. Now one can still define $A\oplus M$ and the derivations $\textrm{Der}_{\mathbb {Z}}(A,M)$ of *A* with values in *M*. There is an isomorphism

$$ {\textrm{Hom}}_{A}(L_{A/\mathbb {Z}},M)\simeq \textrm{Der}_{\mathbb {Z}}(A,M), $$

where $L_{A/\mathbb {Z}}$ is the cotangent complex of *A* over $\mathbb {Z}$. For more information, we refer to [28, Chapter 25].

Now we present a theory of pro-cotangent complex of a derived prestack $\mathcal {F}:{\textbf {Nilp}}_{\mathbb {Z}_p}\rightarrow {\textbf {Ani}}$. Let $x\in \mathcal {F}(R)$ for some $R\in {\textbf {Nilp}}_{\mathbb {Z}_p}$. Then we define a functor ${\textrm{Mod}}(R)^{\le 0}\rightarrow {\textbf {Ani}}$ by

$$ \mathcal {F}_x:M\in {\textrm{Mod}}(R)^{\le 0}\mapsto \textrm{Fib}_x(\mathcal {F}(R\oplus M)\rightarrow \mathcal {F}(R)). $$

Let $M_1\rightarrow M_2$ be a map in ${\textrm{Mod}}(R)^{\le 0}$ such that $H^0(M_1)\rightarrow H^0(M_2)$ is a surjection. Consider the fiber product $M:=0\times _{M_2}M_1$ in ${\textrm{Mod}}(R)$. Then *M* is also in ${\textrm{Mod}}(R)^{\le 0}$. So we also get a fiber product $R\oplus M\simeq R\times _{(R\oplus M_2)}(R\oplus M_1)$ as the functor $R\oplus (-)$ commutes with limits.

##### Definition 3.8

(Pro-cotangent space at a point) We say $\mathcal {F}$ admits a pro-cotangent space at $x\in \mathcal {F}(R)$ if the diagram3.2induced by $R\oplus M\simeq R\times _{(R\oplus M_2)}(R\oplus M_1)$ is a homotopy pullback diagram for all $M_1\rightarrow M_2$ as above.

More generally, if $\mathcal {F}$ admits a pro-cotangent space at $x\in \mathcal {F}(R)$, then we can define a new functor ${\textrm{Mod}}(R)^{-}\rightarrow \mathcal S$ by

$$ M\in {\textrm{Mod}}(R)^{\le k}\mapsto \Omega ^i(\textrm{Fib}_x(\mathcal {F}(R\oplus M[i])\rightarrow \mathcal {F}(R)) $$

for any $i\ge k$, where ${\textrm{Mod}}(R)^{-}$ is the $\infty$-category of almost connective modules, i.e. all those *M* such that *M*[*k*] is connective for some *k*. Then this new functor is actually exact as the diagram 3.2 is a homotopy pullback. In particular, this means the new functor is pro-corepresentable by an object in $\textrm{Pro}({\textrm{Mod}}(R)^{-})$, which denoted $T^*_x(\mathcal {F})$. We call it the pro-cotangent space to $\mathcal {F}$ at *x*.

##### Definition 3.9

(Pro-cotangent space) We say a derived prestack $\mathcal {F}$ admits pro-cotangent spaces if it admits a pro-cotangent space at all points.

##### Example 3.10

[16, Proposition 3.2.6] All derived schemes admit pro-cotangent spaces, even cotangent spaces, which means the pro-object is indeed in ${\textrm{Mod}}(R)^{-}$ for any *R*-point. All derived ind-schemes also admit pro-cotangent spaces.

##### Example 3.11

If $\mathcal {F}=h_{A}$, the prestack representable by some $A\in {\textbf {Nilp}}_{\mathbb {Z}_p}$, then for any point $x\in \mathcal {F}(R)$, the pro-cotangent space $T^*_x(\mathcal {F})$ is exactly $L_{A/\mathbb {Z}}\otimes _{A,x}R$. In fact, by [25, Section 3.2], the classical definition of the cotangent complex using cofibrant resolution is the same as the one in Definition 3.8.

Next we want to discuss the nilcompleteness condition of pro-cotangent spaces, which is closely related to the nilcompleteness of derived prestacks.

##### Definition 3.12

For any $R\in {\textbf {Nilp}}_{\mathbb {Z}_p}$, we let $\textrm{Pro}({\textrm{Mod}}(R)^{-})^\textrm{nil}$ denote the full sub-category of $\textrm{Pro}({\textrm{Mod}}(R)^{-})$ spanned by the objects $\mathcal {H}$ viewed as a functor ${\textrm{Mod}}(R)^{-}\rightarrow {\textbf {Ani}}$, satisfying

$$\begin{aligned} \mathcal {H}(M)\xrightarrow {\simeq }\varprojlim _n\mathcal {H}(\tau _{\le n}M).^10 \end{aligned}$$

^10^

##### Lemma 3.13

[16, Chapter 1, Lemma 3.3.3] Let $\mathcal {F}$ be a nilcomplete derived prestack. If it admits a pro-cotangent space at $x\in \mathcal {F}(R)$, then $T^*_x(\mathcal {F})\in \textrm{Pro}({\textrm{Mod}}(R)^{-})^\textrm{nil}$.

##### Proof

This follows from the definition of nilcompleteness of derived prestack. $\square$

For any $R\in {\textbf {Nilp}}_{\mathbb {Z}_p}$, there is another full subcategory $\textrm{Pro}({\textrm{Mod}}(R)^{-})_\textrm{laft}$ of $\textrm{Pro}({\textrm{Mod}}(R)^{-})$, which is very important to us.

##### Definition 3.14

$\textrm{Pro}({\textrm{Mod}}(R)^{-})_\textrm{laft}$ is the full subcategory of $\textrm{Pro}({\textrm{Mod}}(R)^{-})$ spanned by objects $\mathcal {H}$ satisfying

1. $$\mathcal {H}\in \textrm{Pro}({\textrm{Mod}}(R)^{-})^\textrm{nil}$$;
2. For every $m\ge 0$, the induced functor ${\textrm{Mod}}(R)^{[-m,0]}\rightarrow {\textbf {Ani}}$ commutes with filtered colimits.

The full subcategory $\textrm{Pro}({\textrm{Mod}}(R)^{-})_\textrm{laft}$ will be closely related to the geometric property of the derived prestack. To see this, we now recall some basic properties concerning ${\textbf {Nilp}}_{\mathbb {Z}_p}$.

##### Definition 3.15

[25, Proposition 3.1.5], [17, Chapter 2, Corollary 1.5.6] Let $R\in {\textbf {Nilp}}_{\mathbb {Z}_p}^{\le n}$ for some *n*. Then we say *R* is of finite type (or finite presentation) over $\mathbb {Z}_p$ if it satisfies the following equivalent conditions.

1. The functor ${\textrm{Hom}}(R,-)$ commutes with filtered colimit when restricted to ${\textbf {Nilp}}_{\mathbb {Z}_p}^{\le n}$, i.e. *R* is a compact object in ${\textbf {Nilp}}_{\mathbb {Z}_p}^{\le n}$.
2. $\pi _0(R)$ is a finite type $\mathbb {Z}_p$-algebra and each $\pi _m(R)$ is a finite generated module over $\pi _0(R)$.

Let ${\textbf {Nilp}}_{\mathbb {Z}_p,\mathrm ft}^{\le n}$ denote the full subcategory consisting of animated rings satisfying the above condition. Then we have ${\textbf {Nilp}}_{\mathbb {Z}_p}^{\le n}\simeq \textrm{Ind}({\textbf {Nilp}}_{\mathbb {Z}_p,\mathrm ft}^{\le n})$.

Now we can define derived prestacks locally of almost finite type (or locally almost of finite presentation).

##### Definition 3.16

Let $\mathcal {F}\in {\textrm{PreStk}}^{\le n}$. We say $\mathcal {F}$ is locally of finite type over $\mathbb {Z}_p$ if $\mathcal {F}$ is the left Kan extension along the inclusion ${\textbf {Nilp}}_{\mathbb {Z}_p,\mathrm ft}^{\le n}\hookrightarrow {\textbf {Nilp}}_{\mathbb {Z}_p}^{\le n}$, or equivalently, if $\mathcal {F}$ preserves filtered colimit. We say $\mathcal {F}\in {\textrm{PreStk}}$ is locally almost of finite type over $\mathbb {Z}_p$ if it is nilcomplete and its restriction to ${\textbf {Nilp}}_{\mathbb {Z}_p}^{\le n}$ is locally of finite type over $\mathbb {Z}_p$ for every $n\ge 0$. Let ${\textrm{PreStk}}_\textrm{laft}$ denote the $\infty$-category of derived prestacks locally almost of finite type.

Now we have the following unsurprising result.

##### Lemma 3.17

[16, Chapter 1, Lemma 3.5.2] Let $\mathcal {F}$ be a derived prestack locally almost of finite type. Assume $\mathcal {F}$ admits a pro-cotangent space at $x\in \mathcal {F}(R)$. Then the pro-cotangent space $T^*_x(\mathcal {F})$ is in $\textrm{Pro}({\textrm{Mod}}(R)^{-})_\textrm{laft}$.

##### Proof

This follows from the definition of ${\textrm{PreStk}}_\textrm{laft}$ and that the functor $(R,M)\mapsto R\oplus M$ commutes with filtered colimit. $\square$

We conclude this subsection with the functoriality of pro-cotangent space. Let $R_1\rightarrow R_2$ be a map in ${\textbf {Nilp}}_{\mathbb {Z}_p}$. Then we have a natural pullback functor

$$ f^*: {\textrm{Mod}}(R_1)^{-}\rightarrow {\textrm{Mod}}(R_2)^{-} $$

which induces a natural functor

$$ {\textrm{Pro}}(f^*):{\textrm{Pro}}({\textrm{Mod}}(R_1)^{-})\rightarrow {\textrm{Pro}}({\textrm{Mod}}(R_2)^{-}). $$

Now let $x_1\in \mathcal {F}(R_1)$ be an $R_1$-point and $x_2\in \mathcal {F}(R_2)$ be the image of the point $x_1$ in $\mathcal {F}(R_2)$. Assuming $\mathcal {F}$ admits pro-cotangent spaces at $x_1$ and $x_2$, we can obtain a map

$$ T_{x_2}^*(\mathcal {F})\rightarrow {\textrm{Pro}}(f^*)(T_{x_1}^*(\mathcal {F})) $$

##### Definition 3.18

(Pro-cotangent complex) We say $\mathcal {F}$ admits a (pro)-cotangent complex if it admits (pro)-cotangent spaces and for any $R_1\rightarrow R_2, x_1,x_2$ as above, the map $T_{x_2}^*(\mathcal {F})\rightarrow {\textrm{Pro}}(f^*)(T_{x_1}^*(\mathcal {F}))$ is an isomorphism.

##### Example 3.19

[16, Proposition 7.4.2] All the derived Artin stacks admit (pro)-cotangent complexes. For derived affine schemes, this can be easily seen from Example 3.11.

#### Infinitesimally cohesive

As we have seen in Fact 3.4, there is a homotopy pullback diagram connecting $\tau _{\le n+1}R$ and $\tau _{\le n}R$ for $R\in {\textbf {Nilp}}_{\mathbb {Z}_p}$ and each $n\ge 0$. This homotopy pullback diagram is an example of more general square-zero extensions.

##### Definition 3.20

(Square-zero extension) Let $R\in {\textbf {Nilp}}_{\mathbb {Z}_p}$ and *M* is a connective *R*-module. A square-zero extension of *R* by *M* is a homotopy pullback diagramwhere $\textrm{triv}: R\rightarrow R\oplus M[1]$ is the trivial section of the natural projection $R\oplus M[1]\rightarrow R$ and *s* is any section of $R\oplus M[1]\rightarrow R$. We simply write the square-zero extension as the quadruple $({\tilde{R}},R,M,s)$.

By the definition of the cotangent complex $L_{R/\mathbb {Z}}$, we see that square-zero extensions are classified by ${\textrm{Hom}}(L_{R/\mathbb {Z}},M[1])$.

##### Definition 3.21

(Infinitesimally cohesive) Let $\mathcal {F}$ be a derived prestack. We say $\mathcal {F}$ is infinitesimally cohesive if for all square-zero extensions $({\tilde{R}},R,M,s)$, the following diagram is a homotopy pullback

### Deformation theory of $\mathcal {X}$

After making all the necessary preparations, we are now at a good stage to study the derived stack $\mathcal {X}$. The three properties about derived prestacks we discussed in previous subsections can be integrated into the following definition.

#### Definition 3.22

(Deformation theory) Let $\mathcal {F}$ be a derived prestack. We say $\mathcal {F}$ admits a deformation theory (resp. corepresentable deformation theory) if the following conditions are satisfied:

1. it is nilcomplete;
2. it admits pro-cotangent complex (resp. cotangent complex^11^);
3. it is infinitesimal cohesive.

There is a simple way to verify if a derived prestack admits a deformation theory.

#### Proposition 3.23

[16, Chapter 1, Proposition 7.2.5] Suppose $\mathcal {F}$ is a nilcomplete derived prestack. If for any homotopy pullback diagram of truncated animated ringswith $S_1\rightarrow S_0$ a nilpotent embedding, which means $\pi _0(S_1)\rightarrow \pi _0(S_0)$ is surjective and its kernel is a nilpotent ideal, the induced diagramis also a homotopy pullback diagram. Then $\mathcal {F}$ admits a deformation theory.

#### Proof

As $\mathcal {F}$ is nilcomplete, it is sufficient to check the existence of pro-cotangent complexes and infinitesimal cohesiveness for truncated animated rings and modules by [16, Chapter 1, Lemma 3.3.4 and 6.1.3]. So considering truncated animated rings is sufficient for the arguments in the proof of [16, Chapter 1, Proposition 7.2.5]. $\square$

We also have its converse.

#### Proposition 3.24

[16, Chapter 1, Proposition 7.2.2] Suppose $\mathcal {F}$ admits a deformation theory. Then for any homotopy pullback diagram $S\simeq S_1\times _{S_0}S_2$ with $S_1\rightarrow S_0$ a nilpotent embedding, the induced map $\mathcal {F}(S)\rightarrow \mathcal {F}(S_1)\times _{\mathcal {F}(S_0)}\mathcal {F}(S_2)$ is an equivalence.

We will actually use this criterion to prove $\mathcal {X}^\textrm{nil}$ admits a deformation theory.

#### Proposition 3.25

The derived prestack $\mathcal {X}^\textrm{nil}$ admits a deformation theory.

In order to prove Proposition 3.25, we recall the following theorem due to Toën–Vaquié. Note that all derived Artin stacks admit a deformation theory by [16, Proposition 7.4.2].

#### Theorem 3.26

[35, 37] The derived (pre)stack ${\textbf {Perf}}^{[a,b]}:{\textbf {Nilp}}_{\mathbb {Z}_p}\rightarrow {\textbf {Ani}}$ sending $R\in {\textbf {Nilp}}_{\mathbb {Z}_p}$ to $\textrm{Perf}(R)^{[a,b],\simeq }$ admits a deformation theory and is locally almost of finite presentation for any $a\le b\in \mathbb {Z}$. In particular, the derived (pre)stack $\bigsqcup _n \textrm{BGL}_n$ of vector bundles admits a deformation theory and is locally almost of finite presentation.

#### Remark 3.27

In fact, we only need the Artin stack $\textrm{BGL}_n$. But for the possible generalisation to perfect complexes, we choose to present the above general theorem.

#### Proof of Proposition 3.25

Recall that for any animated ring $A\in {\textbf {Nilp}}_{\mathbb {Z}_p}$ equipped with a Frobenius action $\varphi$, we can define the $\infty$-groupoid of étale $\varphi$-modules over *A* by the homotopy pullback diagram3.3Using the Breuil–Kisin prism $(\mathfrak {S},(E))$ and Lemma 2.14, for any $R\in {\textbf {Nilp}}_{\mathbb {Z}_p}^{\le n}$, we haveNow we want to use Proposition 3.23. Given any homotopy fiber product $R\simeq R_1\times _{R_0}R_2$ with $R_1\rightarrow R_0$ a nilpotent embedding, we want to proveis also a homotopy pullback diagram for any $0\le i\le n+2$. By diagram 3.3, Theorem 3.26 and Proposition 3.24, we just need to prove (1)is also a homotopy pullback diagram and (2) $\mathfrak {S}_{R_1}^i[\frac{1}{E}]\rightarrow \mathfrak {S}_{R_0}^i[\frac{1}{E}]$ is a nilpotent embedding.

We first prove the first statement. In fact, it suffices to prove the natural map

$$ \iota :\mathfrak {S}^i/E^n\otimes R\rightarrow (\mathfrak {S}^i/E^n\otimes R_1)\times _{(\mathfrak {S}^i/E^n\otimes R_0)}(\mathfrak {S}^i/E^n\otimes R_2)$$

is an isomorphism for each *n*. As the forgetful functor

$$ \tilde{\Theta }:{\textbf {Nilp}}_{\mathbb {Z}_p}\xrightarrow {\Theta }  {\textbf {CAlg}}_{\mathbb {Z}_p}^{\le 0}\rightarrow {\textrm{Mod}}(\mathbb {Z}_p)^{\le 0} $$

is conservative and preserves limits (cf. [28, Proposition 25.1.2.2]), it remains to prove ${\tilde{\Theta }}(\iota )$ is an isomorphism.

Let ${\tilde{R}}$ denote the fiber product $R_1\times _{R_0}R_2$ in ${\textrm{Mod}}(\mathbb {Z}_p)$. Then we have ${\tilde{\Theta }}(R)$ is isomorphic to $\tau _{\ge 0}{\tilde{R}}$, the connective cover of ${\tilde{R}}$. As $\mathfrak {S}^i/E^n$ is flat over $\mathbb {Z}_p$, we have $\mathfrak {S}^i/E^n\otimes \tau _{\ge 0}{\tilde{R}}\simeq \tau _{\ge 0}(\mathfrak {S}^i/E^n\otimes \tilde{R})$. Note that fiber sequence in ${\textrm{Mod}}(\mathbb {Z}_p)$ is also a cofiber sequence. We then getis a homotopy pullback diagram in ${\textrm{Mod}}(\mathbb {Z}_p)$. Now by using $\tau _{\ge 0}$ commutes with limits, we see that ${\tilde{\Theta }}(\iota )$ is an isomorphism. So the first statement is true.

Next we prove the second statement, i.e. $\mathfrak {S}_{R_1}^i[\frac{1}{E}]\rightarrow \mathfrak {S}_{R_0}^i[\frac{1}{E}]$ is a nilpotent embedding. This means we need to prove the map $\pi _0(\mathfrak {S}^i{\widehat{\otimes }} R_1)\rightarrow \pi _0(\mathfrak {S}^i{\widehat{\otimes }} R_0))$ is surjective and it kernel is nilpotent. By Lemma 3.28, we see that $\pi _0(\mathfrak {S}^i{\widehat{\otimes }} R_j))\cong \mathfrak {S}^i{\widehat{\otimes }}\pi _0(R_j)$ for $j=0,1$, from which the second statement follows. In fact, let *K* be the kernel of $\pi _0(R_1)\rightarrow \pi _0(R_0)$. Then we have a short exact sequence

$$ 0\rightarrow \mathfrak {S}^i{\widehat{\otimes }} K\rightarrow \mathfrak {S}^i{\widehat{\otimes }} \pi _0(R_1)\rightarrow \mathfrak {S}^i{\widehat{\otimes }} \pi _0(R_0)\rightarrow 0 $$

where $\mathfrak {S}^i{\widehat{\otimes }} K$ is nilpotent.

#### Lemma 3.28

Let *A* be a flat $\mathbb {Z}/p^a$-algebra for some $a>0$. Let $d\in A$ be a non zero-divisor such that $A/d^n$ is flat over $\mathbb {Z}/p^a$ for all *n*. Let *R* be an animated ring over $\mathbb {Z}/p^a$. Then for each $i\ge 0$, we have $\pi _i(A{\widehat{\otimes }} R)\cong A{\widehat{\otimes }}\pi _i(R)$, where the completion is derived *d*-adic completion.

#### Proof

By [34, Tag 0CQE], we have a short exact sequence

$$ 0\rightarrow R^1\varprojlim _n \pi _{i+1}(A/d^n\otimes R)\rightarrow \pi _i(A{\widehat{\otimes }} R)\rightarrow \varprojlim _n \pi _i(A/d^n\otimes R)\rightarrow 0. $$

As $R^1\varprojlim _n \pi _{i+1}(A/d^n\otimes R)=R^1\varprojlim _n A/d^n\otimes \pi _{i+1}(R)=0$, we then have $\pi _i(A{\widehat{\otimes }} R)\cong \varprojlim _n \pi _i(A/d^n\otimes R)=A{\widehat{\otimes }} \pi _i(R)$. $\square$

We can also prove $({\textrm{Lan}}{^\textrm{cl}\mathcal {X}})^{\#,\mathrm nil}$ admits a deformation theory.

#### Theorem 3.29

The derived prestack $({\textrm{Lan}}\mathcal {X}_\textrm{EG})^{\#,\mathrm nil}$ admits a deformation theory.

#### Proof

Recall that $\mathcal {X}_\textrm{EG}$ is an ind-Artin stack. Write $\mathcal {X}_\textrm{EG}=\varinjlim _i\mathcal {X}_i$. As left Kan extension preserves colimits, we have ${\textrm{Lan}}\mathcal {X}_\textrm{EG}=\varinjlim _i{\textrm{Lan}}\mathcal {X}_i$. Then $({\textrm{Lan}}\mathcal {X}_\textrm{EG})^{\#}\simeq (\varinjlim _i({\textrm{Lan}}\mathcal {X}_i)^{\#})^{\#}$. By [17, Chapter 2, Proposition 4.4.3], we see that each $({\textrm{Lan}}\mathcal {X}_i)^{\#}$ is a derived Artin stack. By [17, Chapter 1, Corollary 4.3.4], $({\textrm{Lan}}\mathcal {X}_i)^{\#}(R)$ is in ${\textbf {Ani}}^{<\infty }$ for any truncated animated ring *R*. For any étale cover $R_1\rightarrow R_2$, if $R_1\in {\textbf {Ani}}^{<n}$, then $R_2$ is also in ${\textbf {Ani}}^{<n}$ as $\pi _i(R_2)\cong \pi _0(R_2)\otimes _{\pi _0(R_1)}\pi _i(R_1)$. Then by Lemma 2.14 and the fact that filtered colimit commutes with finite limit, we see $(\varinjlim _i({\textrm{Lan}}\mathcal {X}_i)^{\#})(R_1)\simeq \varprojlim (\varinjlim ({\textrm{Lan}}\mathcal {X}_i)^{\#})(R^{\bullet }_2)$ where the right hand side is the limit of the Čech nerve. In other words, this means $\varinjlim _i({\textrm{Lan}}\mathcal {X}_i)^{\#}$ already satisfies the étale sheaf property for étale coverings in ${\textbf {Ani}}^{<\infty }$. So $(\varinjlim _i({\textrm{Lan}}\mathcal {X}_i))^{\#}$ coincides with $\varinjlim _i({\textrm{Lan}}\mathcal {X}_i)^{\#}$ when restricted to truncated animated rings, which in particular satisfies the assumptions in Proposition 3.23 except being nilcomplete by [16, Proposition 7.4.2]. Then $(\varinjlim _i({\textrm{Lan}}\mathcal {X}_i))^{\#,\textrm{nil}}$ satisfies all the assumptions in Proposition 3.23. Hence $({\textrm{Lan}}\mathcal {X}_\textrm{EG})^{\#,\textrm{nil}}$ admits a deformation theory. $\square$

### Classicality of $\mathcal {X}$: Part 1

Now we have proved both $\mathcal {X}^\textrm{nil}$ and $({\textrm{Lan}}\mathcal {X}_\textrm{EG})^{\#,\mathrm nil}$ admit a deformation theory. The next goal is to compare them. The following proposition will be our key tool to compare two derived prestacks.

#### Proposition 3.30

[16, Chapter 1, Proposition 8.3.2] Let $\mathcal {F}_1\rightarrow \mathcal {F}_2$ be a map of derived prestacks admitting a deformation theory. Suppose there exists a commutative diagram,where $g_1,g_2$ are nilpotent embeddings (see [16, Definition 8.1.3]), and $^\textrm{cl}\mathcal {F}_{0}$ is a classical prestack. Suppose also that for any discrete commutative ring $R\in {Nilp}_{\mathbb {Z}_p}$, and a map $x_0\in {^\textrm{cl}\mathcal {F}_{0}}(R)$ and $x_i=g_i\circ x_0$ where $i=1,2$, the induced map

$$ T^*_{x_2}(\mathcal {F}_2)\rightarrow T^*_{x_1}(\mathcal {F}_1) $$

is an isomorphism. Then *f* is an isomorphism.

In our case, we will take $^\textrm{cl}\mathcal {F}_0=\mathcal {X}_\textrm{EG}$, $\mathcal {F}_1=({\textrm{Lan}}\mathcal {X}_\textrm{EG})^{\#,\mathrm nil}$ and $\mathcal {F}_2=\mathcal {X}^\textrm{nil}$. This means we need to compare $T^*_x(({\textrm{Lan}}\mathcal {X}_\textrm{EG})^{\#,\mathrm nil})$ and $T^*_x(\mathcal {X}^\textrm{nil})$ for any discrete $R\in \textrm{Nilp}_{\mathbb {Z}_p}$. We will divide this into two parts. In this section, we will see how to reduce the comparison of pro-cotangent complexes at arbitrary points to those valued in finite fields. In the next section, we will continue to compare pro-cotangent complexes at finite field points and finish the proof.

#### Locally almost of finite type

It is not easy to compare pro-cotangent spaces for arbitrary classical rings. But Lemma 3.17 says that the situation becomes better if the involved derived prestacks are locally almost of finite type. This is what we will prove.

##### Proposition 3.31

The derived prestack $\mathcal {X}^\textrm{nil}$ is locally almost of finite type.

##### Proof

By definition, this means we need to prove $\mathcal {X}^\textrm{nil}$ preserves filtered colimit when restricted to ${\textbf {Nilp}}_{\mathbb {Z}_p}^{\le n}$ for each $n\ge 0$. Write $R=\varinjlim _iR_i\in {\textbf {Nilp}}_{\mathbb {Z}_p}^{\le n}$ as a filtered colimit. We then need to prove $\varinjlim _i\mathcal {X}(R_i)\simeq \mathcal {X}(R)$.

Again by using the Breuil–Kisin prism $(\mathfrak {S},(E)$ and Lemma 2.14, we haveAs filtered colimit commutes with finite limit in ${\textbf {Ani}}$, we have

$$ \varinjlim _i\mathcal {X}(R_i)\simeq \varprojlim (\varinjlim _i{\textrm{Vect}}(\mathfrak {S}_{R_i}[\frac{1}{E}])^{\varphi =1,\simeq })\rightrightarrows (\varinjlim _i{\textrm{Vect}}(\mathfrak {S}^1_{R_i}[\frac{1}{E}])^{\varphi =1,\simeq })\cdots (\varinjlim _i{\textrm{Vect}}(\mathfrak {S}^{n+2}_{R_i}[\frac{1}{E}])^{\varphi =1,\simeq }). $$

We claim that the functor $\varinjlim _i{\textrm{Vect}}(\mathfrak {S}^{j}_{R_i}[\frac{1}{E}])^{\varphi =1}\rightarrow {\textrm{Vect}}(\mathfrak {S}^{j}_{R}[\frac{1}{E}])^{\varphi =1}$ is fully faithful for every $0\le j\le n+2$. In fact, let $M_s\in {\textrm{Vect}}(\mathfrak {S}^{j}_{R_s}[\frac{1}{E}])^{\varphi =1}$ and $M_t\in {\textrm{Vect}}(\mathfrak {S}^{j}_{R_t}[\frac{1}{E}])^{\varphi =1}$. Then the mapping space between $M_s$ and $M_t$ in $\varinjlim _i{\textrm{Vect}}(\mathfrak {S}^{j}_{R_i}[\frac{1}{E}])^{\varphi =1}$ is

$$ \varinjlim _{s\rightarrow k,t\rightarrow k}\textrm{Map}_k(M_{s,k},M_{t,k})$$

where $M_{s,k}=M_s\otimes _{R_s}R_k$, $M_{t,k}=M_t\otimes _{R_t}R_k$ and $\textrm{Map}_k(-,-)$ means the mapping space in ${\textrm{Vect}}(\mathfrak {S}^{j}_{R_k}[\frac{1}{E}])^{\varphi =1}$. Then we need to prove $\varinjlim _{s,t\rightarrow k}\textrm{Map}_k(M_{s,k},M_{t,k})\simeq \textrm{Map}(M_{s,R},M_{t,R})$ where $M_{s,R}=M_s\otimes _{R_s}R$, $M_{t,R}=M_t\otimes _{R_t}R$ and the mapping space is taken in ${\textrm{Vect}}(\mathfrak {S}^{j}_{R}[\frac{1}{E}])^{\varphi =1}$. By [11, Lemma 5.2.14], we may assume $M_s$ and $M_t$ are both finite free. Choose a basis ${\underline{e}}$ of $M_s^{\vee }$. As $\varphi ^*(\mathfrak {S}_R^j {\underline{e}})$ is finite free, we have $\textrm{Map}(\varphi ^*(\mathfrak {S}_R^j \underline{e}),M_s^{\vee })=\textrm{Map}(\varphi ^*(\mathfrak {S}_R^j \underline{e}),(\mathfrak {S}_R^j{\underline{e}})[\frac{1}{u}])=\varinjlim _a \textrm{Map}(\varphi ^*(\mathfrak {S}_R^j\underline{e}),\frac{1}{u^a}\mathfrak {S}^j_R{\underline{e}})$. This means we can choose a $\varphi$-stable lattice ${\overline{M}}^{\vee }_s$ in $M_s^{\vee }$. In the same way, we choose a $\varphi$-stable lattice ${\overline{M}}_t$ in $M_t$. Note that $M_{s,k}^{\vee }\simeq M_{s}^{\vee }\otimes _{R_s}R_k$ and $M_{s,R}^{\vee }\simeq M_s^{\vee }\otimes _{R_s}R$. Now we just need to prove $\varinjlim _{s,t\rightarrow k}(M_{s,k}^{\vee }\otimes M_{t,k})^{\varphi =1}\simeq (M_{s,R}^{\vee }\otimes M_{t,R})^{\varphi =1}$. This follows from Lemma 2.25.

Next we claim that the functor $\varinjlim _i{\textrm{Vect}}(\mathfrak {S}^{}_{R_i}[\frac{1}{E}])^{\varphi =1,\simeq }\rightarrow {\textrm{Vect}}(\mathfrak {S}^{}_{R}[\frac{1}{E}])^{\varphi =1,\simeq }$ is also essentially surjective. By Lemma 2.23, this will then complete the proof of Proposition 3.31.

Note that for any *i* and *n*, we have the following homotopy pullback diagram as in Fact 3.43.4By tensoring with $\mathfrak {S}/E^m$ which is flat over $\mathbb {Z}_p$, we get a homotopy pullback diagram compatible with $\varphi$-actions, which come from the $\varphi$-actions on $\mathfrak {S}$,3.5Now by taking limit with respect to *m* and inverting *E*, we get a homotopy pullback diagram compatible with $\varphi$-actions3.6Here we have used the fact that taking truncation commutes with limits and the fact that $\pi _{n+1}(\mathfrak {S}_{R_i})\cong \varprojlim _m \pi _{n+1}(\mathfrak {S}/E^m\otimes R_i)$ by Lemma 3.28.

Note that Diagram 3.6 is exactly the diagram obtained by applying Fact 3.4 to $\mathfrak {S}_{R_i}$, which is a square-zero extension. The point is that now we know Diagram 3.6 (especially the section *s*) is compatible with $\varphi$-actions and the Frobenius on $\tau _{\le n+1}{\mathfrak {S}_{R_i}}[\frac{1}{E}]$ is induced by the homotopy pullback diagram. In particular, it induces a homotopy pullback diagram3.7by Theorem 3.26 and the fact that taking $(-)^{\varphi =1,\simeq }$ commutes with homotopy pullbacks.

Now we want to prove the left vertical functor in Diagram 3.7 is essentially surjective, which can be deduced from that the right vertical functor is essentially surjective. Consider the base change functor between the homotopy categoriesGiven $(M,\varphi _M)\in \textrm{Ho}({\textrm{Vect}}(\tau _{\le n}(\mathfrak {S}_{R_i}[\frac{1}{E}])\oplus \pi _{n+1}(\mathfrak {S}_{R_i}[\frac{1}{E}])[n+2]))^{\varphi =1})$, by base changing along the natural projection $\tau _{\le n}(\mathfrak {S}_{R_i}[\frac{1}{E}])\oplus \pi _{n+1}(\mathfrak {S}_{R_i}[\frac{1}{E}])[n+2]\rightarrow \tau _{\le n}(\mathfrak {S}_{R_i}[\frac{1}{E}])$, we get $(M_1,\varphi _{M_1})$. Further base changing along the trivial section $\textrm{triv}: \tau _{\le n}(\mathfrak {S}_{R_i}[\frac{1}{E}])\rightarrow \tau _{\le n}(\mathfrak {S}_{R_i}[\frac{1}{E}])\oplus \pi _{n+1}(\mathfrak {S}_{R_i}[\frac{1}{E}])[n+2]$, we get $(M_2,\varphi _{M_2})\in \textrm{Ho}({\textrm{Vect}}(\tau _{\le n}(\mathfrak {S}_{R_i}[\frac{1}{E}])\oplus \pi _{n+1}(\mathfrak {S}_{R_i}[\frac{1}{E}])[n+2]))^{\varphi =1})$. As the trivial section and the natural projection $\tau _{\le n}(\mathfrak {S}_{R_i}[\frac{1}{E}])\oplus \pi _{n+1}(\mathfrak {S}_{R_i}[\frac{1}{E}])[n+2]\rightarrow \tau _{\le n}(\mathfrak {S}_{R_i}[\frac{1}{E}])$ induce the identity on $\pi _0$, we see that $(\pi _0(M),\varphi _{M})=(\pi _0(M_2),\varphi _{M_2})$. This must give an isomorphism between $(M,\varphi _M)$ and $(M_2,\varphi _{M_2})$ by Lemma 3.32, which finishes the proof of the essential surjectivity of the base change functor

$$\begin{aligned} {\textrm{Vect}}(\tau _{\le n+1}(\mathfrak {S}_{R_i}[\frac{1}{E}])^{\varphi =1,\simeq }\rightarrow {\textrm{Vect}}(\tau _{\le n}(\mathfrak {S}_{R_i}[\frac{1}{E}]))^{\varphi =1,\simeq }. \end{aligned}$$

We then get the essential surjectivity of the functor $\pi _0$

$$ {\textrm{Vect}}((\mathfrak {S}_{R_i}[\frac{1}{E}])^{\varphi =1,\simeq }\rightarrow {\textrm{Vect}}(\pi _0(\mathfrak {S}_{R_i}[\frac{1}{E}]))^{\varphi =1,\simeq }. $$

Let’s now look at the following commutative diagram of base change functorsBy the limit-preserving property of classical stack of étale $\varphi$-modules ( [12, Corollary 3.1.5 and 3.2.6]), we know the functor $l_4$ is an equivalence. By previous discussions, we also know $l_1$ is fully faithful and $l_2,l_3$ are essentially surjective. So given *M* in $\textrm{Ho}({\textrm{Vect}}(\mathfrak {S}_{R}[\frac{1}{E}])^{\varphi =1,\simeq })$, we can get *N* in $\textrm{Ho}(\varinjlim _i{\textrm{Vect}}(\mathfrak {S}_{R_i}[\frac{1}{E}])^{\varphi =1,\simeq })$ such that $l_3\circ l_1(N)\simeq l_3(M)$. By Lemma 3.32, this implies $l_1$ is essentially surjective, which finally finishes the proof of Proposition 3.31. $\square$

##### Lemma 3.32

Let *A* be an animated ring with a Frobenius action $\varphi$. Let $M,N\in {\textrm{Vect}}(A)^{\varphi =1}$. If there is an isomorphism $f:\pi _0(M)\rightarrow \pi _0(N)$ in ${\textrm{Vect}}(\pi _0(A))^{\varphi =1}$, then *f* can be lifted to an isomorphism ${\tilde{f}}: M\rightarrow N$ in $\textrm{Ho}({\textrm{Vect}}(A)^{\varphi =1})$.

##### Proof

Let *V* be an étale $\varphi$-module in ${\textrm{Vect}}(A)^{\varphi =1}$. We have an exact triangle

$$ V^{\varphi =1}\rightarrow V\xrightarrow {\varphi -1}V. $$

Then we see the natural map $\pi _0(V^{\varphi =1})\rightarrow (\pi _0(V))^{\varphi =1}$ is surjective. This means *f* can be lifted to a map ${\tilde{f}}:M\rightarrow N$ by taking $V=M^{\vee }\otimes N$. As a map between finite projective modules over *A* is an isomorphism if and only if the induced map on $\pi _0$ is an isomorphism, we see that ${\tilde{f}}$ is an isomorphism. $\square$

We can also prove a similar result for $({\textrm{Lan}}\mathcal {X}_\textrm{EG})^{\#,\mathrm nil}$.

##### Proposition 3.33

The derived prestack $({\textrm{Lan}}\mathcal {X}_\textrm{EG})^{\#,\mathrm nil}$ is locally almost of finite type.

##### Proof

Let $(({\textrm{Lan}}\mathcal {X}_\textrm{EG})^{\#})^{\le n}$ be the restriction of $({\textrm{Lan}}\mathcal {X}_\textrm{EG})^{\#}$ to ${\textbf {Nilp}}_{\mathbb {Z}_p}^{\le n}$. Let $({\textrm{Lan}}\mathcal {X}_\textrm{EG})^{\#,\le n}_\textrm{ft}$ be its restriction to ${\textbf {Nilp}}_{\mathbb {Z}_p,\mathrm ft}^{\le n}$. We need to prove $(({\textrm{Lan}}\mathcal {X}_\textrm{EG})^{\#})^{\le n}$ is the left Kan extension of $({\textrm{Lan}}\mathcal {X}_\textrm{EG})^{\#,\le n}_\textrm{ft}$ along the inclusion ${\textbf {Nilp}}_{\mathbb {Z}_p,\mathrm ft}^{\le n}\rightarrow {\textbf {Nilp}}_{\mathbb {Z}_p}^{\le n}$. By [17, Chapter 2, Corollary 2.5.7], we have $(({\textrm{Lan}}\mathcal {X}_\textrm{EG})^{\#})^{\le n}\simeq (({\textrm{Lan}}\mathcal {X}_\textrm{EG})^{\le n})^{\#_{n}}$ where $\#_{n}$ means the sheafification functor on the level of ${\textbf {Nilp}}_{\mathbb {Z}_p}^{\le n}$ (cf. [17, Chapter 2, Section 2.5.1]).

As $\mathcal {X}_\textrm{EG}$ is locally of finite type, we know $({\textrm{Lan}}\mathcal {X}_\textrm{EG})^{\textrm{nil}}$ is locally almost of finite type by [17, Chapter 2, Corollary 1.7.8]. Let $({\textrm{Lan}}\mathcal {X}_\textrm{EG})^{\le n}_\textrm{ft}$ be the restriction of $({\textrm{Lan}}\mathcal {X}_\textrm{EG})^{\le n}$ to ${\textbf {Nilp}}_{\mathbb {Z}_p,\mathrm ft}^{\le n}$. Then $({\textrm{Lan}}\mathcal {X}_\textrm{EG})^{\le n}$ is the left Kan extension of $({\textrm{Lan}}\mathcal {X}_\textrm{EG})^{\le n}_\textrm{ft}$ along the inclusion ${\textbf {Nilp}}_{\mathbb {Z}_p,\mathrm ft}^{\le n}\rightarrow {\textbf {Nilp}}_{\mathbb {Z}_p}^{\le n}$. By [17, Chapter 2, Crollary 2.7.5(b) and 2.7.10], we see $(({\textrm{Lan}}\mathcal {X}_\textrm{EG})^{\le n})^{\#_{n}}$ is the left Kan extension of $({\textrm{Lan}}\mathcal {X}_\textrm{EG})^{\#,\le n}_\textrm{ft}$ along the inclusion ${\textbf {Nilp}}_{\mathbb {Z}_p,\mathrm ft}^{\le n}\rightarrow {\textbf {Nilp}}_{\mathbb {Z}_p}^{\le n}$. $\square$

#### Cotangent spaces at finite type points

Now as both $({\textrm{Lan}}\mathcal {X}_\textrm{EG})^{\#,\mathrm nil}$ and $\mathcal {X}^\textrm{nil}$ are locally almost of finite type, it suffices to reduce Proposition 3.30 to classical points which are valued in finite type algebras. We have the following theorem.

##### Theorem 3.34

The pro-cotangent complexes of both $({\textrm{Lan}}\mathcal {X}_\textrm{EG})^{\#,\mathrm nil}$ and $\mathcal {X}^\textrm{nil}$ at classical points valued in finite type algebras are indeed complexes (instead of being just pro-complexes).

We first prove this for $({\textrm{Lan}}\mathcal {X}_\textrm{EG})^{\#,\mathrm nil}$. Let us begin with some discussion about the pro-cotangent complexs of formal schemes. Let ${\textrm{Spf}}(R)$ be a classical Noetherian affine formal scheme with *I*-adic topology. Let $(x_1,\cdots ,x_n)$ be a set of generators of *I*. The left Kan extension of ${\textrm{Spf}}(R)$ is then the filtered colimit $\varinjlim _m \textrm{Lan Spec}(R/I^m)$. Note that $\textrm{Lan Spec}(R/I^m)$ is again representable by ${\textrm{Spec}}(R/I^n)$.

On the other hand, we can define animated rings $R_m=R\otimes ^{\mathbb {L}}_{f_m,\mathbb {Z}[y_1,\cdots ,y_n]}\mathbb {Z}$ where the map $f_m:\mathbb {Z}[y_1,\cdots ,y_n]\rightarrow R$ sends each $y_i$ to $x_i^{2^m}$. Then we can consider the filtered colimit $\varinjlim _m R_m$ as derived prestacks.

##### Proposition 3.35

( [20, Proposition 2.1.4]) The natural morphism

$$ \varinjlim _m {\textrm{Spec}}(R/I^m)\rightarrow \varinjlim _m {\textrm{Spec}}(R_m) $$

is an equivalence.

We still use ${\textrm{Spf}}(R)$ to denote the above derived formal scheme $\varinjlim _m \textrm{Lan Spec}(R/I^m)$. Now let *A* be a finite type $\mathbb {Z}/p^a$-algebra and $x: {\textrm{Spec}}(A)\rightarrow {\textrm{Spf}}(R)$ be an *A*-point of ${\textrm{Spf}}(R)$. Then we have $\textrm{Map}({\textrm{Spec}}(A),{\textrm{Spf}}(R))=\varinjlim _m \textrm{Map}({\textrm{Spec}}(A),{\textrm{Spec}}(R_m))$.

In particular, we have $T^*_x({\textrm{Spf}}(R))=``\varprojlim _m"T^*_x({\textrm{Spec}}(R_m))=``\varprojlim _m" L_{R_m/\mathbb {Z}}\otimes ^{\mathbb {L}}_{R_m}A$ as pro-systems by the definition of pro-cotangent complex. Now we have the cotangent complex $L_{R/\mathbb {Z}}$ of *R*. Consider its derived *I*-adic completion ${\widehat{L}}_{R/\mathbb {Z}}$. We have a natural pro-system $``\varprojlim _m" L_{R/\mathbb {Z}}\otimes ^{\mathbb {L}}_{R}R_m$. For the given point *x* as above, we have the following result.

##### Lemma 3.36

Regarding ${\widehat{L}}_{R/\mathbb {Z}}\otimes ^{\mathbb {L}}_RA$ as a constant pro-complex, the natural morphism of pro-complexes $\widehat{L}_{R/\mathbb {Z}}\otimes ^{\mathbb {L}}_RA\rightarrow ``\varprojlim _m" L_{R/\mathbb {Z}}\otimes ^{\mathbb {L}}_{R}R_m\otimes ^{\mathbb {L}}_{R_m}A$ is an equivalence.

##### Proof

This is clear as

$$L_{R/\mathbb {Z}}\otimes ^{\mathbb {L}}_{R}R_m\otimes ^{\mathbb {L}}_{R_m}A\simeq L_{R/\mathbb {Z}}\otimes ^{\mathbb {L}}_RR/I^m\otimes ^{\mathbb {L}}_{R/I^m}A\simeq \widehat{L}_{R/\mathbb {Z}}\otimes ^{\mathbb {L}}_RR/I^m\otimes ^{\mathbb {L}}_{R/I^m}A\simeq \widehat{L}_{R/\mathbb {Z}}\otimes ^{\mathbb {L}}_RA.$$

$$\square $$

Consider the maps $\mathbb {Z}\rightarrow R\rightarrow R_m$. This induces an exact triangle

$$ L_{R/\mathbb {Z}}\otimes ^{\mathbb {L}}_RR_m\rightarrow L_{R_m/\mathbb {Z}}\rightarrow L_{R_m/R}, $$

which induces an exact triangle of pro-complexes

$$ ``\varprojlim _m"L_{R/\mathbb {Z}}\otimes ^{\mathbb {L}}_RR_m\otimes ^{\mathbb {L}}_{R_m}A\rightarrow ``\varprojlim _m" L_{R_m/\mathbb {Z}}\otimes ^{\mathbb {L}}_{R_m}A\rightarrow ``\varprojlim _m"L_{R_m/R}\otimes ^{\mathbb {L}}_{R_m}A $$

We claim the last term vanishes.

##### Lemma 3.37

The pro-complex $``\varprojlim _m"L_{R_m/R}\otimes ^{\mathbb {L}}_{R_m}A$ is isomorphic to 0.

##### Proof

Note that $R_m=R\otimes ^{\mathbb {L}}_{f_m,\mathbb {Z}[y_1,\cdots ,y_n]}\mathbb {Z}$. So

$$\begin{aligned}  &   L_{R_m/R}\simeq L_{\mathbb {Z}/\mathbb {Z}[y_1,\cdots ,y_n]}\otimes ^{\mathbb {L}}_{\mathbb {Z}[y_1,\cdots ,y_n],f_m}R\simeq L_{\mathbb {Z}/\mathbb {Z}[y_1,\cdots ,y_n]}\otimes ^{\mathbb {L}}_{\mathbb {Z}}R_m\\  &   \quad \simeq (y_1,\cdots ,y_n)/(y_1,\cdots ,y_n)^2[1]\otimes ^{\mathbb {L}}_{\mathbb {Z}}R_m. \end{aligned}$$

The transition map $R_m\rightarrow R_{m-1}$ is induced by the map $\mathbb {Z}[y_1,\cdots ,y_n]\rightarrow \mathbb {Z}[y_1,\cdots ,y_n]$ sending $y_i$ to $y_i^2$. So the transition map

$$ L_{R_m/R}\otimes ^{\mathbb {L}}_{R_m}A\rightarrow L_{R_{m-1}/R}\otimes ^{\mathbb {L}}_{R_{m-1}}A $$

turns into

$$ (y_1,\cdots ,y_n)/(y_1,\cdots ,y_n)^2[1]\otimes ^{\mathbb {L}}_{\mathbb {Z}}A\rightarrow (y_1,\cdots ,y_n)/(y_1,\cdots ,y_n)^2[1]\otimes ^{\mathbb {L}}_{\mathbb {Z}}A, $$

which sends $y_i$ to $y_i^2$. Hence the transition map is 0. So the pro-system $``\varprojlim _m"L_{R_m/R}\otimes ^{\mathbb {L}}_{R_m}A$ is 0. $\square$

##### Corollary 3.38

The pro-cotangent complex $T^*_x({\textrm{Spf}}(R))$ is isomorphic to ${\widehat{L}}_{R/\mathbb {Z}}\otimes ^{\mathbb {L}}_RA$, which is a complex of *A*-modules.

##### Proof

This follows from the above lemmas. $\square$

Now we are ready to prove Theorem 3.34 for $({\textrm{Lan}}\mathcal {X}_\textrm{EG})^{\#}$.

##### Proposition 3.39

Let $e:{\textrm{Spec}}(B)\rightarrow ({\textrm{Lan}}\mathcal {X}_\textrm{EG})^{\#}$ be a classical point with *B* being a finite type algebra over $\mathbb {Z}/p^a$ for some *a*. Then the pro-cotangent complex $T^*_{e}(({\textrm{Lan}}\mathcal {X}_\textrm{EG})^{\#})$ is a complex of *B*-modules.

##### Proof

Recall that $\mathcal {X}_\textrm{EG}$ is a Noetherian formal algebraic stack. This means there is a smooth surjective morphism $U\rightarrow \mathcal {X}_\textrm{EG}$ where *U* is a Noetherian affine formal scheme over ${\textrm{Spf}}(\mathbb {Z}_p)$.

Let $U^{\bullet }$ be the Čech nerve of the morphism $U\rightarrow \mathcal {X}_\textrm{EG}$. Then the étale sheafification $({\textrm{Lan}}(U^{\bullet }))^{\#}$ of the left Kan extension of $U^{\bullet }$ is a groupoid object, whose geometric realization in the $\infty$-category of derived stacks is exactly $({\textrm{Lan}}\mathcal {X}_\textrm{EG})^{\#}$. And $({\textrm{Lan}}U)^{\#}\rightarrow ({\textrm{Lan}}\mathcal {X}_\textrm{EG})^{\#}$ is again smooth surjective by [17, Chapter 2, Proposition 4.4.3].

As $({\textrm{Lan}}U)^{\#}\rightarrow ({\textrm{Lan}}\mathcal {X}_\textrm{EG})^{\#}$ is smooth surjective, there exists an étale covering $\{{\textrm{Spec}}(B_j)\}_{j\in J}$ of ${\textrm{Spec}}(B)$, with *J* a finite set, such that each $e_j:{\textrm{Spec}}(B_j)\rightarrow ({\textrm{Lan}}\mathcal {X}_\textrm{EG})^{\#}$ factors through $({\textrm{Lan}}U)^{\#}\rightarrow ({\textrm{Lan}}\mathcal {X}_\textrm{EG})^{\#}$. Then we have an exact triangle for each $j\in J$

$$ T^*_{e_j}(({\textrm{Lan}}\mathcal {X}_\textrm{EG})^{\#})\rightarrow T^*_{e_j}(({\textrm{Lan}}U)^{\#})\rightarrow T^*_{e_j}(({\textrm{Lan}}U)^{\#}/({\textrm{Lan}}\mathcal {X}_\textrm{EG})^{\#}) $$

where $T^*_{e_j}(({\textrm{Lan}}U)^{\#}/({\textrm{Lan}}\mathcal {X}_\textrm{EG})^{\#})$ is the relative pro-cotangent complex (cf. [16, Chapter 1, Section 2.4.3]) at the point $e_j$. As $({\textrm{Lan}}U)^{\#}$ is indeed a Noetherian affine formal scheme, we know $T^*_{e_j}(({\textrm{Lan}}U)^{\#})$ is a complex of $B_j$-modules by Corollary 3.38. Moreover as $({\textrm{Lan}}U)^{\#}\rightarrow ({\textrm{Lan}}\mathcal {X}_\textrm{EG})^{\#}$ is smooth surjective, the pro-complex $T^*_{e_j}(({\textrm{Lan}}U)^{\#}/({\textrm{Lan}}\mathcal {X}_\textrm{EG})^{\#})$ is also a complex of $B_j$-modules by [16, Proposition 7.4.2(b)]. Therefore, we see $T^*_{e_j}(({\textrm{Lan}}\mathcal {X}_\textrm{EG})^{\#})$ is also a complex of $B_j$-modules. By the next Lemma 3.40, the pro-cotangent complex $T^*_{e}(({\textrm{Lan}}\mathcal {X}_\textrm{EG})^{\#})$ is then a complex of *B*-modules. $\square$

##### Lemma 3.40

Let $``\varprojlim _{i\in I}"T_i$ be a pro-complex of *A*-modules. Let $\{B_j\}_{j\in J}$ be an étale covering of *A* where *J* is a finite set. Suppose that for each $j\in J$, the pro-complex $``\varprojlim _{i\in I}"T_i\otimes _AB_j$ is isomorphic to $P_j$, a complex of $B_j$-modules. Then the pro-complex $``\varprojlim _{i\in I}"T_i$ is indeed a complex of *A*-modules.

##### Proof

It suffices to prove: if $``\varprojlim _{i\in I}"T_i$ becomes 0 after base change along a faithfully flat map $A\rightarrow B$, then $``\varprojlim _{i\in I}"T_i$ itself is 0. By the assumption, for each $T_i\otimes _AB$, there exists $T_j$ such that there exists a map $T_j\rightarrow T_i$ such that $T_j\otimes _AB\rightarrow T_i\otimes _AB$ is the zero map. Then $T_j\rightarrow T_i$ is also the zero map. This means $``\varprojlim _{i\in I}"T_i=0$. $\square$

Next we move to prove Theorem 3.34 for $\mathcal {X}^\textrm{nil}$.

##### Proposition 3.41

Let $e:{\textrm{Spec}}(C)\rightarrow \mathcal {X}^{\textrm{nil}}$ be a classical point with *C* being a finite type algebra over $\mathbb {Z}/p^a$ for some *a*. Then the pro-cotangent complex $T^*_{e}(\mathcal {X}^{\textrm{nil}})$ is isomorphic to $\textrm{Herr}(ad(M_C))^\vee [-1]$ where $\textrm{Herr}(ad(M_C))^\vee$ is the dual of the Herr complex associated to the adjoint of the étale $(\varphi ,\Gamma )$-module $M_C$ corresponding to *e*.

##### Proof

Let $M\in {\textrm{Mod}}(C)^{[-n,0]}$ such that $M\simeq \tau ^{\ge -n}\tilde{M}$ for a perfect complex ${\tilde{M}}$. Then $C\oplus M\in {\textbf {Nilp}}_{\mathbb {Z}_p,\mathrm ft}^{\le n}$. By the definition of the pro-cotangent complex, we have the pullback diagramNote that the cotangent complex of the stack $\mathrm BGL_d$ at a point ${\textrm{Spec}}(D)\rightarrow \mathrm BGL_d$ is given by $ad(M_D)[-1]$, where $M_D$ is the corresponding finite projective module over *D* and $ad(M_D):=M_D\otimes M_D^{\vee }$. Then by Proposition 3.45 in the next subsection, we can use the perfect prismatic site and get

$$ \mathcal {X}^{\textrm{nil}}(C)\simeq \varprojlim _i \mathrm{BGL_d}(A^i_{{\textrm{cyc}},C}[\frac{1}{\xi }])^{\varphi =1} $$

and

$$ \mathcal {X}^{\textrm{nil}}(C\oplus M)\simeq \varprojlim _i \mathrm{BGL_d}(A^i_{{\textrm{cyc}},C\oplus M}[\frac{1}{\xi }])^{\varphi =1}. $$

Note that $A^i_{{\textrm{cyc}},C\oplus M}[\frac{1}{\xi }])=A^i_{{\textrm{cyc}},C}[\frac{1}{\xi }]\oplus (M\otimes _CA^i_{{\textrm{cyc}},C}[\frac{1}{\xi }])$. This implies

$$ \textrm{Map}(T^*_e(\mathcal {X}^{\textrm{nil}}),M)\simeq \varprojlim _i \textrm{Map}(T^*_{e_i}(\mathrm{BGL_d}),M\otimes _CA^i_{{\textrm{cyc}},C}[\frac{1}{\xi }])^{\varphi =1} $$

where $e_i:{\textrm{Spec}}(A^i_{{\textrm{cyc}},C}[\frac{1}{\xi }])\rightarrow \mathrm{BGL_d}$ is the point corresponding to *e*.

Also, we know that

$$\begin{aligned}  &   \varprojlim _i \textrm{Map}(T^*_{e_i}(\mathrm{BGL_d}),M\otimes _CA^i_{{\textrm{cyc}},C}[\frac{1}{\xi }])^{\varphi =1}\simeq \varprojlim _i\tau _{\ge 0}(ad(M_i)[1]\otimes _CM)^{\varphi =1}\\  &   \quad \simeq \tau _{\ge 0}\varprojlim _i (ad(M_i)[1]\otimes _CM)^{\varphi =1}, \end{aligned}$$

where $M_i$ is the corresponding object in $\mathrm{BGL_d}(A^i_{{\textrm{cyc}},C}[\frac{1}{\xi }])^{\varphi =1}$. In particular, by Lemma 2.22, we have

$$\begin{aligned} ad(M_i)=ad(M_0)\otimes _{A_{{\textrm{cyc}},C}[\frac{1}{\xi }]} A^i_{{\textrm{cyc}},C}[\frac{1}{\xi }]\cong C(\Gamma ^i,ad(M_0)). \end{aligned}$$

Recall that the homotopy limit of cosimplicial chain complexs is quasi-isomorphic to the totalisation of its corresponding double complex. Write $C(\Gamma ^{\bullet },ad(M_0))$ the complex associated to the cosimplicial module $\{ad(M_i)\}_i$. As each term $ad(M_i)$ is flat over *C*, we see that

$$ \varprojlim _i (ad(M_i)\otimes _CM)\simeq C(\Gamma ^{\bullet },ad(M_0))\otimes _C M $$

and therefore

$$ \varprojlim _i (ad(M_i)\otimes _CM)^{\varphi =1}\simeq C(\Gamma ^{\bullet },ad(M_0))^{\varphi =1}\otimes _C M. $$

Next we are going to study the complex $C(\Gamma ^{\bullet },ad(M_0))^{\varphi =1}$. We claim this complex is (a shift of) the Herr complex of the étale $(\varphi ,\Gamma )$-module $M_C$ corresponding to the point $e: {\textrm{Spec}}(C)\rightarrow \mathcal {X}^{\textrm{nil}}$. Note that $M_C$ corresponds to the étale $(\varphi ,\Gamma )$-modules $M_0$ over $A_{{\textrm{cyc}},C}[\frac{1}{\xi }]$ under the equivalence in Proposition 2.27. To prove the claim, we have to resort to the “basic" case introduced in [12], in which we can always find $(\varphi ,\Gamma )$-stable lattices.

Let us recall the following definition.

##### Definition 3.42

( [12] Definition 3.2.3) If *K* is a finite extension of $\mathbb {Q}_p$, we set $K^\textrm{basic}:=K\cap K_0(\zeta _{p^{\infty }})$, where $K_0$ is the maximal unramified extension of $\mathbb {Q}_p$ inside *K*.

The advantage of working with $K^\textrm{basic}$ is that $A^+_{K^\textrm{basic},C}$ is $(\varphi ,\Gamma )$-stable. Now the étale $(\varphi ,\Gamma )$-module $M_C$ of rank *d* over $A_{K,C}$ can be regarded as an étale $(\varphi ,\Gamma )$-module of rank $d[K:K^\textrm{basic}]$ over $A_{K^\textrm{basic},C}$ (cf. the discussion after [12, Definition 3.2.3]). By the same proof of [12, Lemma 5.1.5], we can find a $(\varphi ,\Gamma )$-stable $A^+_{K^\textrm{basic},C}$-lattice $\mathfrak {M}_C$ such that $ad(\mathfrak {M}_C)$ is a $(\varphi ,\Gamma )$-stable lattice in $ad(M_C)$. Let $(e_1,\cdots ,e_s)$ be a set of generators of $ad(\mathfrak {M}_C)$ over $A^+_{K^\textrm{basic},C}$. Note that $A^+_{K^\textrm{basic},C}$ is actually a subring of $A_{{\textrm{cyc}},C}$ (in fact, $A^+_{K^\textrm{basic},C}=A^+_{K_0,C}\subset A_{{\textrm{cyc}},C}$ by the discussion after [12, Deinition 2.1.12]). Therefore, the $A_{{\textrm{cyc}},C}$-submodule $ad(\mathfrak {M}_0)$ of $ad(M_0)$ generated by $(e_1,\cdots ,e_s)$ is a $(\varphi ,\Gamma )$-stable lattice.

Note that $A_{{\textrm{cyc}},C}[\frac{1}{\xi }]$ is a Noetherian Banach ring by [12, Proposition 2.4.3]. Then the forgetful functor from finite Banach modules over $A_{{\textrm{cyc}},C}[\frac{1}{\xi }]$ to finite $A_{{\textrm{cyc}},C}[\frac{1}{\xi }]$-modules is an equivalence of categories by [22, remark 2.2.11]. In particular, $ad(\mathfrak {M}_0)$ is an open subgroup of $ad(M_0)$ (this is true for any lattice). As $ad(M_0)$ is finite Banach module, we have

$$ ad(M_0)\cong \varprojlim _iad(M_0)/\xi ^iad(\mathfrak {M}_0). $$

Note that each $\xi ^iad(\mathfrak {M}_0)$ is again open in $ad(M_0)$. Hence $ad(M_0)/\xi ^iad(\mathfrak {M}_0)$ has discrete topology. By construction, we know $\mathfrak {M}_0$ is $\Gamma$-stable. As the $\Gamma$-action on $A_{{\textrm{cyc}}}$ preserves the ideal $(\xi )$, we know each $\xi ^i\mathfrak {M}_0$ is also $\Gamma$-stable. So the isomorphism $ad(M_0)\cong \varprojlim _iad(M_0)/\xi ^iad(\mathfrak {M}_0)$ actually writes the $\Gamma$-module $ad(M_0)$ as an inverse limit of discrete $\Gamma$-modules. In particular, we can apply [3, Lemma 7.3] to get

$$ C(\Gamma ^{\bullet },ad(M_0))\simeq [ad(M_0)\xrightarrow {\gamma -1}ad(M_0)]. $$

This implies

$$ C(\Gamma ^{\bullet },ad(M_0))^{\varphi =1}\simeq [ad(M_0)\xrightarrow {\gamma -1}ad(M_0)]^{\varphi =1}\simeq \textrm{Herr}(ad(M_0)) $$

where $\textrm{Herr}(ad(M_0))$ is the Herr complex associated with $ad(M_0)$ (see [12, Section 5.1]).

Recall that $M_0$ is the étale $(\varphi ,\Gamma )$-module over $A_{{\textrm{cyc}},C}[\frac{1}{\xi }]$ corresponding to $M_C$. We have a natural map

$$ \textrm{Herr}(ad(M_C))\rightarrow \textrm{Herr}(ad(M_0)). $$

By [33, Proposition 4.5], we have $H^i(\textrm{Herr}(ad(M_C)))$ is naturally isomorphic to the extension group ${\textrm{Ext}}^i(A_{K,C},ad(M_C))$ in the exact category of finite projective étale $(\varphi ,\Gamma )$-modules over $A_{K,C}$. The same proof actually applies to the exact category of finite projective étale $(\varphi ,\Gamma )$-modules over $A_{{\textrm{cyc}},C}[\frac{1}{\xi }]$. The only place that requires attention is the proof of [33, Lemma 4.10]: one needs to use that $A_{{\textrm{cyc}},C}$ is $\varphi$-stable and any $A_{{\textrm{cyc}},C}$-lattice in a finite projective étale $(\varphi ,\Gamma )$-module over $A_{{\textrm{cyc}},C}[\frac{1}{\xi }]$ is closed and complete. Hence we also get a natural isomorphism $H^i(\textrm{Herr}(ad(M_0))\cong {\textrm{Ext}}^i(A_{{\textrm{cyc}},C}[\frac{1}{\xi }],ad(M_0))$. Now as the natural map $A_{K,C}\rightarrow A_{{\textrm{cyc}},C}[\frac{1}{\xi }]$ is faithfully flat by [12, Proposition 2.2.12], the equivalence between the category of finite projective étale $(\varphi ,\Gamma )$-modules over $A_{K,C}$ and the category of finite projective étale $(\varphi ,\Gamma )$-modules over $A_{{\textrm{cyc}},C}[\frac{1}{\xi }]$ in [12, Proposition 2.7.8] is an exact equivalence. This then implies the natural map $\textrm{Herr}(ad(M_C))\rightarrow \textrm{Herr}(ad(M_0))$ is a quasi-isomorphism.

By [12, Theorem 5.1.22], we see that $\textrm{Herr}(ad(M_0))$ is indeed a perfect complex of *C*-modules. Then we have

$$ \textrm{Map}(T^*_e(\mathcal {X}^{\textrm{nil}}),M))\simeq \tau _{\ge 0}(\textrm{Herr}(ad(M_0))[1]\otimes _C M)\simeq \textrm{Map}(\textrm{Herr}(ad(M_0))^{\vee }[-1],M). $$

As $\textrm{Herr}(ad(M_0))^{\vee }[-1]$ itself is a perfect complex, the functor $\textrm{Map}(\textrm{Herr} (ad(M_0))^{\vee }[-1],-)$ commutes with filtered colimit. Then we have

$$ \textrm{Map}(T^*_e(\mathcal {X}^{\textrm{nil}}),M))\simeq \textrm{Map}(\textrm{Herr}(ad(M_0))^{\vee }[-1],M) $$

holds for any $M\in {\textrm{Mod}}(C)^{\le 0}$ since $M\simeq \varprojlim _n\tau ^{\ge -n}(M)$ and any $N\in {\textrm{Mod}}(C)^{[-n,0]}$ can be written as a filtered colimit $\varinjlim _jN_j$ such that $N_j$ is the truncation $\tau ^{\ge -n}{\tilde{N}}_j$ for some perfect complex $N_j$. By the uniqueness of the pro-cotangent complex, we see

$$\begin{aligned} T^*_e(\mathcal {X}^{\textrm{nil}})\simeq \textrm{Herr}(ad(M_0))^{\vee }[-1]\simeq \textrm{Herr}(ad(M_C))^{\vee }[-1]. \end{aligned}$$

In particular, $T^*_e(\mathcal {X}^{\textrm{nil}})$ is a perfect complex over *C*. $\square$

##### Proof of Theorem 3.34

This follows from Propositions 3.39 and 3.41.

Once we proved Theorem 3.34, we can be further reduced to considering only the classical points valued in finite fields.

##### Lemma 3.43

Let *x* be any finite-field point of $\mathcal {X}_\textrm{EG}$, i.e. $x\in \mathcal {X}_\textrm{EG}(k_f)$ where $k_f$ is a finite field. If the map

$$ f_x:T^*_x(\mathcal {X}^\textrm{nil})\rightarrow T^*_x(({\textrm{Lan}}\mathcal {X}_\textrm{EG})^{\#,\mathrm nil}) $$

is an isomorphism for all finite-field points, then the natural map $({\textrm{Lan}}\mathcal {X}_\textrm{EG})^{\#,\mathrm nil}\rightarrow \mathcal {X}^\textrm{nil}$ is an isomorphism.

##### Proof

For any classical ring $R\in \textrm{Nilp}_{\mathbb {Z}_p}$, write $R=\varinjlim _iR_i$ as filtered colimit of all finite type subalgebras $R_i$. Since $\mathcal {X}^\textrm{nil}$ is locally almost of finite type, we have $\mathcal {X}(R)=\varinjlim _i\mathcal {X}(R_i)$. So any point $x\in \mathcal {X}(R)$ is induced by a point $x\in \mathcal {X}(R_i)$. The same is true for $({\textrm{Lan}}\mathcal {X}_\textrm{EG})^{\#,\mathrm nil}$. As both of them admit pro-cotangent complexes, $f_y: T^*_y(\mathcal {X}^{\textrm{nil}})\rightarrow T^*_y(({\textrm{Lan}}\mathcal {X}_\textrm{EG})^{\#,\mathrm nil}$ is an isomorphism for any discrete ring $R\in {Nilp}_{\mathbb {Z}_p}$ and any $y\in \mathcal {X}_\textrm{EG}(R)$ if and only if $f_y$ is an isomorphism for any *R* and any *y* as above when $R\in {Nilp}_{\mathbb {Z}_p}$ runs over the sub-category of finite type objects.

Now let’s assume *R* is a finite type algebra over $\mathbb {Z}_p/p^a$ for some *a*. Then there is a faithfully flat covering $\bigsqcup _i\textrm{Spec}({\hat{R}}_{m_i})\rightarrow \textrm{Spec}(R)$ given by the disjoint union of the spectra of the completion of localizations of *R* at its maximal ideals. This enables us to reduce further to the points valued in complete Noetherian local rings with finite residue fields.

Now let’s assume *R* is a complete Noetherian local ring with maximal ideal *m* and residue field $k_f$ and $y\in ({\textrm{Lan}}\mathcal {X}_\textrm{EG})^{\#,\mathrm nil}(R)=\mathcal {X}_\textrm{EG}(R)$. By Proposition 3.39 and Proposition 3.41, we put $T^*_y(({\textrm{Lan}}\mathcal {X}_\textrm{EG})^{\#,\mathrm nil})=H\in {\textrm{Mod}}(R)$ and $T^*_y(\mathcal {X}^\textrm{nil})=K\in {\textrm{Mod}}(R)$. Then by Lemma 3.17 and Definition 3.14, to prove $f_y$ is an isomorphism, we just need to compare the cotangent spaces as filtered colimit preserving functors ${{\textrm{Mod}}}(R)^{[-n,0]}\rightarrow {\textbf {Ani}}$ for each $n\ge 0$. Note every object $M\in {{\textrm{Mod}}}(R)^{[-n,0]}$ can be written as a filtered colimit of perfect complexes $M\simeq \varinjlim _iK_i$. Then we see that $M\simeq \varinjlim _i\tau _{\le n}(\tau _{\ge 0}K_i)$ (we still use the homological convention here) as $\tau _{\le n}$ is a left adjoint and filtered colimit is exact. In particular, each $\tau _{\le n}(\tau _{\ge 0}K_i)$ is derived *m*-complete. So we just need to compare $H^{\wedge }_{m}$ and $K^{\wedge }_{m}$. By derived Nakayama lemma, we are reduced further to comparing $H\otimes k_f$ and $K\otimes k_f$, i.e. we just need to prove $f_y$ is an isomorphism for all finite-field points. $\square$

##### Remark 3.44

That the pro-cotangent complexes of the two derived stacks at classical points valued in finite type algebras are indeed complexes is crucial in the proof of Lemma 3.43. The point is that pro-complexes do not satisfy faithfully flat descent in general and the derived Nakayama lemma can not apply to pro-complexes.

### Classicalty of $\mathcal {X}$: Part 2

Now by Lemma 3.43, proving Theorem 3.3 can be reduced to comparing the infinitesimal deformation theory of $({\textrm{Lan}}\mathcal {X}_\textrm{EG})^{\#,\mathrm nil}$ and $\mathcal {X}^\textrm{nil}$, which will imply the pro-cotangent complexes are isomorphic. More precisely, fixing a finite-field point ${\bar{\rho }}\in \mathcal {X}_\textrm{EG}(k_f)$, i.e. a residual representation of the absolute Galois group $G_K$, we need to investigate the functor $({\textrm{Lan}}\mathcal {X}_\textrm{EG})^{\#,\mathrm nil}_{{\bar{\rho }}}: {{{\textbf {Art}}}_{/k_f}}\rightarrow {\textbf {Ani}}$ defined as

$$ R\mapsto \textrm{Fib}_{{\bar{\rho }}}(({\textrm{Lan}}\mathcal {X}_\textrm{EG})^{\#,\mathrm nil}(R)\rightarrow ({\textrm{Lan}}\mathcal {X}_\textrm{EG})^{\#,\mathrm nil}(k_f)), $$

and the functor $(\mathcal {X})_{{\bar{\rho }}}^{\textrm{nil}}: {{{\textbf {Art}}}_{/k_f}}\rightarrow {\textbf {Ani}}$ defined as

$$ R\mapsto \textrm{Fib}_{{\bar{\rho }}}(\mathcal {X}^{\textrm{nil}}(R)\rightarrow \mathcal {X}^{\textrm{nil}}(k_f)), $$

where ${{{\textbf {Art}}}}$ is the $\infty$-category of Artinian local animated ring and ${{{\textbf {Art}}}_{/k_f}}$ is the $\infty$-category of Artinian local animated ring *R* with an identification of $k_f$ with the residue field of *R*. Recall that an animated ring *R* is called Artinian local if $\pi _0(R)$ is a classical Artinian local ring and $\pi _*(R)$ is a finitely generated $\pi _0(R)$-module. In particular all Artinian local animated rings over $\mathbb {Z}_p$ are truncated. For any $M\in \textrm{Perf}(k_f)^{[-n,0]}$, we have $k_f\oplus M$ is an Artinian local animated ring. So if $({\textrm{Lan}}\mathcal {X}_\textrm{EG})^{\#}_{{\bar{\rho }}}=({\textrm{Lan}}\mathcal {X}_\textrm{EG})^{\#,\mathrm nil}_{{\bar{\rho }}}\simeq (\mathcal {X})^{\textrm{nil}}_{{\bar{\rho }}}=(\mathcal {X})_{\bar{\rho }}$, we can deduce $T^*_{{\bar{\rho }}}(\mathcal {X}^\textrm{nil})\rightarrow T^*_{\bar{\rho }}(({\textrm{Lan}}\mathcal {X}_\textrm{EG})^{\#,\mathrm nil})$ is an isomorphism by Definition 3.14.

In order to compare $({\textrm{Lan}}\mathcal {X}_\textrm{EG})^{\#}_{{\bar{\rho }}}$ and $\mathcal {X}_{{\bar{\rho }}}$ (we can omit the superscript $``\textrm{nil}"$), we will go back to derived Galois representations. Our proof will be divided into two parts:

1. compare $\mathcal {X}$ with the derived prestack of Laurent *F*-crystals on the absolute perfect prismatic site;
2. compare directly Laurent *F*-crystals on the absolute perfect prismatic site with derived representations.

#### Laurent *F*-crystals on perfect site

In this subsection, we investigate the first part of our strategy. The main result is the following.

##### Proposition 3.45

Let *R* be an animated ring in ${\textbf {Nilp}}_{\mathbb {Z}_p,\mathrm ft}^{\le n}$ for some *n*. Then there is an equivalence

##### Proof

As usual, using the Breuil–Kisin prism $(\mathfrak {S},(E))$ and its perfection $(A_{\infty },(E))$, we haveandBy using the same arguments as in the proof of Theorem 2.24, we see that

$${\textrm{Vect}}(\mathfrak {S}^i_{R}[\frac{1}{E}])^{\varphi =1,\simeq }\rightarrow {\textrm{Vect}}(A^i_{\infty ,R}[\frac{1}{E}])^{\varphi =1,\simeq }$$

is fully faithful for every $0\le i\le n+2$. We now want to prove ${\textrm{Vect}}(\mathfrak {S}_{R}[\frac{1}{E}])^{\varphi =1,\simeq }\rightarrow {\textrm{Vect}}(A_{\infty ,R}[\frac{1}{E}])^{\varphi =1,\simeq }$ is moreover essentially surjective.

Note that we have a commutative diagramWe know that $l_1$ is fully faithful and $l_2,l_3$ are essentially surjective (by the same arguments as in the proof of Proposition 3.31). Moreover by [12, Proposition 2.6.12], $l_4$ is an equivalence. Then by Lemma 3.32, we see that $l_1$ is also essentially surjective. Finally by Lemma 2.23, we are done. $\square$

##### Remark 3.46

Proposition 3.45 recovers Proposition 2.24 as finite type $\mathbb {Z}_p$-algebras are discrete Noetherian animated rings over $\mathbb {Z}_p$.

#### Derived representations with finite coefficients

In this subsection, we will relate derived *F*-crystals on the perfect site to derived Galois representations. Let us first recall the construction of derived local Galois deformation functor in [18, Definition 5.4].

##### Construction 3.47

(Unframed derived local Galois deformation functor) Let ${\bar{\rho }}:G_K\rightarrow {\textrm{GL}}_d(k_f)$ be a residual representation. We can define a functor $\mathcal {F}_{K,{\textrm{GL}}_d}:{{\textbf {Art}}}\rightarrow {\textbf {Ani}}$ as

$$ \mathcal {F}_{K,{\textrm{GL}}_d}(R):=\textrm{Map}_{{\textbf {Ani}}}(|G_K|,|{\textrm{GL}}_d(R)|):=\varinjlim _i\textrm{Map}_{{\textbf {Ani}}}(|G_i|,|{\textrm{GL}}_d(R)|) $$

where $G_K=\varprojlim _iG_i$ is the inverse limit of finite quotient groups and $|\cdot |$ means geometric realization of the corresponding simplicial anima, i.e. $|G_i|=\varinjlim _jG_i^j$ and $|{\textrm{GL}}_d(R)|=\varinjlim _j{\textrm{GL}}_d^j(R)=\varinjlim _j\textrm{Map}(\mathcal {O}({\textrm{GL}}_d)^{\otimes j},R))$. Then the unframed derived Galois deformation functor is $\mathcal {F}_{K,{\textrm{GL}}_d,{\bar{\rho }}}:{{\textbf {Art}}_{/k_f}}\rightarrow {\textbf {Ani}}$ is defined as

$$ \mathcal {F}_{K,{\textrm{GL}}_d,{\bar{\rho }}}(R):=\textrm{Fib}_{{\bar{\rho }}}( \mathcal {F}_{K,{\textrm{GL}}_d}(R)\rightarrow \mathcal {F}_{K,{\textrm{GL}}_d}(k_f)) $$

##### Proposition 3.48

Let *R* be a finite Artinian local ring in ${\textbf {Nilp}}_{\mathbb {Z}_p}^{\le n}$ for some *n*. In particular, $\pi _0(R)$ is a discrete finite Artinian local ring. There is an equivalence

##### Proof

Assume *R* is a $\mathbb {Z}/p^a$-algebra for some *a*. We first define an anima ${\textrm{Vect}}(R,G_K)^{\simeq }$ as follows

$$\begin{aligned}  &   {\textrm{Vect}}(R,G_K)^{\simeq }:=\varprojlim _{\Delta _{\le n+1}}({\textrm{Vect}}(R)^{\simeq }\rightrightarrows {\textrm{Vect}}(C(G_K,\mathbb {Z}/p^a)\otimes R)^{\simeq }\begin{array}{c} \rightarrow \\ [-1em] \rightarrow \\ [-1em] \rightarrow \end{array}\\  &   \quad \cdots {\textrm{Vect}}(C(G_K^{n+1},\mathbb {Z}/p^a)\otimes R)^{\simeq }) \end{aligned}$$

where the cosimplicial animated rings $C(G_K^{\bullet },\mathbb {Z}/p^a)\otimes R$ is defined using the trivial $G_K$-action on $\mathbb {Z}/p^a$. We will then compare both  and $\mathcal {F}_{K,{\textrm{GL}}_d}(R)$ with ${\textrm{Vect}}(R,G_K)^{\simeq }$.

Let us start with $\mathcal {F}_{K,{\textrm{GL}}_d}(R)$. Note that any finite projective module over *R* is finite free as *R* is an Artinian local animated ring (see the first paragraph of the proof of [25, Proposition 2.5.3]). So $\pi _0({\textrm{Vect}}(R)^{\simeq })=\pi _0(B{\textrm{GL}}_d(R))=\{*\}$, which is represented by $R^d$. Now we claim ${\textrm{Vect}}(R)^{\simeq }$ is equivalent to $|{\textrm{GL}}_d(R)|=\varinjlim _m{\textrm{GL}}_d^m(R)$.

As $B{\textrm{GL}}_d$ is indeed the sheafification of the functor $|{\textrm{GL}}_d(-)|$, we get a sequence $*\rightarrow |{\textrm{GL}}_d(R)|\rightarrow B{\textrm{GL}}_d(R)$. Note that $\pi _0(|{\textrm{GL}}_d(R)|)=\{*\}$. Then it suffices to compare the mapping space $\textrm{Map}(*,*)$ in both anima. By [18, Lemma 5.2], we know that $\textrm{Map}(*,*)$ in $|{\textrm{GL}}_d(R)|$ is ${\textrm{GL}}_d(R)$. By the homotopy pullback diagramwe see that $\textrm{Map}(*,*)$ in $B{\textrm{GL}}_d(R)$ is also ${\textrm{GL}}_d(R)$. So $|{\textrm{GL}}_d(R)|\simeq {\textrm{Vect}}(R)^{\simeq }$.

As ${\textrm{Vect}}(R)^{\simeq }$ has only one object $R^d$, we can rewrite ${\textrm{Vect}}(R,G_K)^{\simeq }$ as follows

$$\begin{aligned}  &   {\textrm{Vect}}(R,G_K)^{\simeq }:=\varprojlim _{\Delta _{\le n+1}}(\textrm{Free}(R)^{\simeq }\rightrightarrows \textrm{Free}(C(G_K,\mathbb {Z}/p^a)\otimes R)^{\simeq }\begin{array}{c} \rightarrow \\ [-1em] \rightarrow \\ [-1em] \rightarrow \end{array}\\  &   \quad \cdots \textrm{Free}(C(G_K^{n+1},\mathbb {Z}/p^a)\otimes R)^{\simeq }) \end{aligned}$$

where $\textrm{Free}(C(G_K^{\bullet },\mathbb {Z}/p^a)\otimes R)^{\simeq }$ means the full subcategory of ${\textrm{Vect}}(C(G_K^{\bullet },\mathbb {Z}/p^a)\otimes R)^{\simeq }$ spanned by $C(G_K^{\bullet },\mathbb {Z}/p^a)\otimes R^d$. In particular, $\textrm{Free}(C(G_K^{j},\mathbb {Z}/p^a)\otimes R)^{\simeq }\simeq |{\textrm{GL}}_d(C(G_K^{j},\mathbb {Z}/p^a)\otimes R)|$ for each *j*. Then we have

$$ {\textrm{Vect}}(R,G_K)^{\simeq }=\varprojlim _j |{\textrm{GL}}_d(C(G_K^j,\mathbb {Z}/p^a)\otimes R)| $$

By the definition of $|\cdot |$ as geometric realization, we see that

$$ |{\textrm{GL}}_d(C(G_K^j,\mathbb {Z}/p^a)\otimes R|\simeq \varinjlim _m {\textrm{GL}}_d^m(C(G_K^j,\mathbb {Z}/p^a)\otimes R). $$

For each *m*, we also have

$$ {\textrm{GL}}_d^m(C(G_K^j,\mathbb {Z}/p^a)\otimes R)=\varinjlim _i{\textrm{GL}}_d^m(C(G_i^j,\mathbb {Z}/p^a)\otimes R), $$

as ${\textrm{GL}}_d^m$ is of finite type. So we get

$$ {\textrm{Vect}}(R,G_K)^{\simeq }\simeq \varprojlim _j\varinjlim _{m,i}{\textrm{GL}}_d^m(C(G_i^j,\mathbb {Z}/p^a)\otimes R)\simeq \varprojlim _j\varinjlim _{m,i}{\textrm{GL}}_d^m(R^{G^j_i}) $$

where $R^{G_i^j}$ is the product of $G_i^j$-copies of *R*. Note that $G_i^j$ is finite.

On the other hand, for $\mathcal {F}_{K,{\textrm{GL}}_d}(R)$, we have

$$\begin{aligned} \mathcal {F}_{K,{\textrm{GL}}_d}(R)&=\varinjlim _i\textrm{Map}_{{\textbf {Ani}}}(|G_i|,|{\textrm{GL}}_d(R)|)\simeq \varinjlim _i\varprojlim _j|{\textrm{GL}}_d(R)|^{G_i^j}\\&\simeq \varinjlim _i\varprojlim _j\varinjlim _m{\textrm{GL}}_d^m(R^{G_i^j}). \end{aligned}$$

As the inverse limit $\varprojlim _j$ is in fact a finite limit which commutes with filtered colimit $\varinjlim _i$, we then get

$$ {\textrm{Vect}}(R,G_K)^{\simeq }\simeq \mathcal {F}_{K,{\textrm{GL}}_d}(R). $$

Next we come to compare ${\textrm{Vect}}(R,G_K)^{\simeq }$ with . By choosing the Fontaine prism $(A_{\inf },(\xi ))$, we can write  as

$$\begin{aligned}  &   \varprojlim _j{\textrm{Vect}}(A_{\inf }[\frac{1}{\xi }]/p^a\otimes R)^{\varphi =1,\simeq }\rightrightarrows {\textrm{Vect}}(C(G_K,A_{\inf }[\frac{1}{\xi }]/p^a)\otimes R)^{\varphi =1,\simeq }\\  &   \quad \cdots {\textrm{Vect}}(C(G_K^{n+1},A_{\inf }[\frac{1}{\xi }]/p^a)\otimes R)^{\varphi =1,\simeq }. \end{aligned}$$

Then there is a natural base change mapFor each $j\in [0,n+1]$, we claim the base change functor ${\textrm{Vect}}(C(G_K^j,\mathbb {Z}/p^a)\otimes R)^{\simeq }\rightarrow {\textrm{Vect}}(C(G_K^j,A_{\inf }[\frac{1}{\xi }]/p^a)\otimes R)^{\varphi =1,\simeq }$ is fully faithful. This means for every $M\in {\textrm{Vect}}(C(G_K^j,\mathbb {Z}/p^a)\otimes R)^{\simeq }$, we need to prove $M\simeq (M\otimes _{C(G_K^j,\mathbb {Z}/p^a)} C(G_K^j,A_{\inf }[\frac{1}{\xi }]/p^a))^{\varphi =1}$. This follows from the short exact sequence

3.8 $$\begin{aligned} 0\rightarrow C(G_K^j,\mathbb {Z}/p^a)\rightarrow C(G_K^j,A_{\inf }[\frac{1}{\xi }]/p^a)\xrightarrow {\varphi -1}C(G_K^j,A_{\inf }[\frac{1}{\xi }]/p^a)\rightarrow 0. \nonumber \\ \end{aligned}$$

It is easy to see the sequence 3.8 is left exact. To see it is right exact, we first prove $C(G_K^j,\mathcal {O}_C^{\flat })\xrightarrow {\varphi -1}C(G_K^j,\mathcal {O}_C^{\flat })$ is surjective. Let $\varpi$ be a pesudo-uniformiser of $\mathcal {O}_C^{\flat }$. As $C^{\flat }$ is algebraically closed, we see that $\mathcal {O}_C^{\flat }\xrightarrow {\varphi -1}\mathcal {O}_C^{\flat }$ is surjective. Then so is $\mathcal {O}_C^{\flat }/{\varpi ^n}\xrightarrow {\varphi -1}\mathcal {O}_C^{\flat }/{\varpi ^n}$ for each *n*. This implies $C(G_K^j,\mathcal {O}_C^{\flat }/{\varpi ^n})\xrightarrow {\varphi -1}C(G_K^j,\mathcal {O}_C^{\flat }/{\varpi ^n})$ is surjective. Then by the Artin–Schreier sequence ( [34, Tag 0A3J]), we have a short exact sequence

$$ 0\rightarrow \underline{\mathbb {F}_p}(\textrm{Spec}(C(G_K^j,\mathcal {O}_C^{\flat }/{\varpi ^n})))\rightarrow C(G_K^j,\mathcal {O}_C^{\flat }/{\varpi ^n})\xrightarrow {\varphi -1}C(G_K^j,\mathcal {O}_C^{\flat }/{\varpi ^n})\rightarrow 0. $$

Note that $R^1\varprojlim _n \underline{\mathbb {F}_p}(\textrm{Spec}(C(G_K^j,\mathcal {O}_C^{\flat }/{\varpi ^n})))=0$. Then by taking limit with respect to *n*, we get a short exact sequence

$$ 0\rightarrow C(G_K^j,\mathbb {F}_p)\rightarrow C(G_K^j,\mathcal {O}_C^{\flat })\xrightarrow {\varphi -1}C(G_K^j,\mathcal {O}_C^{\flat })\rightarrow 0. $$

So we also have $C(G_K^j,C^{\flat })\xrightarrow {\varphi -1}C(G_K^j,C^{\flat })$ is surjective. Now consider the following exact triangle

$$ C(G_K^j,W(C^{\flat }))\xrightarrow {\varphi -1}C(G_K^j,W(C^{\flat }))\rightarrow N. $$

We have $H^{-1}(N)=C(G_K^j,\mathbb {Z}_p)$ and $H^{-1}(N\otimes ^{\mathbb {L}}_{\mathbb {Z}_p} \mathbb {F}_p)=C(G_K^j,\mathbb {F}_p)$. Since there is an exact sequence

$$ 0\rightarrow H^{-1}(N)/p\rightarrow H^{-1}(N\otimes ^{\mathbb {L}}_{\mathbb {Z}_p}\mathbb {F}_p)\rightarrow H^0(N)[p]\rightarrow 0, $$

we see that $H^0(N)[p]=0$. Now considering the following short exact sequence

$$ 0\rightarrow H^0(N)/p\rightarrow H^0(N\otimes _{\mathbb {Z}_p}^{\mathbb {L}}\mathbb {F}_p)\rightarrow H^1(N)[p]=0\rightarrow 0, $$

we get $H^0(N)/p=H^0(N\otimes _{\mathbb {Z}_p}^{\mathbb {L}}\mathbb {F}_p)=0$. So $H^0(N)\otimes _{\mathbb {Z}_p}^{\mathbb {L}}\mathbb {F}_p=0$. As $H^0(N)$ is derived *p*-complete, we have $H^0(N)=0$ by derived Nakayama lemma. This means $C(G_K^j,W(C^{\flat }))\xrightarrow {\varphi -1}C(G_K^j,W(C^{\flat }))$ is surjective. Then so is $C(G_K^j,W(C^{\flat })/p^n)\xrightarrow {\varphi -1}C(G_K^j,W(C^{\flat })/p^n)$, which finally shows that the sequence 3.8 is exact.

Now we claim that for $j=0$, the base change functor ${\textrm{Vect}}(R)^{\simeq }\rightarrow {\textrm{Vect}}( A_{\inf }[\frac{1}{\xi }]/p^a\otimes R)^{\varphi =1,\simeq }$ is essentially surjective. Note that we have the following commutative diagramWe know that $l_1$ is fully faithful and $l_2,l_3$ are essentially surjective (by the same arguments as in the proof of Proposition 3.31). In fact, $l_2$ is indeed an equivalence. As *R* is finite Artinian, the functor $l_4$ is also an equivalence by [9, Theorem 2.1.27]. By Lemma 3.32, we must have $l_1$ is an equivalence. This implies the equivalenceSo we finally get the equivalence$\square$

#### Derived representations with general coefficients

One direct consequence of Proposition 3.45 and Proposition 3.48 is the following corollary.

##### Corollary 3.49

There is an equivalence of functors $\mathcal {X}_{{\bar{\rho }}}\simeq \mathcal {F}_{K,{\textrm{GL}}_d,{\bar{\rho }}}: {{\textbf {Art}}}_{/k_f}\rightarrow {\textbf {Ani}}$.

Then in order to prove $({\textrm{Lan}}\mathcal {X}_\textrm{EG})^{\#}_{{\bar{\rho }}}\simeq \mathcal {X}_{{\bar{\rho }}}$, it remains to prove the equivalence between $({\textrm{Lan}}\mathcal {X}_\textrm{EG})^{\#}_{{\bar{\rho }}}$ and $\mathcal {F}_{K,{\textrm{GL}}_d,{\bar{\rho }}}$. As a preparation, we recall some results in [38] concerning derived representations with general coefficients.

##### Definition 3.50

[38, Definition 2.4.3] We define the derived prestack of framed ${\textrm{GL}}_d$-valued continuous representations of $G_K$ over $\mathbb {Z}_p$ as

$$ \mathcal {R}_{G_K,{\textrm{GL}}_d}^{c}:{{\textbf {Nilp}}_{\mathbb {Z}_p}}\rightarrow {{\textbf {Ani}}}, \ \ \ A\mapsto \varinjlim _r\textrm{Map}_{{{\textbf {Nilp}}_{\mathbb {Z}_p}^{\Delta }}}(\mathbb {Z}/p^r[{\textrm{GL}}_d^{\bullet }],C_\textrm{cts}(G_K^{\bullet },A)), $$

where $\mathbb {Z}/p^r[{\textrm{GL}}_d^{\bullet }]$ is the coordinate ring.

We can also define the derived prestack $\mathcal {R}_{G_K,{\textrm{GL}}_d/{\textrm{GL}}_d}^{c}$^12^ of unframed ${\textrm{GL}}_d$-valued continuous representations of $G_K$ over $\mathbb {Z}_p$ as the geometric realization of3.9in the $\infty$-category ${\textrm{PreStk}}$.

Still write $G_K=\varprojlim _iG_i$ with $G_i$’s finite quotient groups.

##### Proposition 3.51

[38, Proposition 2.4.7] If *A* is a truncated animated ring in ${{\textbf {Nilp}}_{\mathbb {Z}_p}}$, then

$$ \mathcal {R}_{G_K,{\textrm{GL}}_d}^{c}(A)\simeq \varinjlim _i\mathcal {R}_{G_i,{\textrm{GL}}_d}^{c}(A) $$

In particular, each $\mathcal {R}_{G_i,{\textrm{GL}}_d}^{c}$.^13^ turns out to be well-behaved.

##### Proposition 3.52

( [38, Proposition 2.2.3 and 2.2.11]) $\mathcal {R}_{G_i,{\textrm{GL}}_d}^{c}$ is a derived affine scheme locally almost of finite presentation over $\mathbb {Z}_p$.

Now we are ready to prove our main result.

##### Proposition 3.53

There is an equivalence of functors

$$ ({\textrm{Lan}}\mathcal {X}_\textrm{EG})^{\#}_{{\bar{\rho }}}\simeq \mathcal {F}_{K,{\textrm{GL}}_d,{\bar{\rho }}}. $$

##### Proof

By Lemma 3.54, we have $({\textrm{Lan}}\mathcal {X}_\textrm{EG})^{\#}_{{\bar{\rho }}}\simeq ({\textrm{Lan}}\mathcal {X}_\textrm{EG})_{{\bar{\rho }}}$. So we just need to focus on proving $({\textrm{Lan}}\mathcal {X}_\textrm{EG})_{\bar{\rho }}\simeq \mathcal {F}_{K,{\textrm{GL}}_d,{\bar{\rho }}}$. We will proceed in three steps

1. prove $$({\textrm{Lan}}\mathcal {X}_\textrm{EG})_{{\bar{\rho }}}\simeq ({\textrm{Lan}}^\textrm{cl}\mathcal {R}_{G_K,{\textrm{GL}}_d/{\textrm{GL}}_d}^{c})_{{\bar{\rho }}}$$;
2. prove $$({\textrm{Lan}}^\textrm{cl}\mathcal {R}_{G_K,{\textrm{GL}}_d/{\textrm{GL}}_d}^{c})_{{\bar{\rho }}}\simeq \mathcal {R}_{G_K,{\textrm{GL}}_d/{\textrm{GL}}_d,{\bar{\rho }}}^{c}$$;
3. prove $$\mathcal {R}_{G_K,{\textrm{GL}}_d/{\textrm{GL}}_d,{\bar{\rho }}}^{c}\simeq \mathcal {F}_{K,{\textrm{GL}}_d,{\bar{\rho }}}$$.

**Step 1**: let us begin with the first step. Let $\mathrm{Nilp_{\mathbb {Z}_p,\mathrm ft}}$ denote the full subcategory of $\textrm{Nilp}_{\mathbb {Z}_p}$ spanned by finite type $\mathbb {Z}_p$-algebras. Recall that $\mathcal {X}_\textrm{EG}$ is limit-preserving, or geometrically speaking locally of finite type. Then by [17, Chapter 2, Section 1.6] and [7, Proposition 5.1.2] we can write

$$ \mathcal {X}_\textrm{EG}\simeq \varinjlim _{({\textrm{Spec}}(A)\rightarrow \mathcal {X}_\textrm{EG})\in \textrm{Nilp}_{\mathbb {Z}_p}^{\textrm{op}}\,_{/{\mathcal {X}_{\textrm{EG}}}}}h_A\simeq \varinjlim _{({\textrm{Spec}}(A)\rightarrow \mathcal {X}_{\textrm{EG}})\in \textrm{Nilp}_{\mathbb {Z}_p,\textrm{ft}}^{\textrm{op}}\,_{/{\mathcal {X}_{\textrm{EG}}}}} h_A, $$

where $\textrm{Nilp}_{\mathbb {Z}_p}^\textrm{op}{}_{/{\mathcal {X}_\textrm{EG}}}$, $\textrm{Nilp}_{\mathbb {Z}_p,\mathrm ft}^\textrm{op}{}_{/{\mathcal {X}_\textrm{EG}}}$ are full subcategories of the overcategory $\textrm{PreStk}{}_{/{\mathcal {X}_\textrm{EG}}}$, the prestack $h_A$ is representable by *A* and the colimit is taken in the $\infty$-category $^\textrm{cl}\mathrm PreStk$. Then we have

$$ {\textrm{Lan}}\mathcal {X}_\textrm{EG}\simeq \varinjlim _{\textrm{Nilp}_{\mathbb {Z}_p,\mathrm ft}^\textrm{op}\,_{/{\mathcal {X}_\textrm{EG}}}} h_A $$

where the colimit is taken in the $\infty$-category ${\textrm{PreStk}}$ of derived prestacks, as the left Kan extension along $\textrm{Nilp}_{\mathbb {Z}_p}\rightarrow {\textbf {Nilp}}_{\mathbb {Z}_p}$ sends $h_A$ to $h_A$. Now the functor $({\textrm{Lan}}\mathcal {X}_\textrm{EG})_{{\bar{\rho }}}:{\textbf {Art}}_{/k_f}\rightarrow {\textbf {Ani}}$ is defined as

3.10 $$\begin{aligned} ({\textrm{Lan}}\mathcal {X}_\textrm{EG})_{{\bar{\rho }}}(R\rightarrow k_f)  &   :={\textrm{Lan}}\mathcal {X}_\textrm{EG}(R)\times _{\mathcal {X}_\textrm{EG}(k_f)}*_{{\bar{\rho }}}\nonumber \\  &   \simeq (\varinjlim _{\textrm{Nilp}_{\mathbb {Z}_p,\mathrm ft}^\textrm{op}{}_{/{\mathcal {X}_\textrm{EG}}}}h_A(R))\times _{\mathcal {X}_\textrm{EG}(k_f)}*_{{\bar{\rho }}}, \end{aligned}$$

where $*_{{\bar{\rho }}}$ is the point corresponding to ${\bar{\rho }}$.

Let *H* be the set of connected components of $\mathcal {X}_\textrm{EG}(k_f)$ and choose a representative $*_{\alpha }$ from each connected component. Then we can write $\mathcal {X}_\textrm{EG}(k_f)\simeq \bigsqcup _{\alpha \in H}[*_{\alpha }/{\textrm{Aut}(*_{\alpha })}]$. For any ${\bar{\rho }}_A:h_A\rightarrow \mathcal {X}_\textrm{EG}$ and $(R\rightarrow k_f)\in {\textbf {Art}}_{/k_f}$, we then can write

$$ h_A(R)\simeq \bigsqcup _{\alpha \in H}h_{A,\alpha }(R), $$

where $h_{A,\alpha }(R)$ is the preimage of $[*_{\alpha }/{\textrm{Aut}(*_{\alpha })}]$. In particular, there is $h_{A,{\bar{\rho }}}(R)$ corresponding to the fixed residual representation ${\bar{\rho }}$. As a result, we see that

$$\begin{aligned}  &   ({\textrm{Lan}}\mathcal {X}_\textrm{EG})_{{\bar{\rho }}}(R\rightarrow k_f)\simeq (\bigsqcup _{\alpha \in H}\varinjlim _{\textrm{Nilp}_{\mathbb {Z}_p,\mathrm ft}^\textrm{op}{}_{/{\mathcal {X}_\textrm{EG}}}}h_{A,\alpha }(R))\times _{\mathcal {X}_\textrm{EG}(k_f)}*_{{\bar{\rho }}}\\  &   \quad \simeq (\varinjlim _{\textrm{Nilp}_{\mathbb {Z}_p,\mathrm ft}^\textrm{op}{}_{/{\mathcal {X}_\textrm{EG}}}}h_{A,{\bar{\rho }}}(R))\times _{\mathcal {X}_\textrm{EG}(k_f)}*_{{\bar{\rho }}}. \end{aligned}$$

Now we want to further study each $h_{A,{\bar{\rho }}}(R)$. We first look at $h_{A,{\bar{\rho }}}(k_f)$. Note that each element in $h_{A,{\bar{\rho }}}(k_f)$ determines a maximal ideal of *A* as $k_f$ is a finite field. Let $T_{{\bar{\rho }}_A}$ denote the set of maximal ideals appearing in this way and we can write $h_{A,{\bar{\rho }}}(k_f)=\bigsqcup _{m_\beta \in T_{{\bar{\rho }}_A}}h_{A,m_\beta }(k_f)$, such that all elements in $h_{A,m_\beta }(k_f)$ induce the same maximal ideal $m_{\beta }$. For each $m_{\beta }$, let $A_{(m_{\beta })}$ be the localization of *A* at the maximal ideal $m_{\beta }$ and ${\hat{A}}_{(m_{\beta })}$ be its completion. Then we have $h_{A,m_\beta }(k_f)=h_{A_{(m_{\beta })},m_\beta }(k_f)=h_{{\hat{A}}_{(m_{\beta })},m_\beta }(k_f)$.

Now for $(R\rightarrow k_f)\in {\textbf {Art}}_{/k_f}$, we have $h_{A,\bar{\rho }}(R)\simeq \bigsqcup _{m_{\beta }\in T_{{\bar{\rho }}_A}}h_{A,m_{\beta }}(R)$, which is induced by the stratification $h_{A,{\bar{\rho }}}(k_f)=\bigsqcup _{m_\beta \in T_{{\bar{\rho }}_A}}h_{A,m_\beta }(k_f)$ and the natural map $h_{A,{\bar{\rho }}}(R)\rightarrow h_{A,{\bar{\rho }}}(k_f)$. Now we claim that $h_{A,m_{\beta }}(R)\simeq h_{A_{(m_{\beta })},m_{\beta }}(R)\simeq h_{{\hat{A}}_{(m_{\beta })},m_{\beta }}(R)$. Let $\gamma :A\rightarrow k_f$ be a map in $h_{A,m_{\beta }}(k_f)$. Then we can define a functor $h_{A,\gamma }:{{\textbf {Art}}}_{/k_f}\rightarrow {\textbf {Ani}}$ by

$$ h_{A,\gamma }(R):=\textrm{Fib}_{\gamma }(h_A(R)\rightarrow h_A(k_f)). $$

Similarly, we can define $h_{A_{(m_{\beta })},\gamma }$ and $h_{{\hat{A}}_{(m_{\beta })},\gamma }$. By [25, Remark 6.2.9], if we want to prove $h_{{\hat{A}}_{(m_{\beta })},\gamma }\simeq h_{A_{(m_{\beta })},\gamma }\simeq h_{A,\gamma }$, we just need to compare their tangent complexes. By [25, Proposition 6.2.10 (2)], the tangent complexes are the duals of $L_{{\hat{A}}_{(m_{\beta })}/\mathbb {Z}}\otimes ^{\mathbb {L}}_{{\hat{A}}_{(m_{\beta })}}k_f$, $L_{ A_{(m_{\beta })}/\mathbb {Z}}\otimes ^{\mathbb {L}}_{A_{(m_{\beta })}}k_f$, $L_{A/\mathbb {Z}}\otimes ^{\mathbb {L}}_{A}k_f$ respectively. Now consider the natural maps $\mathbb {Z}\rightarrow A\rightarrow A_{(m_{\beta })}$. There is an associated exact triangle

$$ L_{A/\mathbb {Z}}\otimes ^{\mathbb {L}}_AA_{(m_{\beta })}\rightarrow L_{A_{(m_{\beta })}/\mathbb {Z}}\rightarrow L_{A_{(m_{\beta })}/A}, $$

which induces an exact triangle

$$ L_{A/\mathbb {Z}}\otimes ^{\mathbb {L}}_Ak_f\rightarrow L_{A_{(m_{\beta })}/\mathbb {Z}}\otimes ^{\mathbb {L}}_{A_{(m_{\beta })}}k_f\rightarrow L_{A_{(m_{\beta })}/A}\otimes ^{\mathbb {L}}_{A_{(m_{\beta })}}k_f. $$

Now we consider the exact triangle induced by $A\rightarrow A_{(m_{\beta })}\rightarrow k_f$

$$ L_{A_{(m_{\beta })}/A}\otimes ^{\mathbb {L}}_{A_{(m_{\beta })}}k_f\rightarrow L_{k_f/A}\rightarrow L_{k_f/A_{(m_{\beta })}}. $$

As $A\rightarrow A_{(m_{\beta })}$ is flat, we then have $L_{k_f/A}\otimes ^{\mathbb {L}}_AA_{(m_{\beta })}\simeq L_{k_f\otimes _AA_{(m_{\beta })}/A_{(m_{\beta })}}$. Note that $k_f\otimes _AA_{(m_{\beta })}\simeq k_f$ and $L_{k_f/A}\otimes ^{\mathbb {L}}_AA_{(m_{\beta })}\simeq L_{k_f/A}\otimes ^{\mathbb {L}}_{k_f}k_f\otimes ^{\mathbb {L}}_AA_{(m_{\beta })}\simeq L_{k_f/A}$. Then we have $L_{k_f/A}\simeq L_{k_f/A_{(m_{\beta })}}$, which implies $L_{A_{(m_{\beta })}/A}\otimes ^{\mathbb {L}}_{A_{(m_{\beta })}}k_f\simeq 0$. So we get $L_{A/\mathbb {Z}}\otimes ^{\mathbb {L}}_Ak_f\simeq L_{A_{(m_{\beta })}/\mathbb {Z}}\otimes ^{\mathbb {L}}_{A_{(m_{\beta })}}k_f$. By using the facts that $A_{(m_{\beta })}\rightarrow {\hat{A}}_{(m_{\beta })}$ is flat and $k_f\otimes _{A_{(m_{\beta })}}{\hat{A}}_{(m_{\beta })}\simeq k_f$, we can run the same argument to deduce $L_{ A_{(m_{\beta })}/\mathbb {Z}}\otimes ^{\mathbb {L}}_{A_{(m_{\beta })}}k_f\simeq L_{{\hat{A}}_{(m_{\beta })}/\mathbb {Z}}\otimes ^{\mathbb {L}}_{{\hat{A}}_{(m_{\beta })}}k_f$. So we have proved $h_{{\hat{A}}_{(m_{\beta })},\gamma }\simeq h_{A_{(m_{\beta })},\gamma }\simeq h_{A,\gamma }$. This implies $h_{{\hat{A}}_{(m_{\beta })},m_{\beta }}(R)\simeq h_{A_{(m_{\beta })},m_{\beta }}(R)\simeq h_{A,m_{\beta }}(R)$.

Now we have a decomposition

$$ h_{A,{\bar{\rho }}}(R)\simeq \bigsqcup _{m_{\beta }\in T_{{\bar{\rho }}_A}}h_{{\hat{A}}_{(m_{\beta })},m_{\beta }}(R). $$

Note that ${\hat{A}}_{(m_{\beta })}$ is a local ring. So we can also write

$$ h_{A,{\bar{\rho }}}(R)\simeq \bigsqcup _{m_{\beta }\in T_{\bar{\rho }_A}}h_{{\hat{A}}_{(m_{\beta })},{\bar{\rho }}}(R). $$

Let ${\hat{A}}_{(m_{\beta }),n}$ be the quotient ${\hat{A}}_{(m_{\beta })}/m_{\beta }^n$ for any $n\ge 1$. We then have

$$ h_{{\hat{A}}_{(m_{\beta })},{\bar{\rho }}}(R)\simeq \varinjlim _nh_{{\hat{A}}_{(m_{\beta }),n},{\bar{\rho }}}(R) \ \ \text {and} \ \ h_{A,\bar{\rho }}(R)\simeq \bigsqcup _{m_{\beta }\in T_{\bar{\rho }_A}}\varinjlim _nh_{{\hat{A}}_{(m_{\beta }),n},{\bar{\rho }}}(R). $$

Now we want to investigate $\varinjlim _{\textrm{Nilp}_{\mathbb {Z}_p,\mathrm ft}^\textrm{op}{}_{/{\mathcal {X}_\textrm{EG}}}}h_{A,{\bar{\rho }}}(R)$. Let $\bar{\rho }_A,{\bar{\rho }}_B\in \textrm{Nilp}_{\mathbb {Z}_p,\mathrm ft}^\textrm{op}{}_{/{\mathcal {X}_\textrm{EG}}}$ be two elements in the overcategory and $f:{\bar{\rho }}_A\rightarrow {\bar{\rho }}_B$ be a 1-morphism. Then we have an induced map $f: h_{A,{\bar{\rho }}}(k_f)\rightarrow h_{B,{\bar{\rho }}}(k_f)$. In particular, this gives a map of sets of maximal ideals $f^{-1}: T_{{\bar{\rho }}_A}\rightarrow T_{{\bar{\rho }}_B}$. For each $m_{\beta ,A}\in T_{{\bar{\rho }}_A}$, we have induced maps $f_{m_{\beta ,A},n}:{\hat{B}}_{f^{-1}(m_{\beta ,A}),n}\rightarrow {\hat{A}}_{m_{\beta ,A},n}$. So the map $f:h_{A,{\bar{\rho }}}(R)\rightarrow h_{B,{\bar{\rho }}}(R)$ is actually induced by each $f_{m_{\beta ,A},n}$, i.e.

3.11 $$\begin{aligned} f=\bigsqcup _{m_{\beta ,A}\in T_{{\bar{\rho }}_A}}\varinjlim _n f_{m_{\beta ,A},n}:\bigsqcup _{m_{\beta ,A}\in T_{{\bar{\rho }}_A}}\varinjlim _nh_{{\hat{A}}_{(m_{\beta ,A}),n},{\bar{\rho }}}(R)\rightarrow \bigsqcup _{m_{\beta ,B}\in T_{{\bar{\rho }}_B}}\varinjlim _nh_{{\hat{B}}_{(m_{\beta ,B}),n},\bar{\rho }}(R). \nonumber \\ \end{aligned}$$

Let $\textrm{Art}^\textrm{op}_{\mathbb {Z}_p}{}_{/{\mathcal {X}_\textrm{EG}}}$ be the subcategory of $\textrm{Nilp}_{\mathbb {Z}_p,\mathrm ft}^\textrm{op}{}_{/{\mathcal {X}_\textrm{EG}}}$ spanned by finite Artinian local $\mathbb {Z}_p$-algebras. So we have a natural map

3.12 $$\begin{aligned} \iota :\varinjlim _{({\textrm{Spec}}(C)\rightarrow \mathcal {X}_\textrm{EG})\in \textrm{Art}^\textrm{op}_{\mathbb {Z}_p}{}_{/{\mathcal {X}_\textrm{EG}}}}h_{C,{\bar{\rho }}}(R)\rightarrow \varinjlim _{({\textrm{Spec}}(A)\rightarrow \mathcal {X}_\textrm{EG})\in \textrm{Nilp}_{\mathbb {Z}_p,\mathrm ft}^\textrm{op}{}_{/{\mathcal {X}_\textrm{EG}}}}h_{A,{\bar{\rho }}}(R).\nonumber \\ \end{aligned}$$

We claim that $\iota$ is an equivalence. We prove this by constructing its quasi-inverse. For any ${\bar{\rho }}_A:h_A\rightarrow \mathcal {X}_\textrm{EG}$ in $\textrm{Nilp}_{\mathbb {Z}_p,\mathrm ft}^\textrm{op}{}_{/{\mathcal {X}_\textrm{EG}}}$, we know that $h_{A,{\bar{\rho }}}(R)\simeq \bigsqcup _{m_{\beta }\in T_{{\bar{\rho }}_A}}\varinjlim _nh_{{\hat{A}}_{(m_{\beta }),n},{\bar{\rho }}}(R)$. Moreover as each ${\hat{A}}_{(m_{\beta }),n}$ is a finite Artinian local $\mathbb {Z}_p$-algebra, there is a natural map

$$ \bigsqcup _{m_{\beta }\in T_{{\bar{\rho }}_A}}\varinjlim _n h_{{\hat{A}}{_{(m_{\beta }),n}},{\bar{\rho }}}(R)\rightarrow \varinjlim _{({\textrm{Spec}}(C)\rightarrow \mathcal {X}_{\textrm{EG}})\in \textrm{Art}^{\textrm{op}}_{\mathbb {Z}_p}\,_{/{\mathcal {X}_{\textrm{EG}}}}}h_{C,{\bar{\rho }}}(R) $$

which is unique up to a contractible space of choices. Then we get a natural map

$$i_A: h_{A,{\bar{\rho }}}(R)\rightarrow \varinjlim _{({\textrm{Spec}}(C)\rightarrow \mathcal {X}_\textrm{EG})\in \textrm{Art}^\textrm{op}_{\mathbb {Z}_p}\,_{/{\mathcal {X}_\textrm{EG}}}}h_{C,{\bar{\rho }}}(R).$$

By 3.11, this fits into a well-defined map

$$ {\tilde{\iota }}:\varinjlim _{({\textrm{Spec}}(A)\rightarrow \mathcal {X}_{\textrm{EG}})\in \textrm{Nilp}_{\mathbb {Z}_p,\mathrm ft}^\textrm{op}\,_{/{\mathcal {X}_{\textrm{EG}}}}}h_{A,{\bar{\rho }}}(R)\rightarrow \varinjlim _{({\textrm{Spec}}(C) \rightarrow \mathcal {X}_{\textrm{EG}})\in \textrm{Art}^{\textrm{op}}_{\mathbb {Z}_p}\,_{/{\mathcal {X}_{\textrm{EG}}}}}h_{C,{\bar{\rho }}}(R). $$

It is easy to see that ${\tilde{\iota }}\circ \iota \simeq id$ by the construction of ${\tilde{\iota }}$. Now we consider the composite $\iota \circ {\tilde{\iota }}$. Let $j_A$ denote the natural map $h_{A,{\bar{\rho }}}(R)\rightarrow \varinjlim _{({\textrm{Spec}}(A)\rightarrow \mathcal {X}_\textrm{EG})\in \textrm{Nilp}_{\mathbb {Z}_p,\mathrm ft}^\textrm{op}{}_{/{\mathcal {X}_\textrm{EG}}}}h_{A,{\bar{\rho }}}(R)$. Then we need to see that $\iota \circ {\tilde{\iota }}\circ j_A\simeq j_A$ functorially. Note that ${\tilde{\iota }}\circ j_A$ is just $\bigsqcup _{m_{\beta }\in T_{{\bar{\rho }}_A}}\varinjlim _n i_{{\hat{A}}_{(m_{\beta }),n}}$. Then we see that $\iota \circ {\tilde{\iota }}\circ j_A$ is just $\bigsqcup _{m_{\beta }\in T_{{\bar{\rho }}_A}}\varinjlim _n j_{{\hat{A}}_{(m_{\beta }),n}}$, which is the same as $j_A$ up to a contractible space of choices. We then obtain $\iota \circ {\tilde{\iota }}\simeq id$. So the natural map $\iota$ is an equivalence.

Now go back to the goal of Step 1. As $^\textrm{cl}\mathcal {R}_{G_K,{\textrm{GL}}_d/{\textrm{GL}}_d}^{c}$ is also limit preserving, for any $(R\rightarrow k_f)\in {\textbf {Art}}_{/k_f}$, we also have

3.13 $$\begin{aligned}  &   ({\textrm{Lan}}^\textrm{cl}\mathcal {R}_{G_K,{\textrm{GL}}_d/{\textrm{GL}}_d}^{c})_{{\bar{\rho }}}(R\rightarrow k_f)\nonumber \\  &   \quad =(\varinjlim _{\textrm{Art}^\textrm{op}_{\mathbb {Z}_p}{}_{/{^\textrm{cl}\mathcal {R}_{G_K,{\textrm{GL}}_d/{\textrm{GL}}_d}^{c}}}}h_{C,{\bar{\rho }}}(R))\times _{^\textrm{cl}\mathcal {R}_{G_K,{\textrm{GL}}_d/{\textrm{GL}}_d}^{c}(k_f)}*_{{\bar{\rho }}} \end{aligned}$$

As for finite coefficient rings, the étale $(\varphi ,\Gamma )$-modules are the same as Galois representations by [9, Theorem 2.3.1], we have

3.14 $$\begin{aligned} \textrm{Art}^\textrm{op}_{\mathbb {Z}_p}{}_{/{^\textrm{cl}\mathcal {R}^{c}_{G_K,{\textrm{GL}}_d/{\textrm{GL}}_d}}}\simeq \textrm{Art}^\textrm{op}_{\mathbb {Z}_p}{}_{/{\mathcal {X}_\textrm{EG}}} \end{aligned}$$

by [38, Lemma 2.4.4]. By combining equivalences 3.14 and 3.12, we see that the formulas 3.10 and 3.13 are the same. So we are done with Step 1.

**Step 2**: now we carry out the second step. We first claim that $({\textrm{Lan}}^\textrm{cl}\mathcal {R}_{G_K,{\textrm{GL}}_d}^{c})_{\bar{\rho }}\simeq \mathcal {R}_{G_K,{\textrm{GL}}_d,{\bar{\rho }}}^{c}$. By Propositions 3.51 and 3.52, we see that $^\textrm{cl}\mathcal {R}_{G_K,{\textrm{GL}}_d}^{c}\simeq \varinjlim _ih_{R_i}$, where ${\textrm{Spec}}(R_i)={^\textrm{cl}\mathcal {R}_{G_i,{\textrm{GL}}_d}^{c}}$ and $R_i$ is a finite type $\mathbb {Z}_p$-algebra for each *i*. So ${\textrm{Lan}}^\textrm{cl}\mathcal {R}_{G_K,{\textrm{GL}}_d}^{c}\simeq \varinjlim _ih_{R_i}$ where the colimit is taken in ${\textrm{PreStk}}$. For any $(R\rightarrow k_f)\in {\textbf {Art}}_{/k_f}$, we then have

$$ ({\textrm{Lan}}^\textrm{cl}\mathcal {R}_{G_K,{\textrm{GL}}_d}^{c})_{{\bar{\rho }}}(R\rightarrow k_f)\simeq \varinjlim _ih_{R_i}(R)\times _{\varinjlim _ih_{R_i}(k_f)}*_{\bar{\rho }}\simeq \varinjlim _ih_{R_i,{\bar{\rho }}}(R). $$

By the same argument as in Step 1, we have $h_{R_i,{\bar{\rho }}}(R)\simeq h_{{\hat{R}}_{i,(m_i)},{\bar{\rho }}}(R)$ where ${\hat{R}}_{i,(m_i)}$ is the completion of the localization of $R_i$ at the maximal ideal determined by ${\bar{\rho }}$. The functor $h_{{\hat{R}}_{i,(m_i)},{\bar{\rho }}}$ is then pro-corepresentable by $\{{\hat{R}}_{i,(m_i),n}:={\hat{R}}_{i,(m_i)}/m_i^n\}_n$. So we get the functor $({\textrm{Lan}}^\textrm{cl}\mathcal {R}_{G_K,{\textrm{GL}}_d}^{c})_{{\bar{\rho }}}$ is pro-corepresentable by $\{{\hat{R}}_{i,(m_i),n}\}_{i,n}$. In particular, $({\textrm{Lan}}^\textrm{cl}\mathcal {R}_{G_K,{\textrm{GL}}_d}^{c})_{{\bar{\rho }}}$ is the left Kan extension of $\varinjlim _{i,n}h_{{\hat{R}}_{i,(m_i),n}}: \textrm{Art}_{/k_f}\rightarrow {\textbf {Ani}}$ along the inclusion $\textrm{Art}_{/k_f}\rightarrow {\textbf {Art}}_{/k_f}$. Note that $\varinjlim _{i,n}h_{{\hat{R}}_{i,(m_i),n}}: \textrm{Art}_{/k_f}\rightarrow {\textbf {Ani}}$ is just the classical framed deformation functor by [38, Lemma 2.4.4], which is then equivalent to $^\textrm{cl}\mathcal {F}_{K,{\textrm{GL}}_d,{\bar{\rho }}}^{\square }$ (for the definition of $\mathcal {F}_{K,{\textrm{GL}}_d,{\bar{\rho }}}^{\square }$, see [18, Definition 5.4(i)]). By Theorem 3.1 and [18, Lemma 7.5], we see that $\mathcal {F}_{K,{\textrm{GL}}_d,{\bar{\rho }}}^{\square }$ is also the left Kan extension of $^\textrm{cl}\mathcal {F}_{K,{\textrm{GL}}_d,{\bar{\rho }}}^{\square }$ along the inclusion $\textrm{Art}_{/k_f}\rightarrow {\textbf {Art}}_{/k_f}$. This implies we have $({\textrm{Lan}}^\textrm{cl}\mathcal {R}_{G_K,{\textrm{GL}}_d}^{c})_{{\bar{\rho }}}$ is equivalent to $\mathcal {F}_{K,{\textrm{GL}}_d,{\bar{\rho }}}^{\square }$. The latter by definition is just $\mathcal {R}_{G_K,{\textrm{GL}}_d,{\bar{\rho }}}^{c}$ (cf. [38, Remark 2.2.2]). By Lemma 3.54 and (the same proof of ) Lemma 3.43, we have $({\textrm{Lan}}^\textrm{cl}\mathcal {R}_{G_K,{\textrm{GL}}_d}^{c})^{\#,\textrm{nil}}\simeq \mathcal {R}_{G_K,{\textrm{GL}}_d}^{c,\textrm{nil}}$.

Now we are going to prove $({\textrm{Lan}}^\textrm{cl}\mathcal {R}_{G_K,{\textrm{GL}}_d/{\textrm{GL}}_d}^{c})_{{\bar{\rho }}}\simeq \mathcal {R}_{G_K,{\textrm{GL}}_d/{\textrm{GL}}_d,{\bar{\rho }}}^{c}$. By the fact that left Kan extension commutes with colimits and the definition of $\mathcal {R}_{G_K,{\textrm{GL}}_d/{\textrm{GL}}_d}^{c}$ as the geometric realization of the following simplicial prestacks3.15It suffices to prove for any $(R\rightarrow k_f)\in {\textbf {Art}}_{/k_f}$, we have $({\textrm{Lan}}^{{\textrm{cl}}}\mathcal {R}_{G_K,{\textrm{GL}}_d}^{c})(R)\simeq \mathcal {R}_{G_K,{\textrm{GL}}_d}^{c}(R)$. But this follows from $({\textrm{Lan}}^{{\textrm{cl}}}\mathcal {R}_{G_K,{\textrm{GL}}_d}^{c})_{{\bar{\rho }}}\simeq \mathcal {R}_{G_K,{\textrm{GL}}_d,\bar{\rho }}^{c}$ and the same arguments in the first two paragraphs of Lemma 3.54.

**Step 3**. Now it remains to prove the third step: $\mathcal {R}_{G_K,{\textrm{GL}}_d/{\textrm{GL}}_d,{\bar{\rho }}}^{c}\simeq \mathcal {F}_{K,{\textrm{GL}}_d,{\bar{\rho }}}$. This follows directly from the definition of both sides. More precisely, the framed deformation functor $\mathcal {F}^{\square }_{K,{\textrm{GL}}_d}$ (cf. [18, Definition 5.4(i)]) is equivalent to the framed deformation functor $\mathcal {R}^{c}_{G_K,{\textrm{GL}}_d}$ by [38, Remark 2.2.2]. By [18, Definition 5.4(i)], for any animated ring *R*, we have the pullback diagramThe base change of the simplicial anima ${\textrm{GL}}_d^{\bullet }(R)$ along $\mathcal {F}_{K,{\textrm{GL}}_d}(R)\rightarrow |{\textrm{GL}}_d(R)|$ gives a simplial anima, which is the same as the simplicial anima in [38, Definition 2.2.14]. The homotopy colimit of this simplicial anima is just $\mathcal {F}_{K,{\textrm{GL}}_d}(R)$ by the fact that the colimit in the $\infty$-topos ${\textbf {Ani}}$ is universal. This shows $\mathcal {F}_{K,{\textrm{GL}}_d}(R)$ is equivalent to $\mathcal {R}^{c}_{G_K,{\textrm{GL}}_d/{\textrm{GL}}_d}(R)$ (see also [38, Remark 2.2.15]. $\square$

The following lemma has been used above and enables us to deal with sheafification.

##### Lemma 3.54

Let $\mathcal {Y}$ be a derived prestack which satisifies the étale sheaf property when restricted to ${\textbf {Nilp}}_{\mathbb {Z}_p}^{<\infty }$. Assume $\mathcal {Y}$ admits a deformation theory and for all points $\bar{\rho }:{\textrm{Spec}}(k_f)\rightarrow \mathcal {Y}$ with $k_f$ a finite field, the natural map $({\textrm{Lan}}^\textrm{cl}\mathcal {Y})_{{\bar{\rho }}}\rightarrow \mathcal {Y}_{{\bar{\rho }}}$ is an equivalence. Then we have $({\textrm{Lan}}^{\textrm{cl}}\mathcal {Y})^{\#}_{{\bar{\rho }}}\simeq \mathcal {Y}_{{\bar{\rho }}}$ for all $\bar{ \rho }$.

##### Proof

Write $\mathcal {Y}(k_f)$ as $\bigsqcup _{\alpha \in H}[*_{\alpha }/(\textrm{Aut}(*_{\alpha })]$ where *H* is the set of connected components of $\mathcal {Y}(k_f)$. By the assumption, for any $(R\rightarrow k_f)\in {{{\textbf {Art}}}_{/k_f}}$, we have $({\textrm{Lan}}^\textrm{cl}\mathcal {Y})_{*_{\alpha }}(R\rightarrow k_f)\simeq \mathcal {Y}_{*_\alpha }(R\rightarrow k_f)$. Consider the following pullback diagramNote that $[*_{\alpha }/(\textrm{Aut}(*_{\alpha })]$ is the geometric realization (i.e. the homotopy colimit) of the simplicial anima $(*_{\alpha })^{\bullet }$ corresponding to the automorphism group $\textrm{Aut}(*_{\alpha })$. We write $[*_{\alpha }/(\textrm{Aut}(*_{\alpha })]=\varinjlim \textrm{Aut}(*_{\alpha })^{\bullet }$. Base changing along $\mathcal {Y}_{*\alpha }(R)\rightarrow [*_{\alpha }/(\textrm{Aut}(*_{\alpha })]$, we get another simplicial anima $\mathcal {Y}_{*_\alpha }(R)^{\bullet }$. In particular, $\mathcal {Y}_{*_\alpha }(R)^{0}=\mathcal {Y}_{*_\alpha }(R\rightarrow k_f)$. As the colimit in $\infty$-topos ${\textbf {Ani}}$ is universal, i.e. pullback preserves colimits, we have $\mathcal {Y}_{*_{\alpha }}(R)\simeq \varinjlim \mathcal {Y}_{*\alpha }(R)^{\bullet }$. The same is true for ${\textrm{Lan}}^\textrm{cl}\mathcal {Y}$, i.e. $({\textrm{Lan}}^\textrm{cl}\mathcal {Y})_{*_{\alpha }}(R)\simeq \varinjlim ({\textrm{Lan}}^\textrm{cl}\mathcal {Y})_{*_{\alpha }}(R)^{\bullet }$. By our assumption $({\textrm{Lan}}^\textrm{cl}\mathcal {Y})_{*_{\alpha }}\simeq \mathcal {Y}_{*_{\alpha }}$ and $({\textrm{Lan}}^\textrm{cl}\mathcal {Y})(k_f)\simeq \mathcal {Y}(k_f)$, we get a natural equivalence of simplicial anima $({\textrm{Lan}}^\textrm{cl}\mathcal {Y})_{*_{\alpha }}(R)^{\bullet }\simeq \mathcal {Y}_{*\alpha }(R)^{\bullet }$ induced by the actions of $\textrm{Aut}(*_{\alpha })$ on $({\textrm{Lan}}^\textrm{cl}\mathcal {Y})_{*_\alpha }(R\rightarrow k_f)$ and $\mathcal {Y}_{*_\alpha }(R\rightarrow k_f)$ respectively (in fact, the action of $\textrm{Aut}(*_{\alpha })$ on the former is just the base change of the action on the latter along the natural map $({\textrm{Lan}}^\textrm{cl}\mathcal {Y})_{*_\alpha }(R)\rightarrow \mathcal {Y}_{*\alpha }(R)$). In particular, by taking the geoemtric realization of the two simplicial animas, we get $({\textrm{Lan}}^\textrm{cl})_{*_\alpha }\mathcal {Y}_{*_\alpha }(R)\simeq \mathcal {Y}(R)$ for any finite Artinian local ring with finite residue field. Taking the union over *H*, we get ${\textrm{Lan}}^\textrm{cl}\mathcal {Y}(R)\simeq \mathcal {Y}(R)$.

Now consider any étale map $R_1\rightarrow R_2$ with $R_1$ an Artinian local ring with finite residue field $k_1$. Then $\pi _0(R_2)$ is also an Artinian ring and $\pi _i(R_2)=\pi _0(R_2)\otimes _{\pi _0(R_1)}\pi _i(R_1)$ is a finite $\pi _0(R_2)$-module for each *i*. By definition, we know $R_2$ is also Artinian. In particular, $R_2$ can be written uniquely as a finite product $\prod _jR_{2j}$ of Artinian local rings with finite residue fields (see [25, Section 6.2]). As $\mathcal {Y}$ is an étale stack when restricted to truncated animated rings, we have $\mathcal {Y}(R_2)=\prod _j\mathcal {Y}(R_{2j})$. We claim that this is also true for ${\textrm{Lan}}^\textrm{cl}\mathcal {Y}$. In fact, as $^\textrm{cl}\mathcal {Y}$ is an étale stack, we have $^\textrm{cl}\mathcal {Y}:\textrm{Nilp}_{\mathbb {Z}_p}\rightarrow {\textbf {Ani}}$ preserves finite products. This is equivalent to that $^\textrm{cl}\mathcal {Y}$ can be written as a sifted colimit of representable sheaves, i.e. $^\textrm{cl}\mathcal {Y}\simeq \varinjlim _ih_{A_i}$. As left Kan extension preserves representable sheaves, we have ${\textrm{Lan}}^\textrm{cl}\mathcal {Y}\simeq \varinjlim _ih_{A_i}$ where the colimit is taken in ${\textrm{PreStk}}$. Again, by using that a functor preserving finite products is equivalent to that it is a sifted colimit of representables, we conclude ${\textrm{Lan}}^\textrm{cl}\mathcal {Y}$ preserves finite products. Hence we have ${\textrm{Lan}}^\textrm{cl}\mathcal {Y}(R_2)\simeq \mathcal {Y}(R_2)$.

Now by the description of the sheafification functor [26, Remark 6.2.2.12] (and the proof of [26, Proposition 6.2.2.7]), we have $({\textrm{Lan}}^\textrm{cl}\mathcal {Y})^{\#}(R)\simeq ({\textrm{Lan}}^\textrm{cl}\mathcal {Y})(R)$ for any Artinian local rings *R* with finite residue fields as the sheafification process only involves étale coverings of such rings (see also [17, Chapter 2, Section 2.5.2]). In particular, this means $({\textrm{Lan}}^\textrm{cl}\mathcal {Y})^{\#}_{{\bar{\rho }}}\simeq \mathcal {Y}_{{\bar{\rho }}}$. $\square$

Finally, we can prove Theorem 3.3.

##### Proof of Theorem 3.3

Just combine Proposition 3.45, Proposition 3.48, Proposition 3.53, Lemma 3.54 and Lemma 3.43.

### Derived étale $(\varphi ,\Gamma )$-modules

Throughout this section, we assume *K* is absolutely unramified. We will give an ad-hoc definition of derived étale $(\varphi ,\Gamma )$-modules and prove they are equivalent to derived Laurent *F*-crystals.

Recall there is the cyclotomic prism $({\bar{A}}_K^+,([p]_q)$ (cf. Definition 2.17) in , which is equipped with compatible $\varphi$-action and $\Gamma$-action. For each $n\ge 0$, we define ${\bar{A}}_K^{+,n}/p^a:=C(\Gamma ^n,\bar{A}_K^+/p^a)$. In particular, we get a natural cosimplicial ring $\bar{A}_K^{+,\bullet }/p^a$ by using the $\Gamma$-action on $\bar{A}_K^{+}/p^a$.

#### Definition 3.55

Let *R* be a truncated animated ring which is of finite type over $\mathbb {Z}/p^a$. We define the $\infty$-category ${\textrm{Mod}}^{\varphi ,\Gamma }(A_{K,R})$ of étale $(\varphi ,\Gamma )$-modules with coefficient in *R* to be the limit of the following cosimplicial $\infty$-category

#### Definition 3.56

Let ${\tilde{\mathcal {X}}}:{{\textbf { Nilp}}_{\mathbb {Z}_p\mathrm ft}^{<\infty }}\rightarrow {\textbf {Ani}}$ be the functor sending each $R\in {{\textbf {Nilp}}_{\mathbb {Z}_p,\mathrm ft}^{<\infty }}$ to the anima ${\textrm{Mod}}^{\varphi ,\Gamma }(A_{K,R})^{\simeq }$. By abuse of notation, we still use ${\tilde{\mathcal {X}}}$ to denote the nilcompletion of its left Kan extension along ${{\textbf { Nilp}}_{\mathbb {Z}_p,\mathrm ft}^{<\infty }}\rightarrow {{\textbf { Nilp}}_{\mathbb {Z}_p}^{<\infty }}$, which is called the derived prestack of étale $(\varphi ,\Gamma )$-modules.

Now we proceed to prove ${\tilde{\mathcal {X}}}\simeq \mathcal {X}^{\textrm{nil}}$. As both of them are locally almost of finite type, we just need to compare their evaulation on ${{\textbf { Nilp}}_{\mathbb {Z}_p,\mathrm ft}^{<\infty }}$.

#### Lemma 3.57

Let *R* be an animated ring over $\mathbb {Z}/p^a$. Then for each *n*, we have

$$ (\varinjlim _{\varphi }{\bar{A}}_{K}^{+,n}{\widehat{\otimes }} R)^{\wedge }_{[p]_q,\mathrm derived}\simeq C(\Gamma ^n,A_{{\textrm{cyc}}}/p^a){\widehat{\otimes }} R. $$

#### Proof

It suffices to prove

$$ (\varinjlim _{\varphi }{\bar{A}}_{K}^{+,n}/p^a)/[p]_q\cong C(\Gamma ^n,A_{{\textrm{cyc}}}/p^a)/[p]_q. $$

This follows from $(\varinjlim _{\varphi }{\bar{A}}_K^+)/(p^a,[p]_q)\cong A_{{\textrm{cyc}}}/(p^a,[p]_q)$ and that $\Gamma ^n$ is compact. $\square$

#### Proposition 3.58

Let *R* be a *n*-truncated animated ring which is finite type over $\mathbb {Z}/p^a$. Let $\mathcal {F}_R:\Delta _{\le n+2}\rightarrow {\textbf {Ani}}$ be the natural functor sending each [*i*] to the anima ${\textrm{Vect}}(C(\Gamma ^i,A_{{\textrm{cyc}}}/p^a){\widehat{\otimes }} R[\frac{1}{[p]_q}])^{\varphi =1,\simeq }$ and ${\tilde{\mathcal {X}}}^\textrm{perf}(R)=\varprojlim _{\Delta _{\le n+1}}\mathcal {F}_R$. Then the natural functor ${\tilde{\mathcal {X}}}(R)\rightarrow {\tilde{\mathcal {X}}}^\textrm{perf}(R)$ by base change is fully faithful.

#### Proof

The argument is the same as that in the proof of Proposition 2.24. In particular, we need to use Lemma 3.57 and Remark 2.26 so that we can apply Lemma 2.25 to ${\bar{A}}_K^+$. $\square$

Now in order to prove ${\tilde{\mathcal {X}}}(R)\simeq {\tilde{\mathcal {X}}}^\textrm{perf}(R)$, we need to prove the following result.

#### Proposition 3.59

Let *R* be a *n*-truncated animated ring which is finite type over $\mathbb {Z}/p^a$. Then the following base change functor is an equivalence

$$ {\textrm{Vect}}({\bar{A}}_K^+{\widehat{\otimes }} R[\frac{1}{[p]_q}])^{\varphi =1}\xrightarrow {\simeq } {\textrm{Vect}}(A_{{\textrm{cyc}}}{\widehat{\otimes }} R[\frac{1}{[p]_q}])^{\varphi =1}. $$

Before proving Proposition 3.59, we need to make some preparations when *R* is discrete.

#### Lemma 3.60

Let *R* be a discrete finite type $\mathbb {Z}/p^a$-algebra for some *a*. Then we have the following results.

1. The natural map ${\bar{A}}_{K}^+/[p]_q^n\otimes R\rightarrow A_{{\textrm{cyc}}}/[p]_q^n\otimes R$ is faithfully flat for each *n*. In particular, ${\bar{A}}_{K,R}^+:={\bar{A}}_{K}^+{\widehat{\otimes }} R\rightarrow A_{{\textrm{cyc}},R}$ is faithfully flat.
2. $A_{{\textrm{cyc}},R}$ is exactly the (derived) $[p]_q$-adic completion of $\varinjlim _{\varphi }{\bar{A}}_{K,R}^+$.

#### Proof

For item (1), note that ${\bar{A}}_K^+/p\cong E_K^+\rightarrow A_{{\textrm{cyc}}}/p$ is faithfully flat as $E_K^+$ is a complete discrete valuation ring (cf. [36, Lemma 2.5]) and $A_{{\textrm{cyc}}}/p$ is an integral domain. Then item (1) follows from the proof of [12, Proposition 2.2.12].

For item (2), we first note that $A_{{\textrm{cyc}},R}[[p]_q]=0$. In fact, this follows from $A_{\inf ,R}[[p]_q]=0$ as the natural map $A_{{\textrm{cyc}},R}\rightarrow A_{\inf ,R}$ is faithfully flat by [11, Proposition 2.2.12]. To see $A_{\inf ,R}[[p]_q]=0$, we use the faithfully flat map $\mathfrak {S}_R=(W(k)\otimes R)[[u]]\rightarrow A_{\inf ,R}$ and the fact that $A_{\inf ,R}[[p]_q]=0$ if and only if $A_{\inf ,R}[u]=0$ as $A_{\inf ,R}[\frac{1}{[p]_q}]=A_{\inf ,R}[\frac{1}{u}]$. Now by item (1), we know that ${\bar{A}}_{K,R}^+\rightarrow A_{{\textrm{cyc}},R}$ is injective and so is $\varinjlim _{\varphi }{\bar{A}}_{K,R}^+\rightarrow A_{{\textrm{cyc}},R}$ as $\varphi$-action on $A_{{\textrm{cyc}},R}$ is an isomorphism. In particular, $(\varinjlim _{\varphi }{\bar{A}}_{K,R}^+)[[p]_q]=0$ and the derived $[p]_q$-adic completion of $\varinjlim _{\varphi }{\bar{A}}_{K,R}^+$ is the same as the classical $[p]_q$-adic completion. By derived Nakayama lemma, it suffices to prove $(\varinjlim _{\varphi }\bar{A}_{K,R}^+)/[p]_q\rightarrow A_{{\textrm{cyc}},R}/[p]_q$ is an isomorphism, which follows from $(\varinjlim _{\varphi }{\bar{A}}_K^+)/(p^a,[p]_q)\cong A_{{\textrm{cyc}}}/(p^a,[p]_q)$. $\square$

#### Proof of Proposition 3.59

By Proposition 3.58, we only need to prove this functor is essentially surjective.

We first deal with the case that *R* is discrete. In fact, with the help of Lemma 3.60, we can argue in the same way as [12, Proposition 2.6.9]. In particular, as ${\bar{A}}_K^+$ is $\varphi$-stable, our situation is less involved. We could simply take *T* in loc.cit to be $[p]_q^m$ for some large enough *m* as $\varphi ([p]_q)=[p]_q^p+p\delta ([p]_q)$ and $p^a=0$.

In general, we have the following commutative diagramNote that $\pi _0(A{\widehat{\otimes }} R)=A{\widehat{\otimes }} \pi _0(R)$ for $A={\bar{A}}_K^+, A_{{\textrm{cyc}}}$ by Lemma 3.28. Now by the discrete case, we know the functor $l_4$ is an equivalence. Also by the proof of Proposition 3.31, we see that $l_2$ and $l_3$ are both essentially surjective. Then by Lemma 3.32, we can conclude $l_1$ is essentially surjective. So we are done.

#### Corollary 3.61

Let *R* be a *n*-truncated animated ring which is finite type over $\mathbb {Z}/p^a$. Then we have an equivalence ${\tilde{\mathcal {X}}}(R)\simeq {\tilde{\mathcal {X}}}^\textrm{perf}(R)$.

#### Proof

Just combine Proposition 3.58, Proposition 3.59 and Lemma 2.23. $\square$

Next we will compare ${\tilde{\mathcal {X}}}^\textrm{perf}(R)$ with $\mathcal {X}(R)$.

#### Lemma 3.62

Let *R* be a *n*-truncated animated ring which is finite type over $\mathbb {Z}/p^a$. Then we have

$$ A_{{\textrm{cyc}},R}^n[\frac{1}{[p]_q}]\simeq C(\Gamma ^n,A_{{\textrm{cyc}}}/p^a){\widehat{\otimes }} R[\frac{1}{[p]_q}]. $$

#### Proof

Note that by assumption, $\pi _*(R)=\oplus _n\pi _n(R)$ is a finite type $\mathbb {Z}_p$-algebra. In the setting of Lemma 2.22, we have $K\otimes ^{\mathbb {L}}_{\mathbb {Z}/p^a}\pi _*(R)$ is killed by some bounded power of $\xi$. This means for each *n*, $K\otimes ^{\mathbb {L}}_{\mathbb {Z}/p^a}\pi _n(R)$ is also killed by some bounded power of $\xi$. In particular, we have

$$ A_{{\textrm{cyc}},\pi _n(R)}^n\left[ \frac{1}{[p]_q}\right] \simeq C(\Gamma ^n,A_{{\textrm{cyc}}}/p^a){\widehat{\otimes }} \pi _n(R)[\frac{1}{[p]_q}]. $$

By Lemma 3.28, we know that $\pi _n(A_{{\textrm{cyc}},R}^n[\frac{1}{[p]_q}])\cong A_{{\textrm{cyc}},\pi _n(R)}^n[\frac{1}{[p]_q}]$ and

$$ \pi _n(C(\Gamma ^n,A_{{\textrm{cyc}}}/p^a){\widehat{\otimes }} R\left[ \frac{1}{[p]_q}\right] )\cong C(\Gamma ^n,A_{{\textrm{cyc}}}/p^a){\widehat{\otimes }} \pi _n(R)\left[ \frac{1}{[p]_q}\right] .$$

So we are done. $\square$

As an immediate corollary, we can compare ${\tilde{\mathcal {X}}}^\textrm{perf}(R)$ with $\mathcal {X}(R)$ now.

#### Corollary 3.63

Let *R* be a *n*-truncated animated ring which is finite type over $\mathbb {Z}/p^a$. Then we have

$$ {\tilde{\mathcal {X}}}^\textrm{perf}(R)\simeq \mathcal {X}(R) $$

#### Proof

This follows from Lemma 3.62 and Proposition 3.45. $\square$

Finally we come to the main theorem of this subsection.

#### Theorem 3.64

There is an equivalence of derived prestacks $\mathcal {X}^\textrm{nil}\simeq {\tilde{\mathcal {X}}}$.

#### Proof

As both of them are locally almost of finite type and nilcomplete, this theorem follows from Corollary 3.61 and Corollary 3.63. $\square$

## Data Availability

Data sharing not applicable to this article as no datasets were generated or analysed during the current study.

## Appendix I A lemma on algebraic cotangent complex

The derived deformation theory in [16, Chapter 1] is only established in the characteristic 0 case. However, almost all results in [16, Chapter 1] that we need apply to animated rings straightforwardly. The only place one needs to be careful about is [16, Chapter 1, Lemma 5.4.3], which concerns the topological cotangent complex of $\mathbb {E}_{\infty }$-rings. The goal of this appendix is to establish this result in the setting of animated rings, i.e. consider the algebraic cotangent complex of animated rings.

### Lemma I.1

Let $i:A\rightarrow B$ be a morphism of animiated rings such that $H^0(A)\rightarrow H^0(B)$ is surjective. Consider the corresponding fiber sequence

$$ J\rightarrow A\rightarrow B. $$

Then

1. $$H^0(L_{B/A})=0$$ and $$H^{-1}(L_{B/A})=H^0(B\otimes _AJ)$$;
2. For $n\ge 0$, we have
  $$\begin{aligned} \tau ^{\ge -n}(J)=0\Longrightarrow \tau ^{\ge -n-1}(L_{B/A})=0. ^14 \end{aligned}$$
  ,^14^ In the latter case, $H^0(A)=H^0(B)$ and $H^{-n-2}(L_{B/A})=H^{-n-1}(J)$.

To prove this lemma, let’s recall the following result concerning the Hurewicz map associated with $i:A\rightarrow B$.

### Proposition I.2

[28, Proposition 25.3.6.1] Let $i:A\rightarrow B$ be a morphism of animated rings. Then the Hurewicz map

$$ \epsilon _i: B\otimes _A \textrm{cofib}(i)\rightarrow L_{B/A} $$

is surjective on $H^0$. If the fiber of *i* is connective, then the fiber of $\epsilon _i$ is 2-connective. If the fiber of *i* is *m*-connective for $m>0$, then the fiber of $\epsilon _i$ is $m+3$-connective.

### Proof of Lemma I.1

1. As $H^0(A)\rightarrow H^0(B)$ is surjective, we see that $H^0(\textrm{cofib}(i))=0$. Then $H^0(B\otimes _A\textrm{cofib}(i))=0$. By Proposition I.2, we see that $H^0(L_{B/A})=0$. As *J* is connective, we have $H^{-1}(B\otimes _A\textrm{cofib}(i))\cong H^{-1}(L_{B/A})$ by Proposition I.2. Since $H^{-1}(B\otimes _A\textrm{cofib}(i))$ is just $H^0(B\otimes _AJ)$, we get $H^0(B\otimes _AJ)\cong H^{-1}(L_{B/A})$.
2. Suppose $\tau ^{\ge -n}(J)=0$, i.e. *J* is $(n+1)$-connective. Then by Proposition I.2, we see that $\textrm{fib}(\epsilon _i)$ is $(n+4)$-connective. Hence we have $H^i(B\otimes _A\textrm{cofib}(i))=H^i(L_{B/A})=0$ for all $i\ge -(n+1)$. In particular, as $H^0(J)=0$, we see $H^0(A)=H^0(B)$ and
  $$\begin{aligned}  &   H^{-n-2}(L_{B/A})=H^{-n-1}(B\otimes _AJ)=H^0(B\otimes _AJ[-n-1])\\  &   \quad =H^0(B)\otimes _{H^0(A)}H^0(J[-n-1])=H^{-n-1}(J). \end{aligned}$$

## Footnotes

1 Following [5, Notation 2.1] by a vector bundle over a ringed topos $(\mathcal {T},\mathcal {O})$, we mean an $\mathcal {O}$-module $\mathbb {M}$ such that there exists a cover $\{U_i\}$ of $\mathcal {T}$ and finite projective modules $P_i$ over $\mathcal {O}(U_i)$ such that $\mathbb {M}|_{U_i}\simeq P_i\otimes _{\mathcal {O}(U_i)}\mathcal {O}_{U_i}$. Note that this is different from the definition of finite locally free module over a ringed topos.

2 As the right vertical map is an isofibration, the pullback is the same as homotopy pullback.

3 Here ${\textrm{Hom}}(M,N)$ is the internal Hom object in $\mathcal {D}(A)$ whose connective cover, i.e. $\tau _{\ge 0}{\textrm{Hom}}(M,N)$, gives the mapping space in $\mathcal {D}(A)$, which is an $\infty$-groupoid.

4 To avoid possible confusion, we use $\bar{A}_K^+$ to denote the ring $A_K^+$ defined in [36, Section 2.1] and keep the notation $A_K^+$ for the ring appearing in [12].

5 In the references [11, 12], the completion is always classical completion. So we specify derived completion when needed in this proof.

6 Over a field of characteristic 0, cdga’s, animated rings and $\mathbb {E}_{\infty }$-rings are all equivalent. But this is not true when the base ring is $\mathbb {Z}_p$. In particular, there are two different concepts of cotangent complex. In [27], the cotangent complex for animated rings is called algebraic cotangent complex, which is the notion used in this paper, and the one for $\mathbb {E}_{\infty }$-rings is called topological cotangent complex. One of key lemmas in [16, Chapter 1] is Lemma 5.4.3, which is proved by using [27, Theorem 7.4.3.1] concerning topological cotangent complex. In the setting of animated rings, we need to use [28, Proposition 25.3.6.1], which is somewhat weaker than its $\mathbb {E}_{\infty }$-ring analogue but is still sufficient to prove [16, Lemma 5.4.3].

7 The cotangent complex usually appears in the study of deformation theory of algebraic objects. In our case, the derived prestack $\mathcal {F}:{\textbf {Nilp}}_{\mathbb {Z}_p}\rightarrow {\textbf {Ani}}$ is more like a derived “formal" prestack. So instead, pro-cotangent complex will naturally appear in our context.

8 In [16], the notion of nilcomplete is called convergent.

9 As in [16], nilcomplete is often included in the definition of ind-schemes.

10 Here we still use the homological convention $\tau _{\le n}$.

11 A classical prestack, which admits a cotangent complex and is infinitesimal cohesive, is also called having an obstruction theory.

12 Different to the definition in [38], we do not take sheafification here.

13 As $G_i$ is finite, we simply have $\mathcal {R}_{G_i,{\textrm{GL}}_d}^{c}=\mathcal {R}_{G_i,{\textrm{GL}}_d}$, i.e. all representations are already continuous. For the definition of $\mathcal {R}_{G_i,{\textrm{GL}}_d}$, see [38, Definition 2.2.1].
